# AWMF mold guideline “Medical clinical diagnostics for indoor mold exposure” – Update 2023 AWMF Register No. 161/001 

**DOI:** 10.5414/ALX02444E

**Published:** 2024-05-03

**Authors:** Julia Hurraß, Birger Heinzow, Sandra Walser-Reichenbach, Ute Aurbach, Sven Becker, Romuald Bellmann, Karl-Christian Bergmann, Oliver A. Cornely, Steffen Engelhart, Guido Fischer, Thomas Gabrio, Caroline E.W. Herr, Marcus Joest, Christian Karagiannidis, Ludger Klimek, Martin Köberle, Annette Kolk, Herbert Lichtnecker, Thomas Lob-Corzilius, Norbert Mülleneisen, Dennis Nowak, Uta Rabe, Monika Raulf, Jörg Steinmann, Jens-Oliver Steiß, Jannik Stemler, Ulli Umpfenbach, Kerttu Valtanen, Barbora Werchan, Birgit Willinger, Gerhard A. Wiesmüller

**Affiliations:** 1 Section for Hygiene in Healthcare Facilities, Division of Infection Control and Environmental Hygiene, Cologne Health Department, Cologne,; 2 Formerly: State Agency for Social Services (LAsD) Schleswig-Holstein, Kiel,; 3 Bavarian Health and Food Safety Authority, Munich,; 4 Laboratory Dr. Wisplinghoff,; 5 ZfMK – Center for Environment, Hygiene and Mycology Cologne, Cologne,; 6 Department for Otorhinolaryngology, Head and Neck Surgery, University Medical Center Tübingen, Tübingen, Germany,; 7 Department of Internal Medicine I, Medical University of Innsbruck, Innsbruck, Austria,; 8 Institute of Allergology Charité, Charité – University Medicine Berlin, Berlin,; 9 Institute for Translational Research, CECAD Cluster of Excellence, University of Cologne, Cologne, Germany and Department I for Internal Medicine, Cologne University Hospital, Cologne,; 10 Institute for Hygiene and Public Health, University Hospital Bonn, Bonn,; 11 Baden-Württemberg State Health Office in the Stuttgart Regional Council, Stuttgart,; 12 Formerly: Baden-Württemberg State Health Office in the Stuttgart Regional Council, Stuttgart,; 13 Environmental Health and Prevention, Institute and Polyclinic for Occupational, Social and Environmental Medicine, University of Munich Hospital Ludwig-Maximilians-University, Munich,; 14 Allergological-Immunological Laboratory, Helios Lung and Allergy Center Bonn, Bonn,; 15 Faculty of Health, Professorship for Extracorporeal Lung Replacement Procedures, University of Witten/Herdecke, Witten/Herdecke,; 16 Lung Clinic Cologne Merheim, Clinics of the City of Cologne, Cologne,; 17 Center for Rhinology and Allergology, Wiesbaden,; 18 Department of Dermatology and Allergy Biederstein, School of Medicine, Technical University of Munich, Munich,; 19 Institute for Occupational Safety and Health (IFA) of the German Social Accident Insurance (DGUV), Unit Biological Agents, Sankt Augustin,; 20 Medical Institute for Environmental and Occupational Medicine MIU GmbH Erkrath, Erkrath,; 21 Scientific working group of environmental medicine of the German Society of Pediatric Allergology (GPAU),; 22 Asthma and Allergy Center Leverkusen, Leverkusen,; 23 Institute and Polyclinic for Occupational, Social and Environmental Medicine, member of the German Center for Lung Research, Hospital of the University of Munich, Munich,; 24 Center for Allergology and Asthma, Johanniter Hospital Treuenbrietzen, Treuenbrietzen,; 25 Institute for Prevention and Occupational Medicine of the German Statutory Accident Insurance, Institute of the Ruhr University Bochum (IPA), Bochum,; 26 Center for Pediatrics and Adolescent Medicine, University Hospital Giessen and Marburg GmbH, Giessen,; 27 Specialized Practice in Allergology and Pediatric Pulmonology in Fulda, Fulda,; 28 Institute for Clinical Hygiene, Medical Microbiology and Clinical Infectiology, Paracelsus Private Medical University Nuremberg Clinic, Nuremberg,; 29 Doctor for Pediatrics and Adolescent Medicine, Pediatric Pulmonology, Environmental Medicine, Classical Homeopathy, Asthma Trainer, Neurodermatitis Trainer, Viersen,; 30 FG II 1.4 Microbiological Risks, German Environment Agency, Berlin,; 31 German Pollen Information Service Foundation (PID), Berlin, Germany,; 32 Department of Laboratory Medicine, Division of Clinical Microbiology – Medical University of Vienna, Vienna, Austria, and; 33 Institute for Occupational, Social and Environmental Medicine, Uniclinic RWTH Aachen, Aachen, Germany; a Co-author; b Member of a scientific medical society, a society or a medical association with voting rights

**Keywords:** dampness, mold, indoor, diagnostics, guideline

## Abstract

None.

## Period of validity

- Status of the guideline: September 05, 2023
- Validity of the guideline until: September 04, 2028

## Guideline coordination 

Dr. rer. nat. Julia Hurraß 

## Guideline Steering Group 

Dr. med. Birger Heinzow, Dr. rer. nat. Julia Hurraß (GHUP), Dr. rer. nat. Sandra Walser-Reichenbach (GHUP), Prof. Dr. med. Gerhard A. Wiesmüller (GHUP) 

## Society for Hygiene, Environmental Medicine and Preventive Medicine e.V. (GHUP) 

Prof. Dr. med. Steffen Engelhart, Prof. Dr. med. Caroline E.W. Herr, Dr. rer. nat. Julia Hurraß, Dr. rer. nat. Sandra Walser-Reichenbach, Prof. Dr. med. Gerhard A. Wiesmüller 

## German Dermatological Society e.V. (DDG) 

Dr. rer. nat. Martin Köberle 

## German Society for Allergology and Clinical Immunology e.V. (DGAKI) 

Prof. Dr. rer. nat. Monika Raulf 

## German Society for Occupational and Environmental Medicine e.V. (DGAUM) 

Prof. Dr. med. Dennis Nowak, Prof. Dr. rer. nat. Monika Raulf 

## German Society for Ear, Nose and Throat Medicine, Head and Neck Surgery (DGHNO-KHC) 

Priv.-Doz. Dr. med. Sven Becker 

## German Society for Hospital Hygiene e.V. (DGKH) 

Prof. Dr. med. Steffen Engelhart 

## German Society for Pneumology and Respiratory Medicine e.V. (DGP) 

Prof. Dr. med. Karl-Christian Bergmann, Norbert Mülleneisen, Prof. Dr. med. Dennis Nowak 

## German-speaking Mycological Society e.V. (DMykG) 

Prof. Dr. med. Birgit Willinger 

## Society for Pediatric Allergology and Environmental Medicine e.V. (GPA) 

Dr. med. Thomas Lob-Corzilius 

## Austrian Society for Medical Mycology (ÖGMM) and elected representative 

Prof. Dr. med. Romuald Bellmann, Prof. Dr. med. Birgit Willinger 

## Medical associations and elected representatives: 

### Medical Association of German Allergists (AeDA) 

Prof. Dr. med. Ludger Klimek, Dr. med. Uta Rabe 

### Federal Working Group for Pediatric Pneumology e.V. (BAPP) 

Prof. Dr. Jens-Oliver Steiß 

## Experts 

Dr. med. Ute Aurbach, Prof. Dr. med. Oliver A. Cornely, Dr. rer. nat. Guido Fischer, Dr. rer. nat. Thomas Gabrio, Dr. med. Birger Heinzow, Dr. med. Marcus Joest, Prof. Dr. med. Christian Karagiannidis, Dr. rer. nat. Annette Kolk, Dr. med. Dipl.-Chem. Herbert Lichtnecker, Dr. med. Jannik Stemler, Prof. Dr. med. Jörg Steinmann, Dr. med. Ulli Umpfenbach, Dr. rer. nat. Kerttu Valtanen, Dr. rer. medic. Barbora Werchan 

## Addressees 

The guideline is aimed at allergists, occupational physicians, ENT physicians, hygienists, immunologists, hospital hygienists, medical mycologists, pulmonologists, and environmental medicine specialists and serves to inform general practitioners, family doctors, infectiologists, internists, and pediatricians as well as all other interested medical colleagues and all those affected by indoor moisture/mold damage and affected patients. 

In addition, there is separate information available for persons affected by moisture/mold damage indoors and affected patients. 

## Patient representation 

Due to the complexity of the topic, which affects different medical specialties, it was not possible to identify a suitable higher-level patient or patient-representative body when updating the guideline. Since the guideline was first published, no patient representatives have contacted the guideline group, and despite correspondence with higher-level patient representatives, no one from this group could be persuaded to participate. Only after the consensus conference and the completion of most of this updated version of the guideline the non-profit association “Mukoviszidose e.V. – Bundesverband Cystische Fibrose (CF)” agreed to review the guideline. This meant that at least the association representing the interests of people who suffer from cystic fibrosis and are therefore at increased risk regarding exposure to mold indoors could be included. The suggestions of this interest group to replace the term “CF patients” with “people/persons with cystic fibrosis” and to replace the term “patients” with “people or persons” wherever possible and appropriate were implemented, as well as the suggestion to create a health information, which, like the guideline, can be downloaded from the AWMF website. 

## Consensus strength 

The strength of consensus was determined according to the AWMF guidelines: > 95% = strong consensus, >75% to ≤ 95% = consensus, > 50% to ≤ 75% = majority agreement, ≤ 50% = no majority agreement. 

The consensus strength of the entire guideline is > 95% (strong consensus). 

## 1. About this guideline 

Mold (fungi) indoors is (are) still a major health issue in Germany. 

In a study of dampness and mold in 7,127 homes in 22 centers across Europe (including Germany) with on-site inspections in 3,118 homes, the self-reported incidence of water damage (10%), damp patches (21%), and mold (16%) over the past year was of a similar order of magnitude to observed dampness (19%) and observed mold (14%) [504]. 

The problem is often associated with great uncertainty for those affected. 

The German Environment Agency’s “Guidelines on the prevention, detection and remediation of mold growth in buildings” are available for the detection, assessment, search for causes and remediation of mold growth indoors [297]. Prior to the first publication of the Association of the Scientific Medical Societies in Germany (AWMF) guideline in April 2016 and its present update, the Robert Koch Institute (RKI) Commission “Methods and Quality Assurance in Environmental Medicine” had already commented on the health assessment in 2007 [354]. In addition, answers to common questions on the health risk assessment of indoor mold exposures are available from the Bioaerosols Working Group of the Society for Hygiene, Environmental Medicine and Preventive Medicine (GHUP) [262, 285, 766, 767, 768, 769, 770, 771, 773, 774, 774, 776]. 

Reliable scientific knowledge on the topic of “health effects of mold” is currently still limited in many areas, and only a few doctors have the necessary specialist knowledge about the health effects of mold and precautionary measures against mold exposure. 

This guideline was created 2016 and updated in 2023 with the aim of improving the required factual approach to the problem, which remains difficult due to the sometimes unclear nosology and the lack of a comprehensive diagnostic guideline. 

This updated guideline aims to provide physicians with assistance in advising and treating patients subject to increased mold exposure in a typical indoor scenario – i.e., not in particularly exposed workplaces (colloquially: “mold exposure”) from a medical perspective. Diagnosing and treating mycoses (infections caused by fungi) is no subject of this guideline. 

“Mold” is a collective term for various molds, bacteria, yeasts, and other microorganisms. Mold damage can become visible indoors on infested materials [297]. 

Mold is a natural, ubiquitous part of our environment. A prerequisite for the development and growth of mold is sufficient moisture in the material or on surfaces, and mold growth is therefore favored by high humidity, inadequate ventilation, and cold component surfaces (consequence: condensation due to falling below the dew point). Structural conditions, water damage, rising damp, leaks, accidents, etc. also lead to increased humidity and can promote the formation and growth of mold. Indoor mold can develop and grow on various materials (e.g., wood, wallpaper, cardboard, plastics, rubber, carpets) and in a wide range of temperatures [354, 783]. The development and growth of mold indoors is always a moisture problem and this is the primary cause that must be eliminated to prevent long-term exposure of users. 

Moisture/mold damage, which can result in health-relevant exposure and thus a potential health risk for healthy people, concerns visible or non-visible cases of damage with microbiological colonization, in particular mold infestation with active mold growth (viable) or with dried (non-viable) mold, in which an increased release of mold components (spores, mycelium, etc.) and other biological substances is likely. 

As individual sensitivity and exposure to mold spores vary greatly and other, as yet unmeasurable parameters (e.g., pathogen-associated molecular patterns (PAMP)) probably also play a role, no health-related guideline values (CFU/m^3^) can be defined. A quantitative health risk assessment is not possible. However, due to the potential health risk of moisture/mold damage indoors, such damage should be classified as a concern from a hygiene/preventive perspective [354] and must always be properly remediated. 

Moisture/mold damage (with increased mold and bacterial growth) is associated with very different effects in humans. These include the following symptoms and clinical pictures with correspondingly pronounced exposure:

- Allergies
     – Type I allergy: Allergic rhinoconjunctivitis, bronchial asthma, urticaria
     – Type I and type III allergy: Allergic bronchopulmonary aspergillosis (ABPA), Allergic bronchopulmonary mycoses (ABPM)
     – Type III and type IV allergy: Hypersensitivity pneumonitis (HP, extrinsic allergic alveolitis {EAA}), humidifier lung, farmer’s lung (workplace).
- Atopic eczema
- Susceptibility to infections (chronic bronchitis)
- System mycoses
- Mycotoxicoses
- Toxic alveolitis, Organic Dust Toxic Syndrome (ODTS) (workplace)
- Mucosal irritation, Mucous Membrane Irritation (MMI), occasionally also referred to as Mucous Membrane Irritation Syndrome (MMIS)
- Upper respiratory tract complaints
- Odor effects
- Mood disorders

**Key message 6, section 1.3 Key messages:**

**Exposure to mold can generally lead to irritation of the mucous membranes (mucous membrane irritation, MMI), odor effects, and mood disorders.**

**Key message 7, section 1.3 Key messages:**

**Specific clinical pictures associated with mold exposure relate to allergies and mold infections (mycoses).**

In practice, answering patients’ questions about the health risk associated with the detection of mold in indoor spaces is primarily a medical task. To be able to assess a health risk from molds, the health situation of the exposed person (predisposition) as well as the extent of mold infestation and the release of mold spores or other components (e.g., metabolic products, cell components) indoors (exposure) must be assessed. 

In practice, the focus is on the patient’s medical history and the general and specialist clinical assessment of the patient. The first step is to check whether there are any indications of particular mold exposure indoors, e.g., due to moisture/mold damage, whether there is a predisposition to possible mold effects and whether the symptoms or clinical pictures may be caused by mold exposure. 

According to current knowledge, the most common health problems associated with mold indoors are probably mucous membrane irritation of the eyes and respiratory tract and allergic reactions [262, 464]. 

At very high bioaerosol concentrations, which generally only occur at workplaces and not indoors, serious toxic effects (Organic Dust Toxic Syndrome, ODTS) can be observed [354, 469, 640]. Work-related exposure and the associated clinical pictures are not the subject of this guideline, but are addressed where appropriate. 

Mold growth indoors must be considered a potential health risk from a prevention perspective, even if no quantitative and causal link can be established between the occurrence of individual species and health complaints. From a health point of view, moisture damage and/or mold growth indoors is always a hygiene problem that should not be tolerated – even without health problems. Rather, according to the precautionary principle, the contamination should be minimized and, if possible, eliminated [297, 380, 469]. 

**Key message 1, section 1.3 Key messages:**

**Mold infestation to a relevant extent should not be tolerated indoors for precautionary reasons. To assess the extent of damage, please refer to the “Guidelines for the prevention, detection and remediation of mold infestation in buildings” published by the German Environment Agency [** 297 **].**

The most important preventive measure in the event of indoor mold exposure is to clarify the cause of the moisture/water damage and carry out proper remediation [138, 297, 381, 519]. 

**Key message 2, section 1.3 Key messages:**

**The most important measures in the case of indoor mold infestation are to identify the cause and carry out proper remediation [** 297 **].**

### 1.1 Purpose and aim of the guideline 

The updated guideline is intended to close the existing gap for a rational and efficient medical diagnosis of indoor mold exposure. For the most part, guidelines have existed and continue to exist almost exclusively for the building-related approach to moisture damage [229, 230, 295, 296, 297, 380, 381, 492, 519] and reviews of associated clinical pictures [198, 298, 354, 771, 783]. However, there is no comprehensive diagnostic procedure for patients. 

Workplace-related diseases or specific workplace exposures, oral ingestion of molds or mold components, and diseases caused by yeasts and dermatophytes are not covered by the guideline. 

The scientific literature on mold is very extensive and mostly published in English. In epidemiological studies, domestic exposure is often categorized with the terms “dampness and mold”, i.e., no distinction is made between moisture damage with or without indoor mold. This makes sense as there are no specific health-related markers for quantitative mold exposure. The term “mold” refers to “visible” mold structures, whereby “visible” also includes hidden mold infestation. 

Further definitions can be found in the supplement to this guideline. 

### 1.2 What’s new? 

This updated guideline contains the following new features:

- Updated and more extensive literature research.
- Updated assessment of diseases associated with indoor moisture/mold damage (Table 4 Evidence for the relationship between indoor moisture/mold exposure and disease).
- Evaluation of new diseases associated with indoor moisture/mold damage (Table 4 Evidence for the relationship between indoor moisture/mold exposure and disease).
- Note in the guideline text on diseases for classification in Table 4 Evidence for the relationship between indoor moisture/mold exposure and disease
- More and more clearly formulated core messages.
- Identification of key messages, whether reviewed, modified or newly added in 2023.
- Identification of the strength of consensus for each core message.
- Linking the key messages to the guideline text.
- Updated cross-references to other guidelines in the guideline text.
- Cross-references to diagnostics and therapy in the guideline text on diseases.
- Inclusion of two further risk groups for moisture/mold damage in the interior.
- Presentation of the reasons why a clearly causal cause-effect relationship cannot be established from the simple correspondence of a measured mold exposure and possible health effects.

### 1.3 Key messages of the guideline 

The key messages of the guideline are listed below, which also contain the core recommendations of the guideline. The strength of the recommendation is expressed by the following terms: 

Strong recommendation: “shall”; recommendation: “should”; open recommendation: “may be considered”. 

The problem of indoor mold exposure needs to be objectified.

1. Mold infestation to a relevant extent should not be tolerated indoors for precautionary reasons. To assess the extent of damage, please refer to the “Guidelines for the prevention, detection and remediation of mold infestation in buildings” published by the Federal Environment Agency [297]. modified 2023, consensus strength > 95%
2. The most important measures in case of indoor mold infestation are to identify the cause and carry out proper remediation [297]. checked 2023, consensus strength > 95%
3. For medical indication, indoor mold measurements are rarely useful. As a rule, both a quantitative and a qualitative determination of mold species can be dispensed with in case of visible mold infestation. Rather, the causes of the infestation should be clarified, and then the infestation and primary causes should be eliminated. modified 2023, consensus strength > 95%
4. In medical diagnostics for mold exposure, environmental monitoring of mycotoxins in indoor air and house dust has no indication. new 2023, consensus strength > 95%
5. In medical diagnostics for mold exposure, environmental monitoring of microbial volatile organic compounds (MVOC) in indoor air has no indication. new 2023, consensus strength > 95%
6. Exposure to mold can generally lead to irritation of the mucous membranes (mucous membrane irritation (MMI)), odor effects and mood disorders. checked 2023, consensus strength > 95%
7. Specific clinical pictures associated with mold exposure relate to allergies and mold infections (mycoses). tested 2023, consensus strength > 95%
8. Physicians shall, in cases of a suspected association between indoor moisture/mold damage and conditions for which there is no evidence of such an association (e.g., acute idiopathic pulmonary hemorrhage in children, arthritis, autoimmune diseases, chronic fatigue syndrome (CFS), endocrinopathies, gastrointestinal effects, cancers, airborne mycotoxicoses, multiple chemical sensitivity (MCS), multiple sclerosis, neuropsychological effects, neurotoxic effects, sudden infant death syndrome, renal effects, reproductive disorders, rheumatism, thyroid disorders, sick building syndrome (SBS), teratogenicity, and urticaria), inform affected individuals objectively about the current state of knowledge. modified 2023, consensus strength > 95%
9. Risk groups requiring special protection are:
     a Persons under immunosuppression according to the classification of the Commission for Hospital Hygiene and Infection Prevention (KRINKO) at the RKI [349]
     b Persons with severe influenza
     c Persons with severe COVID-19
     d Persons with cystic fibrosis (CF; mucoviscidosis)
     e Persons with bronchial asthma modified 2023, consensus strength > 95%
10. Individuals who are allergic to mold and those with diseases that weaken the immunological defense system shall be informed about the dangers of mold exposure indoors and about measures to prevent and minimize such exposure. modified 2023, consensus strength > 95%
11. In principle, a large number of mold species can cause sensitization and allergies in case of corresponding exposure. Compared to other environmental allergens, however, the allergenic potential is to be regarded as lower overall [241, 255]. modified 2023, consensus strength > 95%
12. As polysensitized individuals, atopic patients often also have IgE antibodies against molds, although this does not necessarily mean that they are ill. The clinical severity of the allergic reaction does not correlate with the level of the specific IgE titer. modified 2023, consensus strength > 95%
13. The core elements of a type I allergy diagnosis are medical history, skin prick test, determination of specific IgE antibodies, and provocation testing. In case of allergic bronchopulmonary aspergillosis (ABPA), the determination of specific IgG antibodies should also be performed. In the case of extrinsic allergic alveolitis (EAA), only the determination of specific IgG antibodies shall be performed serologically. modified 2023, consensus strength > 95%
14. The detection of specific IgE or a positive reaction in the skin test initially only mean that a specific sensitization to corresponding allergens is present. A clinically relevant allergy only becomes apparent in connection with typical allergic symptoms. modified 2023, consensus strength > 95%
15. A negative result of a skin test or a specific IgE test for molds does not reliably exclude sensitization to molds. The reasons for this include the varying composition and quality of test extracts or the absence of relevant allergens. modified 2023, consensus strength > 95%
16. The determination of specific IgG antibodies in connection with the diagnosis of an immediate-type mold allergy (type I allergy) has no diagnostic significance and shall therefore not be performed. This also applies to the detection of immune complexes, e.g., using the Ouchterlony test. modified 2023, consensus strength > 95%
17. Galactomannan in serum shall only be performed for the diagnosis of suspected invasive pulmonary aspergillosis, otherwise there is no indication in the diagnosis of mold exposure. new 2023, consensus strength > 95%
18. The determination of eosinophil cationic protein (ECP) and β-1,3-D-glucan (BDG) in serum has no indication and shall not be performed in medical diagnostics in case of mold exposure. new 2023, consensus strength > 95%
19. The basophil degranulation test and histamine release (histamine liberation test (HLT)), the basophil activation test (BAT) using flow cytometry and the determination of other mediators (sulfidoleukotriene release test, cellular antigen stimulation test (CAST-ELISA)) are used in special diagnostics, but should not be performed in basic allergy diagnostics. new 2023, consensus strength > 95%
20. Lymphocyte transformation tests (LTT) for molds are not indicated as a diagnostic procedure [355] and shall therefore not be performed. Modified 2023, consensus strength > 95%
21. The whole blood test is not a suitable instrument for detecting mold sensitization and shall therefore not be performed. new 2023, consensus strength > 95%
22. Invasive mold infections are rare and are most likely to occur by inhalation. In practice, of the molds classified in risk groups 2 and 3 according to TRBA 460 [712], *Aspergillus fumigatus* is the most important mycosis pathogen. Individuals with general strong or very strong immune deficiency (according to KRINKO grade 2 and 3 [349]) are predominantly affected. In case of a corresponding disposition, this risk shall be given special attention. modified 2023, consensus strength > 95%
23. Microbiological, immunological, molecular biological and radiological methods are core elements of mold infection diagnostics and shall be used depending on the indication. modified 2023, consensus strength > 95%
24. Human biomonitoring of mycotoxins has no indication in medical diagnostics for indoor mold exposure, and shall therefore not be performed. new 2023, consensus strength > 95%
25. The following diagnostic methods shall not be used for indoor mold exposure because there is insufficient scientific evidence (without claim of completeness): Detection of molds in the blood, determination of IgA antibodies directed against molds, determination of lymphocyte subpopulations, determination of cytokines, determination of oxidative stress, visual contrast sensitivity test (VCS test), tear film break-up time. new 2023, consensus strength > 95%
26. The following diagnostic methods shall not be used for indoor mold exposure due to a lack of medical and scientific evidence (list is non-exhaustive): electroacupuncture according to Voll, bioresonance procedures, pendulum, Vega test, decoder dermography, biotonometry, biotensor, Kirlian photography (plasma print procedure, energetic terminal point diagnosis), regulation thermography according to Rost, auriculodiagnostics, kinesiology, aurascopy, iris diagnostics, cytotoxic blood tests, provocation and neutralization test (PN test). new 2023, consensus strength > 95%

### 1.4 Methodology 

The Society for Hygiene, Environmental Medicine and Preventive Medicine (GHUP) is responsible for the updated guideline, while a GHUP working group (Dr. Heinzow, Dr. Hurraß, Dr. Walser-Reichenbach, Professor Dr. Wiesmüller) was responsible for editorial coordination. The professional societies significantly involved in the consultation and care of patients with diseases caused by molds were approached and asked to send representatives to the working group. Following this procedure, the multidisciplinary guideline group was composed of members/representatives from the above-mentioned scientific medical societies, associations, and doctors’ associations. 

**Search methodology**

As was already the case for the creation of the guideline, a nationwide network of experts from the Society for Hygiene, Environmental Medicine and Preventive Medicine (GHUP) was used to update the guideline. The updated guideline is based on the opinion of the RKI Commission [354], the WHO guideline [783] and the scientific workshops held as part of the GHUP annual conferences (GHUP 2009 – 2012) on the topic of “Molds and health” [19, 20, 21, 22] and the first version of the guideline [777]. 

The updated guideline has been prepared in accordance with the methodological requirements for the development of guidelines for diagnostics and therapy of the AWMF and corresponds to an S2k guideline according to the AWMF’s 3-step concept. The guideline is based on an extensive and systematic literature search, which, however, does not formally meet the requirements of an S2k guideline, as there exist no clinical studies on the topic. It was therefore not possible or appropriate to assign evidence levels for recommendations. 

The literature search for the first version of the guideline was conducted up to December 2014. For the update of the guideline, a new literature search was carried out up to June 2022, using additional search terms (Table 1). 

A conceptually graded Medline search yielded 1,949 literature references in 2014 and 3,145 literature references in 2022, followed by an abstract screening to narrow down the search. No thematically comprehensive or overarching review on the topic of medical diagnostics for indoor mold and moisture exposure was found, but only literature on individual topics, as shown in Table 1. 

Searches were conducted in German using an Internet search engine (Google) and in English in the Medline database (Medical Literature Analysis and Retrieval System Online). PubMed was searched for the update. 

The following publications were used for basic evidence assessments of an association between mold exposure and defined clinical pictures: WHO (2009) [783], Institute of Medicine (IOM), USA (2004) [298], Palatya and Shum (2012) [526], Baxi et al. (2016) [45], Mendell et al. (2011) [449], Mendell and Kumagai (2017) [450], Cailloud et al. (2018) [80] and Mendell et al. (2018) [451]. 

Further guidelines have been taken into account for individual subject areas, primarily on diagnostics. These are listed as cross-references in the respective chapters. 

The articles selected from the search results were read and the evidence was determined based on the AWMF guidelines. 

The following guidelines were used (as of February 28, 2023; listed in alphabetical order): 

Acute myeloid leukemia, acute lymphocytic leukemia, chronic lymphocytic leukemia

- Society for Pediatric Oncology and Hematology (GPOH). S1 guideline Acute myeloid leukemia – AML – in childhood and adolescence, version 2.0. Register number 025 - 031. Status: March 25, 2019. Valid until: March 24, 2024; https://register.awmf.org/de/leitlinien/detail/025-031
- Society for Pediatric Oncology and Hematology (GPOH). S1 guideline Acute lymphoblastic leukemia – ALL – in children, version 7.0. Register number 025 - 014. Status: May 31, 2021. Valid until: May 30, 2026; https://register.awmf.org/de/leitlinien/detail/025-014
- German Society for Hematology and Medical Oncology (DGHO). S3 guideline Diagnostics, therapy and follow-up for patients with chronic lymphocytic leukemia (CLL), version 1.0. Register number 018 - 032OL. Status: March 31, 2018. Valid until: March 30, 2023 (under revision); https://register.awmf.org/de/leitlinien/detail/018-032OL

Allergic rhinitis, rhinoconjunctivitis, rhinosinusitis

- German Society for General Practice and Family Medicine e.V. (DEGAM) and German Society for Ear, Nose and Throat Medicine, Head and Neck Surgery e.V. (DGHNO-KHC). S2k Guideline Rhinosinusitis. Register number 053 - 012. Status: April 07, 2017. Valid until: April 06, 2022 (under revision); https://register.awmf.org/de/leitlinien/detail/053-012
- German Society for Ear, Nose and Throat Medicine, Head and Neck Surgery (DGHNO-KHC) and German Society for General Practice and Family Medicine (DEGAM). S2k guideline Rhinosinusitis. Register number 017 - 049. Status: April 07, 2017. Valid until: April 06, 2022 (under revision); https://register.awmf.org/de/leitlinien/detail/017-049

Asthma

- NVL program of BÄK, KBV, AWMF. S3 guideline National asthma care guidelines, version 4.0. Register number nvl-002. Status: September 07, 2020. Valid until: September 06, 2025 (under revision); https://register.awmf.org/de/leitlinien/detail/nvl-002
- German Society for Pneumology and Respiratory Medicine (DGP). S2k guideline Specialist diagnosis and therapy of asthma, version 3.0. Register number 020-009. Status: March 01, 2023. Valid until: February 29, 2028; https://register.awmf.org/de/leitlinien/detail/020-009
- Society for Pediatric Pneumology e.V. (GPP). S1 guideline Management of difficult and severe asthma in children and adolescents, new registration. Registration number 026 - 027. Registration date: August 01, 2018. Registration date: October 04, 2023. Planned completion: December 31, 2024; https://register.awmf.org/de/leitlinien/detail/026-027

Atopic eczema (atopic dermatitis, neurodermatitis)

- German Dermatological Society e.V. (DDG). S3 guideline Atopic dermatitis (AD) [neurodermatitis; atopic eczema], version 4.2. Register number 013 - 027. Status: June 16, 2023. Valid until: June 15, 2028; https://register.awmf.org/de/leitlinien/detail/013-027

Chronic obstructive pulmonary disease (COPD)

- NVL program of BÄK, KBV, AWMF. S3 guideline National COPD care guidelines, version 2.0. Register number nvl – 003. Status: June 25, 2021. Valid until: June 24, 2026 (under revision); https://register.awmf.org/de/leitlinien/detail/nvl-003

Coronavirus disease 2019 (COVID-19)

- German Society for Internal Intensive Care and Emergency Medicine e.V. (DGIIN), German Interdisciplinary Association for Intensive Care and Emergency Medicine e.V. (DIVI), German Society for Pneumology and Respiratory Medicine e.V. (DGP), German Society for Infectiology e.V. (DGI). S3 guideline Recommendations for the treatment of patients with COVID-19 – Living Guideline, version 8.1. Register number 113 - 001LG. Status: September 12, 2022. Valid until: September 11, 2023; https://register.awmf.org/de/leitlinien/detail/113-001LG

Cystic fibrosis

- Society for Pediatric Pneumology e.V. (GPP), German Society for Pediatric and Adolescent Medicine e.V. (DGKJ). S3 guideline Pulmonary disease in cystic fibrosis: Pseudomonas aeruginosa, version 2.0. Register number 026 - 022. Status: September 27, 2022. Valid until: September 26, 2027: Guideline manuscript submitted for review, revision not yet completed; https://register.awmf.org/de/leitlinien/detail/026-022

Extrinsic allergic alveolitis (EAA)

- *Quirce S, Vandenplas O, Campo P, Cruz MJ, de Blay F, Koschel D, Moscato G, Pala G, Raulf M, Sastre J, Siracusa A, Tarlo SM, Walusiak-Skorupa J, Cormier Y.* Occupational hypersensitivity pneumonitis: an EAACI position paper. Allergy. 2016; *71:* 765-779; doi: 10.1111/all.12866
- *Quirce S, Vandenplas O, Campo P, Cruz MJ, de Blay F, Koschel D, Moscato G, Pala G, Raulf M, Sastre J, Siracusa A, Tarlo SM, Walusiak-Skorupa J, Cormier Y.* Occupational exogenous allergic alveolitis: an EAACI position paper. Allergology. 2018; *41:* 449-469; doi: 10.5414/ALX02042

Functional body complaints

- German Society for Psychosomatic Medicine and Medical Psychotherapy e.V. (DGPM) and German College of Psychosomatic Medicine e.V. (DKPM). S3 guideline Functional body complaints, version 2.0. Register number 051 - 001. Status: July 18, 2018. Valid until: July 17, 2023 (under revision); https://register.awmf.org/de/leitlinien/detail/051-001

Invasive fungal infections

- Onkopedia Guideline – Invasive fungal infections – Diagnostics. Status: 2018

Working Group on Infections in Hematology and Oncology (AGIHO) of the German Society for Hematology and Medical Oncology (DGHO) 

*Ruhnke M, Behre G, Buchheidt D, Christopeit M, Hamprecht A, Heinz W, Heussel CP, Horger M, Kurzai O, Karthaus M, Löffler J, Maschmeyer G, Penack O, Rieger C, Rickerts V, Ritter J, Schmidt-Hieber M, Schuelper N, Schwartz S, Ullmann A, Vehreschild JJ, von Lilienfeld-Toal M, Weber T, Wolf HH.* Diagnosis of invasive fungal diseases in haematology and oncology: 2018 update of the recommendations of the infectious diseases working party of the German society for hematology and medical oncology (AGIHO). Mycoses. 2018; *61:* 796-813; doi: 10.1111/myc.12838, for the Infections in Hematology and Oncology Working Party (AGIHO) of the German Society for Hematology and Medical Oncology (DGHO); https://www.onkopedia.com/de/onkopedia/guidelines/invasive-pilzinfektionen-diagnostik/@@guideline/html/index.html

- Onkopedia Guideline – Invasive fungal infection therapy. Status: 2019

Working Group on Infections in Hematology and Oncology (AGIHO) of the German Society for Hematology and Medical Oncology (DGHO) 

*Ruhnke M, Cornely OA, Schmidt-Hieber M, Alakel N, Boell B, Buchheidt D, Christopeit M, Hasenkamp J, Heinz WJ, Hentrich M, Karthaus M, Koldehoff M, Maschmeyer G, Panse J, Penack O, Schleicher J, Teschner D, Ullmann AJ, Vehreschild M, von Lilienfeld-Toal M, Weissinger F, Schwartz S.* Treatment of invasive fungal diseases in cancer patients – Revised 2019. Recommendations of the Infectious Diseases Working Party (AGIHO) of the German Society of Hematology and Oncology (DGHO). Mycoses. 2020; *63:* 653-682; doi: 10.1111/myc.13082, for the Infections in Hematology and Oncology Working Party (AGIHO) of the German Society of Hematology and Medical Oncology (DGHO); https://www.onkopedia.com/de/onkopedia/guidelines/invasive-pilzinfektionen-therapie/@@guideline/html/index.html

- Onkopedia Guideline – Prophylaxis of invasive fungal infections. Status: 2022

Working Group on Infections in Hematology and Oncology (AGIHO) of the German Society for Hematology and Medical Oncology (DGHO) 

*Stemler J, Mellinghoff SC, Khodamoradi Y, Sprute R, Classen AY, Zapke SE, Hoenigl M, Krause R, Schmidt-Hieber M, Heinz WJ, Klein M, Koehler P, Liss B, Koldehoff M, Buhl C, Penack O, Maschmeyer G, Schalk E, Lass-Flörl C, Karthaus M, Ruhnke M, Cornely OA, Teschner D.* Primary prophylaxis of invasive fungal diseases in patients with haematological malignancies: 2022 update of the recommendations of the Infectious Diseases Working Party (AGIHO) of the German Society for Haematology and Medical Oncology (DGHO), 2022. J Antimicrob Chemother. 2023; *78:* 1813-1826; doi: 10.1093/jac/dkad143, for the Infections in Hematology and Oncology Working Party (AGIHO) of the German Society for Hematology and Medical Oncology (DGHO); https://www.onkopedia.com/de/onkopedia/guidelines/invasive-pilzinfektionen-therapie/@@guideline/html/index.html

Odor

- German Society for Ear, Nose and Throat Medicine, Head and Neck Surgery (DGHNO-KHC). S2k guideline Olfactory and tasting disorders, version 5.0. Register number 017 - 050. Status: May 01, 2023. Valid until: April 30, 2028; https://register.awmf.org/de/leitlinien/detail/017-050

Pneumonia

- German Society for Pneumology and Respiratory Medicine (DGP). S3 guideline Treatment of adult patients with community-acquired pneumonia, version 4.0. Register number 020 - 020. Status: April 24, 2021. Valid until: April 23, 2025; https://register.awmf.org/de/leitlinien/detail/020-020
- German Society for Pediatric Infectiology e.V. (DGPI) and Society for Pediatric Pneumology e.V. (GPP). S2k guideline Management of community-acquired pneumonia in children and adolescents (pCAP), version 1.0. Register number 048 - 013. Status: March 31, 2017. Valid until: March 30, 2022 (under revision); https://register.awmf.org/de/leitlinien/detail/048-013
- German Society for Pneumology and Respiratory Medicine (DGP). S3 guideline Epidemiology, diagnosis and therapy of adult patients with nosocomial pneumonia, version 2.0. Register number 020 - 013. Status: September 11, 2017. Valid until: September 10, 2022 (under revision); https://register.awmf.org/de/leitlinien/detail/020-013

Rheumatoid arthritis

- German Society for Rheumatology (DGRh). S3 guideline Management of early rheumatoid arthritis, version 3.0. Register number 060 - 002. Status: December 18, 2019; Valid until: December 17, 2024; https://register.awmf.org/de/leitlinien/detail/060-002

Sudden infant death syndrome

- German Society for Sleep Research and Sleep Medicine e.V. (DGSM). S1 Guideline Prevention of Sudden Infant Death Syndrome, version 3.0. Register number 063 - 002. Status: November 06, 2022. Valid until: November 05, 2027 (under revision); https://register.awmf.org/de/leitlinien/detail/063-002

Urticaria

- German Dermatological Society e.V. (DDG) and German Society for Allergology and Clinical Immunology e.V. (DGAKI). S3 guideline Classification, diagnosis and therapy of urticaria, version 3.0. Register number 013 - 028. Status: February 01, 2022. Valid until: January 31, 2025; https://register.awmf.org/de/leitlinien/detail/013-028https://register.awmf.org/de/leitlinien/detail/013-028

Diagnostics

- German Society for Pneumology and Respiratory Medicine (DGP). S2k guideline Diagnostics and therapy of adult patients with cough, version 3.0. Register number 020 - 003. Status: January 01, 2019. Valid until: December 31, 2023 (under revision); https://register.awmf.org/de/leitlinien/detail/020-003
- *Kardos P, Dinh QT, Fuchs K-H, Gillissen A, Klimek L, Koehler M, Sitter H, Worth H.* Guideline of the German Society of Pneumology and Respiratory Medicine on the diagnosis and treatment of adult patients with cough. Pneumology. 2019; *73:* 143-180; doi: 10.1055/a-0808-7409; https://www.thieme-connect.de/products/ejournals/abstract/10.1055/a-0808-7409
- *Renz H, Biedermann T, Bufe A, Eberlein B, Jappe U, Ollert M, Petersen A, Kleine-Tebbe J, Raulf-Heimsoth M, Saloga J, Werfel T, Worm M. In vitro* allergy diagnostics. Allergo J. 2010; *19:* 110-128; doi: 10.1007/BF03362255
- *Ruëff F, Bergmann K-C, Brockow K, Fuchs T, Grübl A, Jung K, Klimek L, Müsken H, Pfaar O, Przybilla B, Sitter H, Wehrmann W.* Skin tests for the diagnosis of immediate allergic type reactions. Guideline of the German Society for Allergology and Clinical Immunology (DGAKI) in coordination with the Medical Association of German Allergists (ÄDA), the Professional Association of German Dermatologists (BVDD), the German Dermatological Society (DDG), the German Society for Ear, Nose and Throat Medicine and Head and Neck Surgery (DGHNOKHC), the German Society for Pneumology and Respiratory Medicine (DGP) and the Society for Pediatric Allergology and Environmental Medicine (GPA). Allergo J. 2010; *19:* 402-415; https://dgaki.de/wp-content/uploads/2010/05/Leitlinie_Hauttests-at-immediate-type-reactions2010.pdf
- *Ruëff F, Bergmann K-C, Brockow K, Fuchs T, Grübl A, Jung K, Klimek L, Müsken H, Pfaar O, Przybilla B, Sitter H, Wehrmann W.* Skin tests for the diagnosis of immediate-type allergic reactions. Guideline of the German Society for Allergology and Clinical Immunology (DGAKI). Pneumology 2011; *65:* 484-495; doi: 10.1055/s-0030-1256476; https://www.thieme.de/statics/dokum​e​n​te/thieme/final/de/dokumente/zw_pn​e​u​mologie/Hauttest_Allergie.pdf
- German Society for Allergology and Clinical Immunology e.V. (DGAKI). Sk2 guideline Standardized application of nasal and conjunctival provocation test on allergic diseases of the Upper Airways. Status: May 28, 2020. Planned completion: December 31, 2024; https://register.awmf.org/de/leitlinien/detail/061-009
- *Gonsior E, Henzgen M, Jörres RA, Kroidl RF, Merget R, Riffelmann F-W, Wallenstein G.* Guideline for the performance of bronchial provocation tests with allergens – Part I. German Society for Allergology and Clinical Immunology. Allergo J. 2000; *9:* 193-199. https://dgaki.de/wp-content/uploads/2010/05/Leitlinie_BronchialeProvokationAllergenenTeilA20001.pdf
- *Gonsior E, Henzgen M, Jörres RA, Kroidl RF, Merget R, Riffelmann F-W, Wallenstein G.* Guideline for the performance of bronchial provocation tests with allergens – Part II. German Society for Allergology and Clinical Immunology. Part B: Allergo J. 2001; *10:* 257-264. https://dgaki.de/wp-content/uploads/2010/05/Leitlinie_BronchialeProvokationAllergenenTeilB2001.pdf
- *Gonsior E, Henzgen M, Jörres RA, Kroidl RF, Merget R, Riffelmann F-W, Wallenstein G. *Guideline for the performance of bronchial provocation tests with allergens. Pneumology. 2002; *56:* 187-198; doi: 10.1055/s-2002-20553
- German Society for Occupational and Environmental Medicine (DGAUM). S2k guideline Workplace-based inhalation test (AIT) – specific inhalation challenge (SIC), version 3.1. Register number 002 - 026. Status: 25.01.2021. Valid until: 24.01.2026; https://register.awmf.org/de/leitlinien/detail/002-026

Therapy

- German Society for Allergology and Clinical Immunology e.V. (DGAKI). S2k guideline Allergen immunotherapy for IgE-mediated allergic diseases, version 5.0. Register number 061 - 004. Status: June 30, 2022. Valid until: June 29, 2027. https://register.awmf.org/de/leitlinien/detail/061-004
- *Pfaar O, Ankermann T, Augustin M, Bubel P, Böing S, Brehler R, Eng PA, Fischer PJ, Gerstlauer M, Hamelmann E, Jakob T, Kleine-Tebbe J, Kopp MV, Lau S, Mülleneisen N, Müller C, Nemat K, Pfützner W, Saloga J, Strömer K, Schmid-Grendelmeier P, Schuster A, Sturm GJ, Taube C, Szépfalusi Z, Vogelberg C, Wagenmann M, Wehrmann W, Werfel T, Wöhrl S, Worm M, Wedi B; Commenting participation and process support: Kaul S, Mahler V, Schwalfenberg A.* Guideline on allergen immunotherapy in IgE-mediated allergic diseases: S2K Guideline of the German Society of Allergology and Clinical Immunology (DGAKI), Society of Pediatric Allergology and Environmental Medicine (GPA), Medical Association of German Allergologists (AeDA), Austrian Society of Allergology and Immunology (ÖGAI), Swiss Society for Allergology and Immunology (SSAI), German Dermatological Society (DDG), German Society of Oto-Rhino-Laryngology, Head and Neck Surgery (DGHNO-KHC), German Society of Pediatrics and Adolescent Medicine (DGKJ), Society of Pediatric Pulmonology (GPP), German Respiratory Society (DGP), German Professional Association of Otolaryngologists (BVHNO), German Association of Paediatric and Adolescent Care Specialists (BVKJ), Federal Association of Pneumologists, Sleep and Respiratory Physicians (BdP), Professional Association of German Dermatologists (BVDD). Allergol Select. 2022; *6:* 167-232; doi: 10.5414/ALX02331E

Prevention

- German Society for Allergology and Clinical Immunology e.V. (DGAKI) and German Society for Pediatric and Adolescent Medicine e.V. (DGKJ). S3 Guideline Allergy prevention, version 4.0. Register number 061 - 016. Status: December 07, 2021. Valid until: January 01, 2026 (under revision). https://register.awmf.org/de/leitlinien/detail/061-016

The manuscript of the guideline was updated step by step. 

All authors were asked by email to provide information on possible conflicts of interest online on the AWMF website (https://i​n​t​eressenerklaerung-online.awmf.org). This information was reviewed by three members of the guideline steering group (Dr. rer. nat. Julia Hurraß, Dr. rer. nat. Sandra Walser-Reichenbach and Prof. Dr. med. Wiesmüller, and conflicts of interest of Dr. rer. nat. Julia Hurraß, were reviewed by Dr. rer. nat. Sandra Walser-Reichenbach and Prof. Dr. med. Wiesmüller; conflicts of interest of Dr. rer. nat. Sandra Walser-Reichenbach were evaluated by Dr. rer. nat. Julia Hurraß and Prof. Dr. med. Wiesmüller; conflicts of interest of Prof. Dr. med. Wiesmüller, were reviewed by Dr. rer. nat. Julia Hurraß and Dr. rer. nat. Sandra Walser-Reichenbach). Authors with paid lecturing or training activities as well as authors with participation in a scientific advisory board and paid lecturing or training activities were excluded from management functions (coordination / WG management) (“minor” conflict of interest). Authors who work as consultants or experts, are members of an advisory board and are paid lecturers or trainers were excluded from voting on the key messages (“moderate” conflict of interest). Authors with participation in a scientific advisory board, paid lecturing or training activities and research projects/conducting clinical studies were excluded from the voting on the key messages on a topic-related basis (“moderate” conflict of interest). Authors without information on conflicts of interest were excluded from the voting on all key messages. No conflicts of interest of “high” relevance (ownership interests (patent, copyright, share ownership)) could be identified for any author that would have led to a restriction or exclusion for updating the guideline. 

The interdisciplinary group of members of the guideline process has a sufficiently comprehensive overview of the entire range of topics covered by the guideline so that internal mutual control of any potential conflicts of interest was also ensured. During the entire guideline process, there were no indications of conflicts of interest influencing the guideline content. 

After creating an agreed structure (I), editors and co-authors were appointed for individual sections, who submitted thematically focused contributions. 

From this, a draft text was compiled (II), which was edited twice within the working group on the basis of the comments received in circulation and supplemented by directly requested contributions from proven experts on specific issues (III, IV). The expertise of Dr. Pontus Harten, Kiel, Germany, on the topics of sarcoidosis and rheumatism was included. 

The assessments and recommendations represent a synthesis of the publications identified and used by the authors. 

From this, the editorial working group (Dr. Heinzow, Dr. Hurraß, Dr. Walser-Reichenbach, Prof. Dr. Wiesmüller) produced a manuscript of the guideline by circulation (V). 

This draft guideline was sent to all members at the beginning of March 2023 with a request for comments. Comments from the circulation procedure were documented and a draft (VI) was prepared for the consensus procedure for voting. 

A consensus on the draft and the comments received was reached at the NIH-type consensus conferences on April 21 and May 3, 2023 via a ZOOM conference. For this purpose, the draft with the comments received was made available to all members of the guideline process as a Word document with the official invitation to the conference after a joint appointment had been made in advance of the consensus conference. Members of the guideline process who were unable to attend the conference had sufficient time and opportunity to submit their comments to the guideline steering group beforehand. If non-participating members of the guideline process had no comments, approval of the draft was obtained from these members by e-mail. The first conference was moderated by Dr. med. Roland Suchenwirth as an independent moderator. In this conference, each core message was presented individually to the participants, discussed, finally formulated by consensus, and adopted. The respective votes were taken by roll call. At this conference and the second conference, the remaining entire guideline text was finalized by consensus with regard to spelling, comprehensibility and correct citation. The second consensus conference was moderated by Dr. rer. nat. Julia Hurraß in consultation with Dr. Nothacker (AWMF representative) due to the lack of an independent moderator. 

This version of the guideline (VII), which was approved at the consensus conference, was voted on for the last time by e-mail in a circulation procedure so that members of the guideline process who were unable to attend the consensus conference also had the opportunity to object, make additions, and/or corrections. This final vote was conducted in a one-off, non-anonymized e-mail round and resulted in the final version (VIII) of the guideline. 

At the same time, it was submitted to the executive boards of all participating scientific medical societies, associations and medical associations for authorization and recommendation for adoption. This final authorization was formally completed by September 5, 2023. 

The guideline is published by the GHUP and in the AWMF guideline collection (https://www.awmf.org/leitlinien). Other specialist societies and associations are recommended to adopt the guideline and it will be made available to interested specialist journals and publishers for reprinting. 

A revision of the guideline is planned for 2028. The contact person for this is Dr. rer. nat. Julia Hurraß, ZfMK - Zentrum für Umwelt, Hygiene und Mykologie Köln, Horbeller Straße 18-20, 50858 Köln, e-mail: j.hurrass@wisplinghoff.de, phone: 0221/940505545. 

### 1.5 Structure 

The general introduction is followed by a description of the purpose and aim of this guideline, including a list of the key messages. This is followed by a description of the search methodology, including the guidelines taken into account and the structure of this guideline. The following chapters describe the definition, occurrence and classification of molds, health problems, and diseases caused by molds and a health risk analysis and assessment. This is followed by a discussion of general examination methods, such as medical history and physical examination, and special diagnostic procedures, such as allergological, infectiological, and toxicological examination methods. Diagnostic methods without sufficient scientific evidence or without a medical or scientific basis are also addressed. The present guideline is rounded off with one chapter each on treatment options for health problems and illnesses caused by mold, remediation of living spaces, preventive measures and a description of the connection between social status and moisture/mold infestation. 

The appendix to this guideline contains important definitions. 

## 2. Occurrence, exposure, and health relevance of molds 

### 2.1 Definition and increased occurrence of molds 

**Mold** is a collective term for hyphal and usually also spore-forming small fungi and does not represent a taxonomically defined unit of fungi. 

Molds are a ubiquitous component of our biosphere and are present in outdoor air, indoors and in certain workplaces to varying degrees, depending on the vegetation and anthropogenic sources such as composting plants, recycling plants, biogas plants, garden centers, waste management and agriculture, both seasonally and regionally. 

**Mold-infested (mold-infested materials)** is building material or inventory that was or still is overgrown (colonized) with molds. If not already visible to the naked eye, the determination is made by microscopic detection of a hyphal network and more or less developed conidia or sporangia carriers directly on the affected surfaces of infested material or on a prepared adhesive film tear-off preparation, regardless of whether the molds are still vital/active or already dead [145]. The examination of mold infestation can also be carried out by cultivation. For this purpose, a suspension of the infested material in the form of a dilution series is plated on nutrient media dishes [145]. After the subsequent cultivation, the qualitative and quantitative determination of the molds present is carried out [142]. 

Air testing is often used to confirm the probability of a mold infestation if there is reasonable suspicion of its presence. The mold multiplication units (spores or hyphal fragments) are either impacted directly onto a culture medium dish by active sampling [143] or collected on a filter [141]. This is then analyzed according to ISO 16000-16 [141]. It is also possible to determine the mold spores and hyphal fragments by direct microscopy, e.g., after active collection on an adhesively coated slide [144]. 

The total number of spores (viable and non-viable spores) of molds [144] or only the number of viable spores [142] – determined as colony forming units (CFU) – can be determined. In contrast to the infectious effect, the sensitizing and toxic effect of molds is independent of whether their spores or cell components are viable. The ratio of the total spore count to the CFU count can vary over a range from 1 : 1 to > 10 : 1. Climate influences (temperature and dryness), age, mechanical influences and disinfection measures considerably impair the cultivability of mold spores [205]. 

In addition to molds, other microorganisms such as bacteria (especially actinobacteria [618, 619, 724]) and microorganisms such as mites may be present. In addition to these living and possibly already dead organisms, various metabolic products and cell components can also be detected in the case of moisture damage, such as toxins, endotoxins, allergens, natural “poly sugars” (so-called β-glucans), microbial volatile organic compounds (MVOCs) and fragments of these organisms (e.g., mycelium fragments). As a rule, it is not known which fragments can have a health effect on affected persons in specific individual cases. 

There is no standardized method and no generally recognized evaluation criteria for the routine detection of the accompanying components that occur indoors alongside mold spores. For this reason, **the entirety of all components that can occur in the event of moisture/mold damage are hereinafter referred to as “mold” or “mold infestation”**. This is intended to make it clear that there can be many other components besides mold. 

Mold contamination is a contamination of surfaces or materials by microorganisms or biogenic particles and substances that goes beyond the general background contamination and occurs through direct contact with infested materials or via the air. Mold-infested materials are building materials or fixtures that are colonized with molds, bacteria and/or other microorganisms – regardless of whether the organisms are vital/actively growing in the material, or have already grown and died. This is also referred to as mold damage. 

Mold infestation can be distinguished from contamination with the help of microscopic analyses. In principle, all materials that contain organic matter (nutrients) and **moisture** that can be utilized by microorganisms can be colonized (infested). In contrast to contamination, the growth of microorganisms in the material results in a firmer anchorage of the microorganisms in the material. Metabolites and spores of these microorganisms are actively released into the room air through metabolic activity. In addition, cells (pieces of mycelium) and cell components can spread [297] 

**Moisture damage is** a visible, measurable or perceived consequence of increased water content in interior spaces or building components. 

From a practical point of view, it makes sense to group increased exposure to molds and other factors associated with increased moisture such as yeasts, bacteria (actinobacteria), and mites indoors as **moisture/mold damage**. 

Over 200 different mold species can be found in our immediate living environment, of which around 50 species are common and the others are rare. The different mold species can be associated with specific sources (Table 2). 

The concentration and species composition of molds in outdoor air are highly dependent on the region, season, climatic conditions, weather and local sources. For this reason, the spore concentrations and species composition in suitable outdoor air are used as a reference for the determination of molds indoors. Concentrations of the individual parameters are subject to great temporal and local variability. However, the detection of increased mold concentrations in indoor air is only an indicator of moisture/mold damage. The source of the increased mold concentration must then be determined so that it and its cause can be remedied. This is essential for the proper remediation of damage and for the prevention of health problems for those affected. 

Depending on humidity, temperature and nutrients, certain species have growth advantages and can be associated with certain causes/sources [468, 469]. 

### 2.2 Classification of molds 

In taxonomy, fungi used to be subsumed under plants, but today they represent their own kingdom as fungi. 

Fungi belong to the eukaryotes and have cell walls made of chitin and other glucans, whereas the cell walls of plants consist of cellulose. 

Another important difference to plants is that fungi, as heterotrophic organisms, have no chlorophyll, do not photosynthesize, and must obtain their energy from organic substances of other organisms [267]. In addition, they do not perform active locomotion. 

The nomenclature of the Fungi is binominal, i.e., each organism has a genus and a species name. However, the names of fungi have and are changed relatively frequently due to new findings and taxonomic classifications. Until a few years ago, taxonomic classification was mainly based on morphological characteristics. New taxonomic descriptions are based on a combination of morphology, physiology and genetic characteristics [128, 609, 610]. The use of molecular biological techniques to identify molds has provided new insights into the phylogeny of different mold species. For example, it has been shown that many organisms that were previously grouped together as a single species are morphologically and physiologically almost identical, but are too genetically different to belong to one species. Currently, phylogenetically closely related species are regarded as species complexes (Table 3). 

For example, molecular biology has so far identified 15 species that were previously grouped morphologically under “*Aspergillus versicolor*”, an indicator organism for moisture damage. The species delimitation within this complex has not yet been conclusively clarified. The species of this complex isolated indoors were predominantly identified as *A. jensenii, A. versicolor, A. protuberus,* or *A. creber.* For many species complexes, identification of the molds down to the species level is only possible by molecular biology. For the general, hygienic assessment of molds indoors according to German Environment Agency’s “Guidelines on the prevention, detection and remediation of mold infestation in buildings” [297], the assignment to the *A. versicolor* complex is sufficient. In test reports and expert opinions, the different species of the *A. versicolor* complex (see definitions) can therefore be summarized by stating the identification criteria used or the literature used [208]. 

Aspergilli with asexual and sexual stages previously had a separate name for each stage, e.g., *Eurotium* and *Aspergillus*. There are currently discussions about naming both stages after the asexual form, i.e., *Aspergillus* for the *Eurotium* and *Aspergillus* structures. 

This can lead to communication problems if, for example, medical professionals list indoor mold species in their reports that are named differently according to the new nomenclature and include them in their assessment of possible health problems. In scientific articles and expert reports, the currently valid name for the designation of a mold fungus should therefore be used wherever possible, e.g., instead of *Penicillium notatum*, a name still frequently used in the medical field, the scientifically correct designation *Penicillium chrysogenum* should be used. 

In MycoBank, an online database, the current names and combinations as well as associated data, e.g., descriptions and illustrations, are accessible (https://www.mycobank.org/). 

In medical mycology, however, fungi are classified clinically and independently of taxonomy into dermatophytes, yeasts and molds. Although the DYM system^1^ represents a practicable classification, this classification is misleading and from a biological point of view (taxonomically) incorrect because molds do not represent a taxonomic unit and most “yeasts” (shoot fungi), like dermatophytes, belong taxonomically to the Ascomycota.

^1^Fungi growing on cultures are divided into three groups according to the DYM system used in medical mycology. DYM stands for dermatophytes (D), yeasts (Y), and molds (M) [www.acta-apa.org/journals/acta-dermatovenerol-apa/papers/10.15570/archive/1996/1/Ginter.pdf; https://doi.org/10.1007/978-3-642-72553-1_4] 

Microbiologically, molds should generally be taxonomically indicated as genus (Genus) and species (Species). If only the Latin genus name and then sp. or spp. are given, the species or the individual species have not been further differentiated. 

**2.2.1 Mycotoxins**

Mycotoxins are secondary metabolic products of molds that can have toxic effects on various cell systems of vertebrates in low concentrations (µg/kg food) depending on the type of toxin and consumption habits: Mycotoxins are to be distinguished from the fungal toxins of basidiomycetes. Numerous mold genera (e.g., *Aspergillus*, *Penicillium*, *Fusarium, Alternaria, Stachybotrys*) can form mycotoxins. Mycotoxin formation depends on the species and on environmental factors such as substrate composition, humidity, pH value, light wavelength, and nutrient competition [178]. Mycotoxins are mostly low-molecular compounds, many of which are formed in polyketide metabolism; the fungal toxins of basidiomycetes are usually oligopeptides. 

In general, mycotoxins from indoor-relevant molds can be found in low concentrations (ppt) in house dust [55], in bioaerosols, and on building materials. Mycotoxins can also be present in human blood [31]. However, the mycotoxins detected here (aflatoxins, ochratoxins, citrinin, patulin, various trichothecenes from *Fusarium* species) are only produced by food-relevant molds, but not by indoor-relevant species. The only exception is sterigmatocystin [31], which is formed as an intermediate product in the aflatoxin biosynthesis of “yellow” aspergilli (*A. flavus* group) and can also be formed as an end product by the *A. versicolor* complex and has been detected in house dust [136, 164]. Because the concentration of mycotoxins in food is 100 to 1,000 times higher than in house dust and bioaerosols, it can be assumed that the mycotoxins in the human body were primarily absorbed via the food pathway. Against this background, it is much more likely that possible internal exposure to sterigmatocystin in humans is acquired via the food pathway than via the inhalation pathway (indoor exposure). However, a differentiated human biomonitoring study on this is still lacking. Mycotoxins are not volatile, but occur in the air bound to spores, cell fragments and other particles. 

As mycotoxins arise from secondary metabolism, they have no physiological significance in the metabolism of the fungus according to current knowledge. They are “waste products” that have only acquired an ecological significance in the course of evolution (e.g., the antibiotically or antimycotically active substances inhibit competitors in the biotope). Mycotoxins are generally only found in health-relevant concentrations in food and animal feed if these have been colonized by molds. A distinction must be made between so-called field pests (primarily *Fusarium* species), which produce mycotoxins on crops, and storage pests (*Aspergillus* and *Penicillium*), which synthesize mycotoxins under warm and humid conditions during storage. 

As has been shown in cell culture and animal experiments, mycotoxins trigger cytotoxic effects [177, 630] and have immunomodulatory effects [476]. The cytotoxic effect of some mycotoxins on lung cells depends on their concentration. The data available to date allow the conclusion that the concentrations of most airborne mycotoxins to be expected indoors have no acute toxic effect. Only the most potent toxic compounds, such as the **satratoxins** (trichothecenes) of *Stachybotrys* species, could be present in indoor environments in their active concentrations due to mold-infested materials [176]. Individual studies indicate that the effective concentration of e.g., aflatoxin, which is detectable in house dust but is not produced by indoor-relevant fungal species, is about one order of magnitude (factor 10) lower in cell systems from the lungs (pulmonary uptake, humans) than the effective concentration in kidney cells (oral exposure, animals) [177]. 

However, the maximum expected concentrations of individual mycotoxins in situ (bioaerosols) obviously cannot explain the cytotoxic effects alone. Rather, synergistic effects of different mycotoxins or of mycotoxins with other cell components (e.g., glucans, endotoxins) appear to be responsible for the effect [354]. 

Even taking into account the higher sensitivity of e.g., primary lung epithelial cells (factor 10 compared to immortalized cells, A 549), the expected exposure concentrations in the air are about a factor of 100 below the effect concentrations in the cell culture-based approach [178, 491]. The only exception here are the **satratoxins** (trichothecenes) of *Stachybotrys chartarum*, which could possibly be in the order of magnitude of the effective concentration under extreme exposure conditions (e.g., during indoor refurbishment). It cannot yet be ruled out that aerogenic concentrations reach a magnitude that could be responsible for immunomodulatory effects and thus possibly promote susceptibility to infection or allergy development [543]. 

There is a particular need for research into the possible effects and synergies of various noxious agents such as mycotoxins in combination with LPS (lipopolysaccharides of bacteria, endotoxins), with β-glucans (cell wall components of fungi) or other groups of organisms (e.g., actinobacteria) [538, 686]. 

**2.2.2 Cell wall components, enzymes, and metabolic products**

In addition to mold spores and mycotoxins, other metabolic products and cell components, such as MVOCs, β-glucans, mannans and ergosterol, also play a role in exposures to molds [473, 474], whereby the MVOCs are responsible for the typical odor. 

Ergosterol is a metabolic product (sterol) of yeasts, molds, and edible fungi. It is formed in varying quantities as a membrane component, toxic properties are not known. 

In connection with moisture damage, other microbiological components such as the lysosomal enzyme N-acetyl-β-D-glucosaminidase and lipopolysaccharides of bacteria (LPS, endotoxin) are also increasingly present (e.g., in house dust) [470]. It has not been clarified whether these markers (cell fragments, β-glucan, ergosterol) correlate better with health effects than cultural exposure parameters (CFU) [99, 196, 225, 270, 562, 595, 708]. 

To date, 77 proteins have been described and officially recognized as allergens of molds (excluding dermatophytes and yeasts) (www.allergen.org). The associated protein families differ significantly biochemically and structurally from the allergen families in pollen, food, or animal epithelia [326]. 

The most prominent representatives of mold allergens are [177, 326]:

- Proteases (n = 18, 16 of which were serine proteases),
- ribosomal proteins (n = 9),
- enolases (n = 5),
- dehydrogenases (n = 4),
- thioredoxins (n = 3),
- heat shock proteins (HSP 70/90) (n = 3),
- peroxisomal proteins (n = 2),
- isomerases (n = 2),
- superoxide dismutases (manganese-dependent superoxide dismutase (MnSOD)) (n = 2), and
- flavodoxins (n = 2).

Other mold allergens can be found among the mitogilins, cyclophilins, fibrinogen-binding proteins and proteins with no known biochemical function [326]. A research group led by Olynych [515] demonstrated an immunomodulatory and proinflammatory effect of zymosan. They showed that zymosan leads to increased leukotriene production in mast cells via a dectin-dependent mechanism. 

### 2.3 Health problems and diseases caused by molds 

Epidemiological studies consistently show an association between moisture/mold damage indoors and health effects, especially respiratory complaints, eye, nose and throat irritation, nasal congestion, wheezing, dry cough, sleep disturbance, snoring and fatigue [596, 758]. This guideline is essentially limited to clinical pictures rather than symptoms. 

The respective evidence for associations between moisture/mold damage and the different health effects is summarized in Table 4. 

**Key message 8, section 1.3 Key messages:**

**Physicians shall, in cases of a suspected association between indoor moisture/mold damage and conditions for which there is no evidence of such an association (e.g., acute idiopathic pulmonary hemorrhage in children, arthritis, autoimmune diseases, chronic fatigue syndrome (CFS), endocrinopathies, gastrointestinal effects, cancers, airborne mycotoxicoses, multiple chemical sensitivity (MCS), multiple sclerosis, neuropsychological effects, neurotoxic effects, sudden infant death syndrome, renal effects, reproductive disorders, rheumatism, thyroid disorders, sick building syndrome (SBS), teratogenicity, and urticaria), inform affected individuals objectively about the current state of knowledge.**

Whether mold poses a health risk depends largely on the disposition of the exposed persons. Risk groups requiring special protection are

- Persons under immunosuppression according to KRINKO [349]
- Persons with severe influenza
- Persons with severe COVID-19
- Persons with CF (mucoviscidosis)
- Persons with bronchial asthma

**Key message 9, section 1.3 Key messages:**

**Risk groups requiring special protection are:**

**a) Persons under immunosuppression according to the classification of the Commission for Hospital Hygiene and Infection Prevention (KRINKO) at the Robert Koch Institute (RKI) [349]**

**b) Persons with severe influenza**

**c) Persons with severe COVID-19**

**d) Persons with cystic fibrosis (CF; mucoviscidosis)**

**e) Persons with bronchial asthma**

Causality cannot be established beyond doubt in individual cases between specific mold exposure and specific health complaints and symptoms. 

A clear causal cause-effect relationship cannot be established from the simple correspondence between a measured mold exposure (CFU/m^3^) and possible health effects. The reasons for this are explained in more detail below based on the possible health effects of indoor mold exposure listed above. 

**Infections**

**Figure 1:**
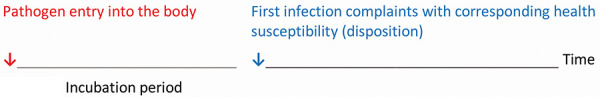
Incubation period [775, 778].

Every infectious disease is preceded by an incubation period. This is the period between the entry of an infectious agent into the human body and the appearance of the first symptoms of infection, i.e., the outbreak of the infectious disease (Figure 1). For example, an incubation period of days to weeks is specified for invasive aspergillosis [775, 778]. 

Since mold is omnipresent, the cause of the mold infestation cannot be determined with certainty based on the mold contamination postulated by an expert in indoor areas with one or more molds at risk of infection according to the Biological Agents Ordinance [278]. The mold fungus may have entered the human body in a different environment, such as in the outside air, at a compost heap, at an organic waste garbage can, in another indoor space, etc. In contrast to bacterial pathogens, a strain identity between the patient isolate and the environmental isolate cannot be proven for molds. 

A prerequisite for a mold infection in humans is a correspondingly pronounced weakness of the immune system (immunosuppression). This health susceptibility (disposition) can only be determined by a doctor based on the assessment of the three risk groups by the KRINKO at the RKI [349]. If there is such a disposition (immunosuppression) and a mold source indoors or in the outside air, immediate cessation of exposure is life-saving and therefore necessary. Affected patients must be informed by their doctor about the necessary measures to avoid exposure. A metrological objectification of mold exposure to one or more molds with risk of infection according to the Biological Agents Ordinance has no benefit for the immediate protection of the immunocompromised person, but carries the risk of a potentially life-threatening prolongation of exposure [775, 778]. 

**Key message 3, section 1.3 Key messages:**

**For medical indication, indoor mold measurements are rarely useful. As a rule, both a quantitative and a qualitative determination of mold species can be dispensed with in the case of visible mold infestation. Rather, the causes of the infestation should be clarified, and then the infestation and primary causes should be eliminated.**

**Key message 10, section 1.3 Key messages:**

**Individuals who are allergic to mold and those with diseases that weaken the immunological defense system shall be informed about the dangers of mold exposure indoors and about measures to prevent and minimize such exposure.**

**Sensitizations and allergies**

**Figure 2:**
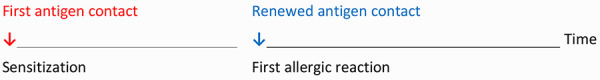
Temporal relationship between sensitization and first allergic reaction [775, 778].

Sensitization in the allergological sense is a misdirected specific immune reaction on first contact with an antigen, which can happen at any time and anywhere. Sensitization is not synonymous with allergic symptoms and does not always lead to an allergy. Further contact with this antigen at a later time can lead to an allergic reaction, i.e., allergic symptoms. The time intervals between sensitization and the first allergic reaction are very variable (Figure 2) [775, 778]. 

Whether exposure to mold leads or has led to sensitization or allergy cannot be proven by measuring molds indoors (CFU/m^3^) [77]. 

However, people who are sensitized to mold fungi may experience allergic reactions due to moisture/mold damage indoors. In this case, too, it is not necessary to objectify the mold exposure by measurement, which is also only insufficiently possible in both qualitative and quantitative terms, but to stop the exposure. This is particularly important for patients with allergic asthma, as in the event of a specific allergic reaction to mold exposure, they can react with an asthma attack, which in the worst case can develop into a life-threatening status asthmaticus (emergency of a particularly severe asthma attack that lasts for a longer period of time and cannot be relieved by the usual asthma treatment). 

Since there are no (valid) commercially available test extracts to detect sensitization, so-called allergy tests, for most molds that occur indoors, neither test results to detect a mold allergy nor the results of mold measurements indoors can be used to determine the allergenic risk of an existing mold infestation in the home of affected people. 

**Key message 3, section 1.3 Key messages:**

**For medical indication, indoor mold measurements are rarely useful. As a rule, both a quantitative and a qualitative determination of mold species can be dispensed with in the case of visible mold infestation. Rather, the causes of the infestation should be clarified, and then the infestation and primary causes should be eliminated.**

**Key message 10, section 1.3 Key messages:**

**Individuals who are allergic to mold and those with diseases that weaken the immunological defense system shall be informed about the dangers of mold exposure indoors and about measures to prevent and minimize such exposure.**

To prevent sensitization and to prevent allergic reactions in the event of existing sensitization, exposure to mold must be stopped (allergen avoidance), i.e., the mold infestation must be remediated [775, 778]. 

**Toxic effects**

There is a more or less long latency period between the intake of a potentially toxic substance and possible health effects. 

**Figure 3:**
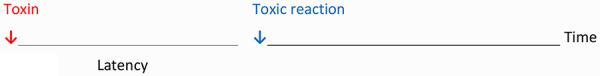
Latency period [775, 778].

This refers to the period between the penetration of a toxin into the human body and the appearance of the first symptoms of intoxication (Figure 3) [775, 778]. 

Exposure to moisture/mold damage is complex and variable. In addition to spores, cell fragments, metabolic products and mycotoxins of molds, bacteria (including actinobacteria), MVOC, β-glucans, mannans, ergosterol, endotoxins, and allergens of the bioaerosol as well as mite allergens are also present. A quantitative health risk assessment for individual components of the bioaerosol is not possible, which is why it is not necessary to determine individual components with regard to a toxic effect when assessing a mold infestation. 

It is known that in the case of moisture damage, some molds produce mycotoxins as secondary metabolites that are bound to spores, mycelium, and cell fragments as components of house dust and bioaerosols, contributing to exposure and presumably to inflammatory/irritative mucosal reactions. 

The concentrations measured to date for mold infestation are very low, so that acute toxic effects (mycotoxicosis) are not to be expected [321]. 

However, it has not yet been clarified which other health effects and consequences are possibly attributable to this in detail. Both antagonistic and synergistic effects have been described in the interaction between the diverse components of the bioaerosol of molds and bacteria. In case of massive moisture damage, the formation of mycotoxins and endotoxins (components of bacteria) must potentially be expected [497]. An increased release of mycotoxins can occur with such dried damage, especially in the course of remediation measures, if large quantities of spores and dust are mobilized. In these cases, strict attention must be paid to the corresponding occupational safety measures [286]. 

Mycotoxin determinations have no significance for the medical assessment of moisture/mold damage. There is currently no indication for the determination of mycotoxins in blood or urine in medical diagnostics for people with indoor mold exposure. The routine determination of mycotoxins in indoor air or in mold-contaminated building materials has neither medical diagnostic significance nor is it necessary for a remediation decision, as any relevant mold infestation must be removed immediately, regardless of whether mycotoxins have been formed or not [775, 778]. 

In principle, all chronic illnesses, even if there is no or insufficient evidence of an association with moisture damage and/or mold exposure, should be treated hygienically. If there are hygienic indications or anamnestic evidence of moisture damage and/or mold (fungus) exposure, the primary causes must always be eliminated preventively, as with all moisture damage. 

**2.3.1 Defined clinical pictures and health disorders**

The different health effects associated with moisture damage and/or mold exposure cannot be explained by a single mechanism or factor [78, 449, 635]. The epidemiological evidence points to both allergologic and non-IgE-mediated immunologic and toxic immunomodulatory mechanisms. In both atopic and non-atopic individuals, moisture damage or mold growth can cause adverse effects [115, 121, 150, 373]. 

The order of the diseases presented below does not represent a weighting of the subject in this guideline. 

**2.3.1.1 Allergic rhinitis**

Climate change, urbanization and industrialization and the associated changes in the indoor and outdoor environment have a significant impact on the prevalence of allergic rhinoconjunctivitis [306]. Type 1 allergic rhinitis is clinically defined as a symptomatic disease of the nose induced by IgE-mediated inflammation of the nasal mucosa following allergen exposure. Allergic rhinitis can be clinically divided into a seasonal, perennial or occupational form, or according to the World Health Organization (ARIA guideline) [66] into an intermittent or persistent form; the severity of the symptoms is also defined on the basis of their severity and the impact on the patient’s quality of life [66, 187]. 

Atopic patients (patients with allergic asthma, allergic rhinitis, atopic dermatitis) are polysensitized and often also have IgE antibodies against molds. 

Depending on the population studied, region and allergen spectrum included, the incidence of allergic rhinitis to fungal allergens is reported at rates of 2.7 – 19% [428, 571, 711]. 

The most common triggers of IgE-mediated rhinitis are allergens from mold species that are mainly found in the outdoor air, namely *Alternaria alternata*, and much less frequently *Cladosporium herbarum*, *Botrytis cinerea*, *Mucor* sp., *Penicillium* sp. and *Aspergillus* sp. [428, 571, 711]. Indoor dampness and mold, as well as exposure at an early age, are consistently associated with allergic rhinitis in epidemiological studies [242, 302, 449, 794]. However, monosensitization to indoor molds is probably rare [455]. 

In summary, it can be stated that there is sufficient evidence for an association between indoor moisture/mold exposure and allergic rhinitis (see Table 4 in chapter 2.3). 

Diagnostic methods are available to a limited extent (see chapter 3). 

Therapeutic procedures are available to a limited extent (see chapter 4). 

**2.3.1.2 Allergic rhinoconjunctivitis**

Allergic symptoms of the nose and eyes (rhinoconjunctivitis) often occur together. Most children with allergic conjunctivitis also have allergic rhinitis. The prevalence of allergic conjunctivitis is estimated to be between 15 and 20% and up to 40% depending on the age of the study [463, 585]. 

Allergic rhinoconjunctivitis is an IgE-mediated inflammatory disease of the nasal mucosa in conjunction with allergy-induced inflammation of the conjunctiva and eyelids. The lifetime prevalence of allergic rhinitis mediated by seasonal allergens is estimated to be ~ 15% in Germany [385]. 

A meta-analysis by Jaakkola et al. (2013) [302] and more recent studies [280, 306, 377, 794] show an association of indoor humidity/mold indicators with the occurrence of allergic rhinoconjunctivitis. 

In summary, it can be stated that there is sufficient evidence for an association between indoor moisture/mold exposure and allergic rhinoconjunctivitis (see Table 4 in chapter 2.3). 

Diagnostic methods are available to a limited extent (see chapter 3). 

Therapeutic procedures are available to a limited extent (see chapter 4). 

**2.3.1.3 Non-invasive and invasive rhinosinusitis**

Rhinosinusitis is divided into an acute form (ARS) and a chronic form (CRS). While in the acute form the symptoms disappear within a maximum of 3 months, in the chronic form they persist for more than 3 months. Pathophysiologically, ARS is caused by a viral infection in the majority of cases; bacterial ARS caused by *Streptococcus pneumoniae*, *Haemophilus influenzae* or *Moraxella catarrhalis* is much rarer [189]. 

With a prevalence of 5 – 12% in the world population, chronic rhinosinusitis is one of the most common chronic diseases in humans. The most common pathogens are *Staphylococcus aureus*, various *Enterobacteriaceae*, less frequently *Pseudomonas aeruginosa* and anaerobes of the oral flora [294]. The European Position Paper on Rhinosinusitis and nasal Polyps 2020 defines CRS as inflammation of the mucous membranes of the nose and paranasal sinuses [189]. By means of imaging or endoscopic examination of the nasal cavities and, if necessary, the sinuses, a distinction can then be made between CRS with (CRSwNP) and without nasal polyps (CRSsNP). In rarer cases, chronic inflammation of the mucous membranes of the nose and nasal sinuses can also be triggered by molds through various mechanisms [186, 187]. Here, the immune status of the host seems to play a decisive role [94]. Sensitizations to molds in patients with chronic sinusitis mainly concern *Alternaria*, a typical mold of the outside air [231]. 

There are currently five different forms of rhinosinusitis caused by fungi:

- the acute invasive form (including rhinocerebral mucormycosis),
- the chronic invasive form,
- the granulomatous invasive form,
- non-invasive (allergic) fungal rhinosinusitis (allergic fungal rhinosinusitis (AFRS)) without and
- with formation of spherical mycetomes [94, 186].

The invasive forms occur more frequently in immunocompromised patients and can lead to death within a few weeks as a fulminant-acute form via vascular hyphal invasion. The most common cause of immunosuppression is untreated or poorly treated diabetes mellitus (50%), followed by underlying hemato-oncological diseases (40%). Other causes can be HIV/AIDS or treatment with chemotherapeutics and biologics [349, 716]. In the chronic invasive form, the progression is protracted, but here too it mainly affects immunocompromised patients. The granulomatous invasive form is a type of fibrotic tumor formation that occurs mainly in Africa, Saudi Arabia, and the Arab Gulf states. The most common causative fungal forms are zygomycetes (*Rhizopus*, *Mucor*, *Rhizomucor*) [189]. 

Three forms of treatment are recommended for the treatment of invasive forms:

- Start systemic antifungal therapy as soon as possible.
- Patients should undergo endoscopic surgical debridement of necrotic sinonasal tissue, repeatedly if necessary.
- If possible, the underlying immunosuppression should be treated.

AFRS is a rather rare immunological reaction to ubiquitous fungi, spores, and their fragments, which usually come into contact with the mucous membranes of the nose and paranasal sinuses through the air. Fungal spores are often found in the nose because they are present in the air we breathe. It is not clear whether molds play a role in all cases of chronic rhinosinusitis, but there is evidence that they may be significant in a subset of patients [250]. 

AFRS is triggered by an IgE-mediated inflammatory reaction to fungal components and is diagnosed using the Bent & Kuhn criteria. 

Clinically characteristic is the presence of a thick, viscous secretion with characteristic histologic findings rich in eosinophilic granulocytes [215]. In the USA, the diagnosis is accepted as confirmed if all the main criteria of the Bent & Kuhn classification [49] are met:

- Type 1 allergy to fungal allergens confirmed by skin testing or *in vitro* testing,
- nasal polyposis,
- characteristic computed tomographic findings,
- presence of eosinophilic mucin without invasion,
- a positive smear test for fungi in the surgically removed paranasal sinus material [49, 215].

Recent studies have shown that fungi are found in the nose and paranasal sinuses in a majority of the population (including all CRS patients) [157]. Thus, it is not only the presence of fungi that seems to be pathognomonic and thus diagnostically indicative, but also a) a reduced immune response in invasive fungal diseases or b) an altered, sometimes exaggerated immune response to these ubiquitous fungal spores in AFRS. 

In the presence of CRS, an examination for IgG4 may make sense if an IgG subclass deficiency is suspected as the underlying cause. However, the available studies are difficult to interpret, so that a determination must be discussed very critically. The determination of IgG4 is by no means the clinical standard [344]. 

Therapeutically, treatment with topical and oral antimycotics is therefore only recommended for invasive forms, but not for AFRS, as double-blind placebo-controlled studies were unable to demonstrate any effect here (see [157]) and no pathophysiological connection with molds is assumed in the majority of cases of chronic rhinosinusitis [188]. 

Severe, untreatable chronic rhinosinusitis can be caused by biofilms, including fungi. The exact pathomechanism is still unclear. It is likely that planktonic fungi are continuously released from the biofilm, and the mucosa is probably invaded by macrophages, which phagocytize the fungal hyphae but do not kill them [107, 191, 192, 320]. 

Mycotic biofilms consist of mold complexes that are able to colonize both biotic and abiotic surfaces. They cause immune evasion and reduced sensitivity to antifungal agents while retaining the ability to release planktonic mold hyphae. Numerous studies using different detection methods have demonstrated the presence of biofilms in the sinonasal mucosa of patients with CRS [107, 191, 192, 320]. The presence of biofilms has been associated with poorer disease outcomes [107]. In patients requiring surgery, the preoperative severity of disease was higher in a group of patients with proven biofilms in the sinonasal mucosa than in a comparison group without evidence of corresponding biofilms, but the postoperative outcome was identical in both groups [192]. 

Confocal laser scanning microscopy with fluorescence in situ hybridization is specified as the “gold standard” detection method for biofilms [320]. This method should be combined with further microbiological examinations. Traditional culture techniques for pathogen detection and identification complement these diagnostic methods [320]. Biofilms are thus an interesting approach to explain the persistence of molds in the chronically inflamed paranasal sinus mucosa. The clinical significance of biofilms for the course of the disease cannot be conclusively assessed at present. In the future, it will be important to develop suitable detection methods for clinical routine. There is no medical indication for the analysis of mycotoxins in nasal secretions or urine. 

In summary, there is sufficient evidence of an association between indoor moisture/mold exposure and non-invasive and invasive rhinosinusitis (see Table 4 in chapter 2.3). 

Diagnostic methods are available (see chapter 3). 

Therapeutic procedures are available (see chapter 4). 

Cross references:

- German Society for General Practice and Family Medicine e.V. (DEGAM) and German Society for Ear, Nose and Throat Medicine, Head and Neck Surgery e.V. (DGHNO-KHC). S2k Guideline Rhinosinusitis. Register number 053 - 012. Status: April 07, 2017. Valid until: April 06, 2022 (under revision); https://register.awmf.org/de/leitlinien/detail/053-012
- German Society for Ear, Nose and Throat Medicine, Head and Neck Surgery (DGHNO-KHC) and German Society for General Practice and Family Medicine (DEGAM). S2k guideline Rhinosinusitis. Register number 017 - 049. Status: April 07, 2017. Valid until: April 06, 2022 (under revision); https://register.awmf.org/de/leitlinien/detail/017-049

**2.3.1.4 Allergic bronchial asthma**

As with allergic rhinitis, mainly molds that occur seasonally in high concentrations in the outdoor air (e.g., mostly *Alternaria*, more rarely *Cladosporium*, *Epicoccum*, *Fusarium*) can induce seasonal allergic bronchial asthma, while indoor molds (*Aspergillus*, *Penicillium*) can lead to perennial allergic bronchial asthma [354, 574]. Olynych et al. (2006) [515] describe that zymosan from molds is able to cause or enhance inflammatory reactions in the airways. The cause is described as increased leukotriene production in the mast cells, which is induced by signals via TLR (Toll-like receptors). The release of leukotrienes can influence lung function and also induce reversible bronchoconstriction and vasodilation with mucosal edema. Results from Vanderborght et al. (2021) [731] showed that type 2-high asthma can be associated with a specific indoor mycobioma. *Aspergillus versicolor* and *Cladosporium spherospermum* have been detected as asthma-relevant indoor species. An association between damp indoor environments (dampness) and/or molds and the development of asthma, especially in children, can be considered certain [46, 86, 240, 299, 318, 449, 539, 654, 700, 707]. Rising global temperatures can promote the growth of known allergenic fungi. Mold in living spaces is not only to be expected in winter due to the cooler temperatures, but also in summer if there is sufficient humidity. 

The genus *Alternaria alternata* (formerly *A. tenuis*), which is considered an outdoor mold, appears to be a particularly important mold for the development and severity of asthma [243, 396, 452, 628, 798]. A temporal relationship between asthma symptoms and spore flight was shown, particularly in patients with a high degree of sensitization and in patients with and without concurrent grass pollen allergy. Other authors have emphasized the importance of *Cladosporium* spp. for allergic asthma, which occurs seasonally in very high concentrations in the outdoor air, but also in indoor infestations [87, 422, 423, 424, 425, 629]. In patients with seasonal asthmatic symptoms (June to September), *Alternaria* sensitization may also be present without simultaneous pollen sensitization [627]. 

Allergic bronchial asthma is often associated with other atopic diseases (atopic dermatitis, allergic rhinoconjunctivitis) [35, 36, 80, 354, 456, 576, 676]. Monosensitization to indoor molds is rare. Clinical studies show that polyvalent sensitization to other environmental allergens is common with molds [324]. It should always be borne in mind that other microorganisms such as actinomycetes and mites, as further allergen sources, also reproduce well under the optimal living conditions for molds, so that trigger factors may be present here. Up to 95% of asthmatics allergic to molds are also sensitized to other inhalation allergens. As weak allergens, mold allergens rarely induce isolated allergies and usually only occur in patients with a high sensitization potential, and a genetic predisposition is important for this sensitization process in addition to mold exposure in damp homes [300, 706]. 

In summary, it can be stated that there is sufficient evidence for an association between indoor moisture/mold exposure and the manifestation, progression, and exacerbation of allergic bronchial asthma (see Table 4 in chapter 2.3). 

Diagnostic methods are available to a limited extent (see chapter 3). 

Therapeutic procedures are available to a limited extent (see chapter 4). 

Cross references:

- NVL program of BÄK, KBV, AWMF. S3 guideline National asthma care guidelines, version 4.0. Register number nvl-002. Status: September 07, 2020. Valid until: September 06, 2025 (under revision); https://register.awmf.org/de/leitlinien/detail/nvl-002
- German Society for Pneumology and Respiratory Medicine (DGP). S2k guideline Specialist diagnosis and therapy of asthma, version 3.0. Register number 020-009. Status: March 01, 2023. Valid until: February 29, 2028; https://register.awmf.org/de/leitlinien/detail/020-009
- Society for Pediatric Pneumology e.V. (GPP). S1 guideline Management of difficult and severe asthma in children and adolescents, new registration. Registration number 026 - 027. Registration date: October 04, 2023. Planned completion: December 31, 2024; https://register.awmf.org/de/leitlinien/detai​l​/​0​2​6​-027

**2.3.1.5 Atopic eczema (atopic dermatitis, neurodermatitis)**

As aeroallergens, mold allergens can presumably be triggers for atopic dermatitis [722, 755]. Epidemiological studies have shown an association between (atopic) eczema and moisture damage/mold. Whether this association represents an immunologically mediated reaction of the skin to indoor exposure to molds has not been clarified [337, 759]. 

A case report suggests that occupational contact urticaria and protein contact dermatitis with subsequent eczema development against *Penicillium* can also be an expression of immunologically mediated dermatitis in mold sensitization in connection with other mold exposures [760]. Sensitization alone often does not justify avoidance or therapeutic measures [386]. 

In summary, it can be stated that there is limited or suspected evidence of an association between indoor moisture/mold exposure and the manifestation of atopic eczema (atopic dermatitis, neurodermatitis) (see Table 4 in chapter 2.3). 

Diagnostic methods are available to a limited extent (see chapter 3). 

Therapeutic procedures are available to a limited extent (see chapter 4). 

Cross references:

- German Dermatological Society e.V. (DDG). S3 guideline Atopic dermatitis (AD) [neurodermatitis; atopic eczema], version 4.2. Register number 013 - 027. Status: June 16, 2023. Valid until: June 15, 2028; https://register.awmf.org/de/leitlinien/detail/013-027

**2.3.1.6 Urticaria**

In rare cases, the consumption of food contaminated with mold components can trigger urticaria [36, 456]. Examples are mold components (such as enzymes) in beverages, in baked goods or on dried sausage/salami [272, 421, 464]. Airborne exposure as a trigger of urticaria is unlikely [526] or a rarity [646]. 

In summary, it can be stated that there is inadequate or insufficient evidence of an association between indoor moisture/mold exposure and urticaria (see Table 4 in chapter 2.3). 

Cross references:

- German Dermatological Society e.V. (DDG) and German Society for Allergology and Clinical Immunology e.V. (DGAKI). S3 guideline Classification, diagnosis and therapy of urticaria, version 3.0. Register number 013 - 028. Status: February 01, 2022. Valid until: January 31, 2025; https://register.awmf.org/de/leitlinien/detail/013-028

**2.3.1.7 Hypersensitivity pneumonitis (HP), extrinsic allergic alveolitis (EAA)**

An association between hypersensitivity pneumonitis (HP) (extrinsic allergic alveolitis (EAA)) in susceptible individuals and the presence of molds is documented by clinical evidence [219, 298]. With a prevalence of 2 – 4 cases per 100,000 inhabitants per year, HP is a rare allergic disease (type III, IV) against inhalation antigens [233, 584]. Molds and other microorganisms from indoor sources play a major role in this disease (Table 5). 

The antigens are contained in dusts and aerosols; possible microbially contaminated sources include birds, feathers, hay, wood dust, humidifiers, air conditioners, indoor fountains, aquariums, steam irons [147, 477, 638] and wind instruments [124]. The antigens most frequently originate from birds, molds and actinomycetes [475]. In EAA, non-smokers are predominantly affected. A comprehensive catalog of antigens has been compiled by Sennekamp [641]. 

EAA was first described in farmers in the 1930s as farmer’s lung [41] and still occurs frequently in the workplace today [508, 714]. It is classified as an occupational disease (In Germany: occupational diseases no. 4201). Numerous literature references describe cases and case series with non-workplace-related EAAs [29, 165, 304, 388, 532, 704] of which feather duvet alveolitis in particular has been diagnosed more frequently in recent years [362]. In Central Europe, the dominant disease pattern is bird-related hypersensitivity pneumonitis [637, 638, 639]. 

In more recent diagnostic criteria, a distinction is made between an acute and a chronic form [558]. This was not the case at the time of the German recommendations for the diagnosis of HP from the Extrinsic allergic Alveolitis Working Group in 2006 [637]. 

In summary, it can be stated that there is sufficient evidence for an association between indoor moisture/mold exposure and HP/EAA, (see Table 4 in chapter 2.3). 

Diagnostic methods are available (see chapter 3). 

Therapeutic procedures are available (see chapter 4). 

Cross references:

- *Quirce S, Vandenplas O, Campo P, Cruz MJ, de Blay F, Koschel D, Moscato G, Pala G, Raulf M, Sastre J, Siracusa A, Tarlo SM, Walusiak-Skorupa J, Cormier Y*. Occupational hypersensitivity pneumonitis: an EAACI position paper. Allergy. 2016; *71:* 765-779; doi: 10.1111/all.12866
- *Quirce S, Vandenplas O, Campo P, Cruz MJ, de Blay F, Koschel D, Moscato G, Pala G, Raulf M, Sastre J, Siracusa A, Tarlo SM, Walusiak-Skorupa J, Cormier Y.* Occupational exogenous allergic alveolitis: an EAACI position paper. Allergology 2018; *41:* 449-469; doi: 10.5414/ALX02042

**2.3.1.8 Allergic bronchopulmonary aspergillosis (ABPA)**

Allergic bronchopulmonary aspergillosis (ABPA) is the most common pulmonary aspergillosis with up to 125 cases per 100,000 inhabitants in Germany. Around 2.5% of all asthma patients and 8% of patients with CF develop ABPA in the course of their lives [310]. Allergic mycosis due to other fungi (ABPM) is much rarer and a large number of different triggers have been described [103]. 

The cause is inhalation-acquired colonization with *Aspergillus* spores, which in ABPA always triggers a type 1 immune reaction with greatly increased total IgE and increased specific IgE antibodies against *Aspergillus* and its components, often also with an additional type 3 reaction with detection of specific *Aspergillus* IgG antibodies [4, 657]. The risk of developing ABPM may increase with high fungal concentrations in the home environment [517, 529, 581], but other authors see no definite connection here [612]. 

Clinically, ABPA presents with coughing, shortness of breath, worsening asthma, hemoptysis, and thick mucus leading to mucus adhesions. The diagnosis of ABPA in asthma is based on the original findings of Rosenberg et al. [586] (Table 6a), which have recently been somewhat simplified (Table 6b) [616]. 

Separate criteria exist for ABPA in cystic fibrosis (CF-ABPA) [288, 675]. Mandatory for all criteria are a total IgE > 417 or 500 kU/L and the detection of an *Aspergillus* IgE antibody [586, 616]. IgE antibodies against the *Aspergillus* components Asp f 1, 2, 4, and 6 can increase sensitivity and specificity [415, 480]. 

The therapy consists mainly of oral steroids [454]. In addition, antifungal therapy (best data available for itraconazole) or treatment with a biologic (anti-IgE, anti-IL4/13, or anti-IL-5) can reduce the need for steroids [310]. Early diagnosis and treatment is important as ABPA can lead to progressive fibrotic remodeling of the lung if left untreated [288, 348, 453, 675]. 

In summary, it can be stated that there is sufficient evidence for an association between indoor moisture/mold exposure and ABPA (see Table 4 in chapter 2.3). 

Diagnostic methods are available (see chapter 3). 

Therapeutic procedures are available (see chapter 4). 

**2.3.1.9 Mycoses**

Infections caused by molds from the environment are called exogenous mycoses (Table 7). Diagnosis and treatment of mycoses are not the subject of this guideline, but only the risk assessment of a risk of infection from exposure to indoor molds, as patients with an increased risk should be advised individually by a doctor about the consequences and preventive measures. 

Mold infections have increased in recent years [254, 591]. A high incidence is found in particular in hemato-oncology patients with a prolonged neutropenic phase and in recipients of an allogeneic stem cell transplant. However, other immunosuppressions, such as prolonged corticosteroid use and pulmonary scaffolding diseases (including scarred residual conditions, e.g., after tuberculosis [18, 663]) and the combination of these factors, particularly in COPD [238, 249], have been linked to increased rates of mold infections. Patients with acute respiratory failure due to influenza [8, 114, 281, 406, 427, 648] or COVID-19 [8, 37, 174, 541, 588, 613, 615, 733, 749] can suffer an invasive mold infection. 

Hematology and oncology patients are now often treated for longer periods due to improved treatment options. However, this often leads to a longer-lasting risk of infection, including repeated neutropenia phases. There is also a tendency to move chemotherapy to outpatient settings and private homes [179]. This can lead to increased exposure in the home during and/or immediately after chemotherapy. Mold infections are among the most common causes of death from infectious diseases in hemato-oncology patients and are becoming increasingly important [254]. When mold mycoses develop in susceptible patients, they are usually acquired via the respiratory tract. Primary sites of infection are most frequently the lungs, less frequently the paranasal sinuses, the ear or traumatized skin. Starting from the respiratory tract, the molds can spread hematogenously or lymphogenously and thus infect other organs [469]. 

Thermotolerant *Aspergillus* species only rarely occur indoors in elevated concentrations (possibly in flower pots), but can be introduced indoors, e.g., in the immediate vicinity of compost or waste treatment plants, but also due to other anthropogenic influences (e.g., agricultural activities). 

Infections caused by opportunistic molds (mesophilic “environmental” species) have been described sporadically in the literature [1, 2, 436, 486, 561, 710]. In a recent evaluation of a total of 53 outbreaks of aspergillosis with 458 affected patients, *Aspergillus fumigatus* and *Aspergillus flavus* were identified as the most common species. Over 50% of the affected patients came from the hematology/oncology department. 

In hospitals, (nosocomial) mold infections are mainly caused by inhaled spores of *Aspergillus* and *Mucor* through contaminated material, construction work or potted plants. Nosocomial infections are defined by the diagnosis of an infection > 48 hours after hospital admission. Immunosuppression usually occurs later after several days of chemotherapy. However, inhalation of spores can occur earlier and even before hospitalization. Spores can persist on the mucous membrane (e.g., in the paranasal sinuses) and only cause an infection after immunosuppression. This probably explains why infections occur even with maximum isolation and e.g., HEPA air filtration. Mold infections can also occur outside the hospital, as case reports show [48, 97, 106, 666, 729]. Chen et al. [97] observed an increase in community-acquired mold infections in their studies on pulmonary mold infections in Taiwan. The connection with construction sites and demolition work and the resulting increase in mold spore contamination of outdoor and (secondarily) indoor air is considered certain [751]. 

In all reports on mold infections, it must be taken into account that it is not clear whether these infections were acquired outside the hospital indoors and/or outdoors**.**

**Key message 7, section 1.3 Key messages:**

**Specific clinical pictures associated with mold exposure relate to allergies and mold infections (mycoses).**

**Key message 22, section 1.3 Key messages:**

**Invasive mold infections are rare and are most likely to occur by inhalation. In practice, it is not possible to distinguish between those in risk groups 2 and 3 according to TRBA 460 [712], the relevance of** *Aspergillus fumigatus* **as the most important mycotic agent is the highest. Individuals with general strong or very strong immune deficiency (according to KRINKO grade 2 and 3 [349]) are predominantly affected. In case of a corresponding disposition, this risk shall be given special attention.**

In summary, it can be stated that there is sufficient evidence for an association between indoor moisture/mold exposure and mycoses (see Table 4 in chapter 2.3). 

Diagnostic methods are available (see chapter 3). 

Therapeutic procedures are available (see chapter 4). 

2.3.1.9.1 Invasive aspergillosis 

Invasive *Aspergillus* infections are a significant cause of morbidity and mortality in immunosuppressed or immunodeficient patients [173, 276]. There is insufficient data on the incidence of aspergillosis in Germany; the global incidence is estimated at 300,000 infections per year [56]. It is a disease with a high mortality rate (case fatality rate 30 – 90% depending on the patient population and spread of infection) [400, 732]. 

Moderately to very severely immunocompromised patient groups include, in particular, hematology patients following stem cell transplantation or intensive chemotherapy. In addition to general hospital hygiene recommendations, special measures to prevent invasive mold infections apply to these patients, such as avoiding gardening, certain spore-contaminated foods, or the conditional use of HEPA filters in patient rooms [349]. 

They should be examined regularly for signs of an invasive mold infection. Furthermore, recommendations for drug-based antifungal prophylaxis apply to certain patient collectives [447]. 

For the diagnosis and management of (angio-)invasive bronchopulmonary aspergillosis (IPA), please refer to the joint guideline of the German-speaking Mycological Society (DMyKG) and the Paul Ehrlich Society (PEG), which is currently being prepared, as well as the guidelines for the diagnosis and treatment of “invasive fungal infections” in accordance with the recommendations of the Infection Working Group of the German Society for Hematology and Oncology. 

**Key message 22, section 1.3 Key messages:**

**Invasive mold infections are rare and are most likely to occur by inhalation. In practice, it is not possible to distinguish between those in risk groups 2 and 3 according to TRBA 460 [712], the relevance of** *Aspergillus fumigatus* **as the most important mycotic agent is the highest. Individuals with general strong or very strong immune deficiency (according to KRINKO grade 2 and 3 [349]) are predominantly affected. In case of a corresponding disposition, this risk shall be given special attention.**

In summary, it can be stated that there is sufficient evidence of an association between indoor moisture/mold exposure and invasive aspergillosis (see Table 4 in chapter 2.3). 

Diagnostic methods are available (see chapter 3). 

Therapeutic procedures are available (see chapter 4). 

Cross references:

- *Ruhnke M, Behre G, Buchheidt D, Christopeit M, Hamprecht A, Heinz W, Heussel CP, Horger M, Kurzai O, Karthaus M, Löffler J, Maschmeyer G, Penack O, Rieger C, Rickerts V, Ritter J, Schmidt-Hieber M, Schuelper N, Schwartz S, Ullmann A, Vehreschild JJ, von Lilienfeld-Toal M, Weber T, Wolf HH.* Diagnosis of invasive fungal diseases in haematology and oncology: 2018 update of the recommendations of the infectious diseases working party of the German society for hematology and medical oncology (AGIHO). Mycoses 2018; *61:* 796-813; doi: 10.1111/myc.12838, for the Infections in Hematology and Oncology Working Party (AGIHO) of the German Society for Hematology and Medical Oncology (DGHO); https://www.onkopedia.com/de/onkopedia/guidelines/invasive-pilzinfektionen-diagnostik/@@guideline/html/index.html
- *Ruhnke M, Cornely OA, Schmidt-Hieber M, Alakel N, Boell B, Buchheidt D, Christopeit M, Hasenkamp J, Heinz WJ, Hentrich M, Karthaus M, Koldehoff M, Maschmeyer G, Panse J, Penack O, Schleicher J, Teschner D, Ullmann AJ, Vehreschild M, von Lilienfeld-Toal M, Weissinger F, Schwartz S.* Treatment of invasive fungal diseases in cancer patients - Revised 2019. Recommendations of the Infectious Diseases Working Party (AGIHO) of the German Society of Hematology and Oncology (DGHO). Mycoses 2020; *63:* 653-682; doi: 10.1111/myc.13082, for the Infections in Hematology and Oncology Working Party (AGIHO) of the German Society of Hematology and Medical Oncology (DGHO); https://www.onkopedia.com/de/onkopedia/guidelines/invasive-pilzinfektionen-therapie/@@guideline/html/index.html

2.3.1.9.2 Aspergilloma and other chronic pulmonary aspergillosis 

Aspergilloma (mycetoma, fungal tumor) is a localized form of chronic pulmonary aspergillosis (CPA). It usually develops in preformed cavities (sinus, lung) due to an accumulation of mold mycelia and forms a typical spherical structure. Predisposing factors include caverns after tuberculosis, bronchiectasis, and malignant diseases [536]. 

While aspergilloma describes the classic, usually singular manifestation, the far more common clinical picture of chronic cavitary pulmonary aspergillosis (CCPA) includes such lesions in one or more sections of the lung with respiratory and/or systemic symptoms persisting for more than three months. 

The entity of chronic fibrosing pulmonary aspergillosis (CFPA) describes a fibrotic change in at least two lung segments that leads to a measurable reduction in lung function. 

In contrast to invasive aspergillosis, aspergillomas, CCPA and CFPA usually occur in immunocompetent or only slightly immunosuppressed people. 

Imaging diagnostics using CT thorax is the most important examination method for detecting and differentiating pulmonary aspergillosis. In the classic presentation with a fungal ball in a preformed cavity, only the detection of elevated *Aspergillus*-specific IgG antibodies is required to confirm the diagnosis according to the ERS guideline from 2016 [132, 723]. The problem of a suitable cut-off value for this is pointed out in a recent CPAnet publication [664]. In addition, the direct detection of *Aspergillus fumigatus* or its components in sputum, bronchial secretions / bronchoalveolar lavage (BAL) or biopsy material by means of culture or PCR may be necessary to confirm the diagnosis. 

In summary, there is sufficient evidence of an association between indoor moisture/mold exposure and aspergilloma and other CPA (see Table 4 in chapter 2.3). 

Diagnostic methods are available (see chapter 3). 

Therapeutic procedures are available (see chapter 4). 

**2.3.1.10 Organic dust toxic syndrome (ODTS)**

Organic dust toxic syndrome (ODTS) is a flu-like acute systemic disease caused by inhalation of high concentrations of bioaerosols, which occur almost exclusively in the workplace. It is sometimes difficult to distinguish from EAA [147, 469, 636]. Local and systemic reactions are possible, with ODTS showing a regular dose dependency. A decision-making aid in the differential diagnosis of EAA vs. ODTS can be found in Table 8. 

ODTS symptoms have been described with very high bioaerosol exposures. Exposure to large quantities of dust with an extreme spore load (> 10^9^ spores/m^3^, possibly less in the case of *Aspergillus fumigatus*) [260] can lead to asthma and pneumonitis [426], with symptoms similar to extrinsic allergic alveolitis. Continued exposure can lead to granulomatous scarring and pulmonary fibrosis [260, 594]. The exact cause of the toxic-irritative effects is not known in detail [354, 381]. 

In summary, it can be stated that there is sufficient evidence for an association between indoor moisture/mold exposure and ODTS (see Table 4 in chapter 2.3). 

Diagnostic methods are available (see chapter 3). 

Therapeutic procedures are available (see chapter 4). 

**2.3.1.11 Pulmonary hemorrhage, acute idiopathic pulmonary hemosiderosis**

Acute idiopathic pulmonary hemosiderosis (AIPH) is a very rare disease whose etiology is unknown [265]. 

In the late 1990s and early 2000s, reports of individual cases [163, 184, 764] and a cluster in Cleveland [168] found a possible link between acute pulmonary hemorrhage in children and exposure to mold. Individual air and dust analyses in homes of children with pulmonary hemorrhage could not provide reliable evidence for this association [744, 745] and initial doubts arose about a possible link between acute pulmonary hemorrhage in children and exposure to mold [54]. 

In 2000, the U.S. Centers for Disease Control and Prevention (CDC) [88] published a statement that there was no evidence of an association between acute pulmonary hemorrhage in newborns and exposure to mold. Previously, an association between pulmonary hemorrhage and indoor mold exposure describing a cluster in 30 newborns in Cleveland [126, 168] was postulated. However, these studies exhibited several methodological flaws with regard to the collection, analysis and evaluation of the data [369]. 

Another cluster of pulmonary hemorrhage in four newborns was described by the CDC in 2004 [90]. Although the newborns were exposed to various environmental factors, including mold, the CDC did not see a direct causal link to the development of pulmonary hemorrhage due to the lack of a comparison population. 

Nevertheless, such an association is still postulated in reviews [170, 171]. 

In its report “Dampness and Mould” [783] publications up to and including 2007 WHO refers to the cluster described in Cleveland and cites the Institute of Medicine (IOM) [298], which sees no association between acute idiopathic pulmonary hemorrhage and exposure to *Stachybotrys chartarum.* A systematic literature search of recent publications at NLM PubMed with the terms “pulmonary hemorrhage or pulmonary siderosis and dampness indoor or mold indoor or mold indoor” shows that no further publications on this topic appeared between 2008 and 2023. 

Reviews also find no clear causal links with mold exposure [58, 98, 279, 440, 542, 600, 601, 697]. 

In the online information for the public, the CDC continues to point out the lack of causality [93]. 

The evidence from the two reports mentioned above could form the basis for further systematic prospective studies on this issue. A case definition was provided by the CDC [89, 437]. 

For idiopathic hemosiderosis, no association with environmental factors was found in a cohort study; an autoimmunological genesis in connection with a genetic predisposition is suspected [374, 692]. 

At the present time, it is not justified to establish a causal relationship between pulmonary hemorrhages and the presence of indoor mold [369]. Nevertheless, a certain association cannot be excluded [127, 154]. In children with AIPH, it is recommended to ask if there is a history of moisture/mold [108, 437]. 

In summary, there is inadequate or insufficient evidence to date of an association between indoor moisture/mold exposure and pulmonary hemorrhage/AIPH (see Table 4 in chapter 2.3). 

**2.3.1.12 Infection susceptibility**

Many studies have shown consistent links between apparent indoor dampness or mold and effects on respiratory health or allergic diseases [182, 183, 449]. Fisk et al. [183] estimate that 8 – 20% of respiratory tract infections in the USA are associated with mold or indoor dampness. The correlation persists even after controlling for independent factors. 

*Penicillium* sp., *Cladosporium* sp., Zygomycetes and *Alternaria* sp. were found to be most closely associated with the occurrence of these diseases. 

The mechanism for this association appears to be of a non-allergic nature. 

In summary, it can be stated that there is sufficient evidence for an association between indoor moisture/mold exposure and susceptibility to infections (see Table 4 in chapter 2.3). 

Diagnostic methods are available to a limited extent (see chapter 3). 

Therapeutic procedures are available to a limited extent (see chapter 4). 

**2.3.1.13 Bronchitis**

Acute bronchitis is a clinical diagnosis characterized by cough due to acute inflammation of the large airways without signs of pneumonia [339]. 

Chronic bronchitis is caused by overproduction and hypersecretion of goblet cell mucus, leading to a progressive reduction in airflow due to luminal obstruction of small airways, epithelial remodeling and changes in airway surface tension that predispose to airway collapse. The incidence in adults is reported to be between 3.4 and 22.0%. Men are more frequently affected than women [336]. 

A quantitative meta-analysis found that indoor dampness and mold were associated with a substantial and statistically significant increase in bronchitis [182]. Subsequent studies also showed that indoor dampness/mold was associated with bronchitis (acute, chronic) [7, 316, 394, 467, 525, 532]. 

*Aspergillus *bronchitis is a discrete clinical entity in patients who are not significantly immunocompromised with structural lung disease such as bronchiectasis [104] or CF (mucoviscidosis) [61]. This type of bronchitis is distinct from asymptomatic fungal colonization and other forms of aspergillosis and may respond to antifungal therapy [104]. 

In summary, it can be stated that there is sufficient evidence of an association between indoor moisture/mold exposure and bronchitis (see Table 4 in chapter 2.3). 

Diagnostic methods are available (see chapter 3). 

Therapeutic procedures are available (see chapter 4). 

**2.3.1.14 Chronic obstructive pulmonary disease (COPD)**

Chronic obstructive pulmonary disease (COPD) describes a non-reversible restriction of airflow accompanied by a chronic inflammatory reaction of the bronchial system. Pathophysiologically, chronic inflammation leads to a loss of the ciliated epithelium and increased mucus production, which in turn leads to an increase in residual volume and a reduced FEV_1_ as an expression of obstruction [559]. The changes affect both the lung framework in the form of the alveolar walls and the pulmonary gas exchange surface (capillary system) and lead to an expansion of the distal air spaces resulting in pulmonary emphysema. 

COPD is one of the most common causes of death worldwide. Etiologically, exogenous factors such as all forms of inhaled smoke, recurrent bronchopulmonary infections, air pollution or fine dust pollution and (occupational) exposure to organic and inorganic dusts play a major role. Congenital and endogenous diseases (e.g., α-1-antitrypsin deficiency, Kartagener syndrome) play a minor role [559]. 

Molds seem to play a subordinate role in the pathogenesis of COPD and the cause of acute exacerbated COPD (aeCOPD), although a lack of systematic research into this association is criticized, a significantly higher rate of mold colonization has been found in patients with aeCOPD compared to the general population [210, 284, 287]. 

Exposure to mold in the home is associated with more frequent exacerbations and poorer outcome of COPD [197]. Preventive measures to avoid mold exposure are of particular importance here [123, 525]. The development of CPA on the basis of COPD occurs frequently [364]. Further studies on the influence of domestic mold exposure on the severity and development of COPD are required. 

The specific diagnosis and therapy will not be discussed here; please refer to the guidelines of the specialist societies [70, 747, 750]. 

In summary, there is limited or suspected evidence of an association between indoor moisture/mold exposure and COPD (see Table 4 in chapter 2.3). 

Diagnostic methods are available (see chapter 3). 

Therapeutic procedures are available (see chapter 4). 

Cross references:

- NVL program of BÄK, KBV, AWMF. S3 guideline National COPD care guidelines, version 2.0. Register number nvl - 003. Status: 25.06.2021. Valid until: 24.06.2026 (under revision); https://register.awmf.org/de/leitlinien/detail/nvl-003

**2.3.1.15 Asthma-COPD overlap syndrome (ACOS)**

Asthma-COPD overlap syndrome (ACOS) is a lung disease that has characteristics of both bronchial asthma and COPD. It is one of the so-called overlap syndromes [391, 530]. 

In addition to an environmental association (air pollution, ozone and particulate matter PM_2,5_), there appears to be a strong influence of smoking, particularly in patients who developed bronchial asthma or allergic rhinoconjunctivitis as adolescents and subsequently started smoking [391, 530]. 

In this respect, the etiology could be exposure to moisture damage with mold infestation and, as a result, inflammation of the airways [358] could favor the development or exacerbation of asthma or rhinoconjunctivitis and the development of ACOS, especially at a young age, if smoking is also present. A Finnish case-control study [303] showed that the risk of ACOS was significantly associated with mold odor in the workplace, but not with other humidity indicators. 

However, the risk of ACOS was not related to mold exposure at home, which does not rule out an association, as exposure at work is probably higher and longer, assuming that damage with a strong mold odor at home is probably eliminated more quickly. However, only 25 people fell into the “ACOS” category in this study, so the statement is based on a small number of cases. 

Since the pathophysiology of the syndrome has not been precisely clarified and no further epidemiological data are available, a possible link has not been proven but can be classified as a very plausible hypothesis. 

Critically, it should be noted that asthma-COPD overlap and “asthma and COPD” are only terms that are applied to patients with persistent airway obstruction, in conjunction with clinical manifestations that are compatible with both asthma and COPD. It is not a definition of a distinct disease entity, but only a description for clinical purposes, subsuming different clinical phenotypes that are subject to different mechanisms [217]. 

**2.3.1.16 Pneumonia**

In a study by Norbäck et al. (2018) [505], children who had various moisture/mold indicators (mold, water condensation on the window pane, smell of mold) at home at birth as well as children who were exposed to moisture and mold in their current living space showed an increased risk of pneumonia. In another study, various moisture/mold indicators (visible mold stains, damp patches, water damage, water condensation, damp clothing, damp bedding, mold odor) were associated with pneumonia in children, with the incidence of pneumonia increasing with the number of damp indicators [795]. 

Fungal infections of the lungs can occur in patients with a compromised immune system, the most common pathogens are *Aspergillus*, *Mucor*, *Fusarium,* and *Candida* spp. [218]. They also occur in critically ill patients with COVID-19, influenza, and community-acquired pneumonia [13]. According to a literature review by Lai and Yu (2021) [376], the most common pathogen in patients with COVID-19 is *Aspergillus fumigatus* followed by *Aspergillus flavus*. *Aspergillus fumigatus* is not only acquired in the hospital (nosocomial), but is presumably also brought in by the patients, most likely from the home environment [541]. 

*Aspergillus* pneumonia can also occur extremely rarely in immunocompetent people [665]. 

In summary, it can be stated that there is sufficient evidence for an association between indoor moisture/mold exposure and pneumonia (see Table 4 in chapter 2.3). 

Diagnostic methods are available (see chapter 3). 

Therapeutic procedures are available (see chapter 4). 

Cross references:

- German Society for Pneumology and Respiratory Medicine (DGP). S3 guideline Treatment of adult patients with community-acquired pneumonia, version 4.0. Register number 020 - 020. Status: April 24, 2021. Valid until: April 23, 2025; https://register.awmf.org/de/leitlinien/detail/020-020
- German Society for Pediatric Infectiology e.V. (DGPI) and Society for Pediatric Pneumology e.V. (GPP). S2k guideline Management of community-acquired pneumonia in children and adolescents (pCAP), version 1.0. Register number 048 - 013. Status: March 31, 2017. Valid until: March 30, 2022 (under revision); https://register.awmf.org/de/leitlinien/detail/048-013
- German Society for Pneumology and Respiratory Medicine (DGP). S3 guideline Epidemiology, diagnosis and therapy of adult patients with nosocomial pneumonia, version 2.0. Register number 020 - 013. Status: September 11, 2017. Valid until: September 10, 2022 (under revision); https://register.awmf.org/de/leitlinien/detail/020-013

**2.3.1.17 Irritative effects – mucous membrane irritation (MMI)**

In addition to various environmental factors, humidity [448], mold [120, 156, 659] and MVOC [359] are associated with mucosal irritation known as mucous membrane irritation (MMI)^2^ and chronic bronchitis [44]. The pathophysiological links between exposures to these environmental factors and MMI or chronic bronchitis are not yet clear, but the mucosal epithelium and local neurons are thought to play a key role in MMI [43]. According to a study from Denmark, long-term exposure to humid indoor environments appears to lead to mucosal hyperresponsiveness in the nasal provocation test with histamine, which persisted even after sanitization [589].

^2^Occasionally also referred to as mucous membrane irritation syndrome (MMIS). 

The frequency of mucous membrane irritation in people exposed to bioaerosols occupationally or environmentally is reported to be ~ 20 – 30% or more [261, 560, 570]. Reliable data on the frequency of these non-allergic, irritative, and inflammatory effects are not yet available in general and specifically for indoor exposure to mold. 

The possible irritative symptoms associated with MMI include non-specific irritation of the mucous membranes of the eyes (e.g., burning, tearing, itching), nose (sneezing, secretion, and obstruction of the nasal cavities), and throat (e.g., feeling of dryness, clearing of the throat). In addition, irritative inflammatory processes in the deeper airways (e.g., coughing) can manifest as chronic bronchitis [44]. Symptoms during exposure such as coughing, burning or itching of the eyes and nose as well as skin irritation quickly subside when exposure is interrupted. Allergic symptoms must be differentiated, which, unlike irritant reactions, usually increase with repeated and prolonged exposure due to sensitization [678]. The irritant-toxic effects of molds are possibly due to metabolic products and/or cell wall components (glucans) and as a reaction to the release of interleukins or other inflammatory mediators [596]. It is assumed that in addition to spores, hyphal/cell fragments in particular have a pro-inflammatory effect [273, 521]. Synergistic effects of different mycotoxins and/or of mycotoxins with other microbiological agents (e.g., glucans, endotoxins of bacteria) could be responsible for the effect [148, 149, 196, 270, 311, 358]. However, there is no obvious correlation between the total inflammatory potential (TIP) and the cytotoxic potential of a fungal species [413]. 

Patients with atopic diathesis will react more strongly to these irritant effects, as mast cells can be activated non-specifically by cell wall components and enzymes, e.g., via pseudoallergen receptors. The mucosal epithelium is damaged during the allergen-relevant period for atopic patients. This releases alarmins (TSLP, IL-33, IL-25), which induce a type 2 immune response. As a result, basophils and mast cells are activated and the inflammation continues to exacerbate. The disruption of the epithelial barrier can promote the development of inhalation allergies [731]. 

**Key message 6, section 1.3 Key messages:**

**Exposure to mold can generally lead to mucous membrane irritation (MMI), odor effects, and mood disorders.**

In summary, there is limited or presumed evidence to date of an association between indoor moisture/mold exposure and irritative effects/MMI (see Table 4 in chapter 2.3). 

There are no valid diagnostic methods available (see chapter 3). 

Therapeutic procedures are available to a limited extent (see chapter 4). 

**2.3.1.18 Sarcoidosis**

Sarcoidosis is a multi-organ disease with preferential involvement of the lungs. The main characteristic is granuloma formation, which is generally indicative of an infection or foreign body reaction [251]. 

It has been hypothesized that microbial cell wall components and bacterial lipopolysaccharides are the cause of sarcoidosis [431, 677, 693]. In the investigation of a sarcoidosis cluster in a building with water damage, it was suspected that thermophilic actinomycetes and non-tuberculous mycobacteria played a role [534]. 

Casuistically, the occurrence of bacterial or fungal infections in sarcoidosis has been repeatedly described; in some cases, DNA or proteins from microbial organisms have been found in the tissues of sarcoidosis patients [161, 441, 577, 649]. Several smaller and larger case series showed an increased prevalence of sarcoidosis in occupations that are sometimes associated with increased inhalation exposure to fire extinguishing emissions, metal and wet work or rooms with water damage [323, 368, 383, 554]. The central problem with these observations is that people with potentially damaging inhalation exposures are more frequently subjected to chest X-ray examinations compared to non-exposed persons, so that the probability of finding sarcoidosis as an incidental finding with no causal relationship to the inhalative noxious agent is increased solely due to the more frequent diagnosis. Finally, there are several intervention studies that demonstrate the efficacy of antibiotic treatment (overview in [431]) or antifungal [695] therapy. Adequate control groups are missing in both publications. The extent to which the apparently favorable findings are due to the concomitant immunosuppressive effects of antibiotics and not to their specific antimicrobial effect remains unclear. In addition to genetic factors and certain immunological prerequisites, a number of environmental factors also appear to play a role in the pathogenesis of sarcoidosis [81, 587, 593, 606]. 

In summary, there is only uncertain evidence that different inhalation microbial exposures, including moisture damage, can increase the risk of developing sarcoidosis; a causal association between mold exposure and sarcoidosis has not yet been established [689, 694, 696]. 

In future studies on the etiology of sarcoidosis, it would appear to make sense to ask about inhalative – including infectious – factors and moisture damage in the patient’s domestic and occupational environment [271, 383, 493, 494, 516, 532, 592]. It can also play a role in differential diagnosis [704]. At the current state of knowledge, however, there is insufficient data to causally link the development or exacerbation of sarcoidosis to moisture damage or molds. 

In the case of sarcoidosis, no other diagnostics related to molds are indicated beyond the usual procedure. 

In summary, there is limited or suspected evidence to date of an association between indoor moisture/mold exposure and sarcoidosis (see Table 4 in chapter 2.3). 

Valid diagnostic methods are available (see chapter 3). 

Therapeutic procedures are available to a limited extent (see chapter 4). 

**2.3.1.19 Rheumatoid arthritis, rheumatic complaints**

For years, infections (bacteria, viruses) have also been discussed as a trigger factor for many inflammatory rheumatic diseases. There are indications from a working group of an association between moisture damage and rheumatic complaints [410, 411, 417, 481, 490]. The occurrence of a cluster in a building was explained by the existing moisture damage and the “abnormal” microbiological exposure [417]. 

However, as long as no studies from other centers (and other countries) are available, it cannot be assumed that there is sufficient evidence. The epidemiological data situation is insufficient; therefore, no statements can be made on the occurrence and possible links between mold exposure and/or indoor humidity and rheumatic diseases. In a collagen-induced arthritis model, ochratoxin A (OTA) or deoxynivalenol (DON) increased the prevalence and clinical severity of rheumatoid arthritis compared to unexposed mice [305]. Whether these data can also be transferred to inhalation exposure is unclear. 

In case of rheumatic complaints, no other diagnostics related to molds are indicated beyond the usual rheumatological procedure (interdisciplinary guideline, management of early rheumatoid arthritis). 

In summary, there is inadequate or insufficient evidence to date of an association between indoor moisture/mold exposure and rheumatoid arthritis, rheumatic complaints (see Table 4 in chapter 2.3). 

Cross references:

- German Society for Rheumatology (DGRh). S3 guideline Management of early rheumatoid arthritis, version 3.0. Register number 060 - 002. Status: December 18, 2019; Valid until: December 17, 2024; https://register.awmf.org/de/leitlinien/detail/060-002

**2.3.1.20 Autoimmune diseases**

A study showed increased levels of antimitochondrial antibodies (AMA) in 6 patients exposed to indoor mold, mycotoxins, and water damage, which the authors interpret as possible mitochondrial damage and autoimmune triggering [397]. 

A review on the significance of mold/mycotoxin exposure in people with existing immune system dysfunction concludes that further research is needed to assess a possible link [365]. 

On this basis, an association between moisture/mold infestation in the interior and autoimmune diseases cannot be deduced. 

In summary, it can be stated that to date there is inadequate or insufficient evidence of an association between indoor moisture/mold exposure and autoimmune diseases (see Table 4 in chapter 2.3). 

**2.3.1.21 Mycotoxicoses**

Systemic effects (poisoning) caused by mycotoxins produced by molds are known as mycotoxicoses and are primarily known to occur when ingested orally via food [307, 475, 550]. 

Inhalation exposure to mycotoxins is possible both in the working and living environment [12, 199]. 

In the event of very high inhalation or dermal exposure, as may occur in the workplace without adequate protective measures [495], poisoning by mycotoxins has been described [434, 444]. 

However, there is as yet no reliable information on airborne intoxications caused by mycotoxins in indoor environments [92, 699]. There is a need for further clarification as to whether the mycotoxin concentrations produced in indoor air are systemically toxicologically relevant. According to the findings available to date, this does not generally appear to be the case [58, 95, 244, 523, 701]. In individual cases of extreme moisture damage with massive mold contamination and high exposure, symptoms of poisoning can presumably also occur indoors [47, 96, 203, 258, 321, 333, 369, 578]. 

Furthermore, it is unlikely that portal-of-entry effects, such as cytotoxic, inflammatory effects, on the respiratory tract can be causally attributed to mycotoxins alone, since exposure to damp and mold-contaminated indoor environments always involves complex exposure to other irritant, cytotoxic and immunogenic components of the bioaerosol [3, 15, 520, 687]. Hypothetical immunomodulatory effects of mycotoxins [727] require verification. 

Monitoring of inhaled mycotoxin exposure is currently not possible with sufficient certainty [244, 548]. 

In summary, it can be stated that there is inadequate or insufficient evidence to date of an association between indoor moisture/mold exposure mycotoxicoses (see Table 4 in chapter 2.3). 

**2.3.1.22 Odor effects**

The human nose is capable of smelling extremely low concentrations of substances stimulating our olfactory system. Users of damp buildings often complain of an inexplicable perception of odors, even to the point of odor nuisance, as shown by various studies based on questionnaire surveys [30, 298, 318, 446, 465]. 

Relevant odor perceptions may be caused by products of the primary and secondary metabolism of molds [599]. 

This should be a reason to investigate the causes of moisture/mold damage in the interior in terms of building physics [313]. The term MVOC is used to describe volatile organic compounds formed by molds and bacteria. The metabolism of molds and bacteria produces numerous volatile metabolites that are responsible for the “mold odor” as odor active volatile organic compounds (OVOC). Some substances have quite pleasant odor notes, for example 1-octen-3-ol has a typical mushroom smell, 2-heptanone a fruity smell and the terpene geosmin an earthy smell. Together, these substances produce a musty, typically “moldy” odor. This is perceived as unpleasant and is often associated with “spoilage” and “uncleanliness”. Sometimes those affected fear that the odorous substances themselves pose a toxic health risk. Many MVOCs have osmophoric groups (carbonyl, S, N, or OH groups) and have very low odor thresholds [418, 472, 551, 599]. 

It must be taken into account that for many so-called MVOCs there are other sources in addition to microbial ones (tobacco smoke, cooking, baking, roasting, potted plant soil, compost bins, etc.) [100, 621, 622, 623, 626]. 

It has not yet been clarified whether so-called MVOCs in concentrations in the lower µg/m^3^ range occurring indoors have biological signaling effects [226, 256]. Olfactory-psychological coupling reactions with unspecific complaints are possible with corresponding cacosmic abnormalities, but toxic reactions are unlikely [360, 361]. 

Odors in the environment can affect health and well-being in various ways. An odor nuisance comprises the following three components:

1. An emotional component (e.g., feeling of annoyance),
2. an interference component (e.g., obstruction of relaxation) and
3. a somatic component (e.g., nausea, vomiting, headache) [781].

A distinction must be made between direct physiological effects, the perception of an odor, odor nuisance as an effect of the odor on an emotional level, and indirect physiological effects as a result of odor nuisance and the associated chronic stress. In the reality of environmental medical assessment, the health effects of odors can often not be clearly separated from the aforementioned mechanisms. 

The characteristic effect of unpleasant odors is annoyance. As a health consequence, mood disorders are possible, but these are not mediated by toxicological mechanisms, but by conditioning, attribution (attribution of contexts) or stress [772]. Mood disorders can be seen as precursors of somatic dysfunctions. Typical symptoms of considerable unpleasant odor nuisance can be fatigue, lack of concentration, nausea, headaches, and insomnia [471]. 

Odor perception and cognitive evaluation, and thus also sensitivity to odors, exhibit large inter-individual differences. Genetic factors, age, gender and hormonal influences, imprinting, smoking behavior, context as well as adaptation, habituation and sensitization effects all play a role [122, 471]. 

**Key message 6, section 1.3 Key messages:**

**Exposure to mold can generally lead to mucous membrane irritation (MMI), odor effects, and mood disorders.**

In summary, it can be stated that to date there is limited or suspected evidence of an association between indoor moisture/mold exposure and odor effects (see Table 4 in chapter 2.3). 

Cross references:

- German Society for Ear, Nose and Throat Medicine, Head and Neck Surgery (DGHNO-KHC). S2k guideline Olfactory and tasting disorders, version 5.0. Register number 017 - 050. Status: May 01, 2023. Valid until: April 30, 2028; https://register.awmf.org/de/leitlinien/detail/017-050

**2.3.1.23 Environmental medical syndromes**

Syndromes are widespread in medicine. In addition to the syndrome, the disease includes the clear and unambiguous determination of the cause [237, 404]. A syndrome (Greek: σúγδϛoμoϛ = convergence) is a specific constellation of symptoms (or abnormalities) of a clinical picture,

- the cause(s) of which may be currently or generally unknown,
- which can have various causes,
- which cannot or cannot be reliably distinguished from other symptom constellations, or
- which are rather rare [237, 404].

Environmental medical syndromes include sick building syndrome (SBS) and multiple chemical sensitivity (syndrome) (MCS) as well as chronic fatigue syndrome (CFS) [779]. 

**Sick building syndrome (SBS)**

Numerous publications, mainly epidemiological studies, discuss a possible link between moisture/mold exposure and SBS (used synonymously: building-related (health) symptoms; not correctly used by definition as a synonym: building-related illness^3^) is discussed [6, 9, 17, 32, 51, 105, 110, 155, 167, 200, 228, 246, 266, 268, 279, 312, 315, 333, 334, 335, 340, 387, 393, 403, 412, 416, 420, 443, 458, 459, 483, 484, 496, 506, 510, 524, 531, 567, 572, 597, 602, 603, 604, 605, 607, 617, 620, 631, 634, 650, 651, 660, 679, 681, 688, 698, 715, 719, 780], even with a pre-SBS [485].

^3^Definition of building-related illness (BRI): Clinically clearly defined clinical pictures (including humidifier fever, legionellosis, indoor-associated allergies e.g., to house dust mites or molds, indoor-associated malignancies such as radon-associated lung carcinoma) [429, 633] for which the etiology, pathology, pathophysiology, diagnosis, therapy, prevention, and prognosis are clearly known. 

However, a variety of physical, chemical, biological, psychosocial and personal factors have been discussed as possible causes of SBS, without a clear etiology having been determined to date. It is therefore assumed to be a multifactorial process in which the simultaneous occurrence of different influences and thus variable combined effects leads to the development of the syndrome [52, 53]. 

**Multiple chemical sensitivity (syndrome) (MCS)/idiopathic environmental intolerance (IEI)**

Some studies discuss a possible link between indoor moisture/mold infestation and MCS [289, 290, 333, 389, 432, 506, 683, 717, 720, 752, 753, 792, 793]. Dampness and mold hypersensitivity syndrome is also occasionally reported [720, 728] or toxic mold syndrome [333, 392]; the latter not without contradiction [96]. 

However, MCS is an impressive example of the complex, often very individual and subjective interactions between body, psyche and environment [247, 248]. Despite the absence or low level of somatic findings, patients often suffer so much that it is almost impossible to cope with everyday life. This results in social and financial losses as well as high direct and indirect healthcare costs. The long-standing dualistic debate as to whether MCS is “physical” or “psychological” has unsettled many sufferers, wasted time and resources searching for causes and trying to find a cure, but has not brought about any satisfactory improvement in the situation of MCS patients. They often feel turned away by “conventional medicine”, and doctor-patient relationships are regularly experienced as difficult. Those affected therefore often turn to alternative medical, scientifically unvalidated explanatory models and treatment methods, which may provide subjective relief, but rarely improve symptoms and participation and may be associated with dangerous side effects and high costs. From a scientific point of view, MCS is not yet a clearly definable clinical picture, but probably a special manifestation of a functional disease that is particularly stressful in individual cases [247, 248]. In particular, the lack of a causal relationship between exposure and symptoms, the chronic course and the comorbidities point to a general hypersensitivity that is not necessarily substance-related. However, their mechanisms of action, structural and functional correlates require further scientific substantiation, also with regard to their therapeutic modifiability. An understanding of MCS as a dysfunctional vicious circle of negative experiences and evaluations, psychophysiological tension and hyperreactivity offers both the patients themselves and their treating physicians a comprehensible psychoneurobehavioral model. It also implies at least potential reversibility and opens up concrete options for action, such as reviewing and relativizing threat expectations, focusing attention and avoidance behavior [247, 248]. 

**Chronic fatigue syndrome (CFS)**

In contrast to the previously discussed syndromes, only a few studies address a possible link between indoor dampness/mold exposure and CFS [24, 214, 662]. 

Here, too, is assumed that CFS etiologically has multifactorial genesis with biological, social and psychological factors [375]. In addition, the Committee on the Diagnostic Criteria for Myalgic Encephalomyelitis/Chronic Fatigue Syndrome, Board on the Health of Select Populations, Institute of Medicine has issued a 304-page statement on the subject of myalgic encephalomyelitis/CFS [109], in which terms such as mold, mycotoxins, or MVOCs are not mentioned. 

To date, there is insufficient evidence of a causal link between the environmental medical syndromes SBS, MCS, and CFS and indoor exposure to moisture/mold [286]. 

In summary, there is sufficient evidence for an association between indoor dampness/mold exposure and disease patterns that can be correctly classified as BRI (building-related illness) by definition, if there is a clear exposure-related association [366]. This conditionally includes the diseases listed in Table 4 in chapter 2.3 under “Sufficient evidence for an association”. 

However, there is inadequate or insufficient evidence for a link between indoor moisture/mold exposure and the environmental medical syndromes SBS, MCS, and CFS (see Table 4 in chapter 2.3). 

Newly coined terms such as biotoxicosis and mold and dampness hypersensitivity syndrome (MDHS) [719, 728] or volatoxins [47], suggest a nosological specificity of a pathophysiological connection for which there is no evidence to date. 

**2.3.1.24 Sensitivity disorders, non-specific symptoms**

Disorders of well-being are understood to be *“deteriorations in psychological, physical and social well-being as well as the feeling of subjective performance. As an emotional experience, they are to be distinguished from annoyance reactions that involve a cognitive evaluation of specific environmental stimuli“* [67, 68]. Disturbances of well-being play a significant role in environment-associated health disorders in general and in interior-associated health disorders in particular [772]. The following three models are used to explain the mechanisms of action of such environment-associated mood disorders [67, 68]:

- Model of the noxious agent: Physiological relationship between an environmental factor and a person’s reaction to, for example, a psychotropic substance.
- Model of attribution: A health condition is attributed to an environmental factor according to a cognitive assessment process.
- Stress model: An environmental factor is consciously perceived and experienced as unpleasant, harmful, or threatening. Stress reactions can manifest themselves as physical dysfunctions, changes in well-being and impaired performance.

Changes in well-being can include anxiety, depression, impaired concentration and memory, psychophysiological activation reactions of blood pressure and hormone concentrations as well as vegetative complaints such as headaches and exhaustion. 

In principle, it is possible for moisture damage and mold to trigger environmentally associated disorders of well-being, for example visual, cognitive and/or odor-related perception of a possible mold infestation (see chapter 2.3.1.22) [772]. 

**Key message 6, section 1.3 Key messages:**

**Exposure to mold can generally lead to mucous membrane irritation (MMI), odor effects, and mood disorders.**

In summary, it can be stated that there is limited or suspected evidence to date of an association between indoor moisture/mold exposure and sensitivity disorders, non-specific symptoms (see Table 4 in chapter 2.3). 

Cross references

- German Society for Psychosomatic Medicine and Medical Psychotherapy e.V. (DGPM) and German College of Psychosomatic Medicine e.V. (DKPM). S3 guideline Functional body complaints, version 2.0. Register number 051 - 001. Status: July 18, 2018. Valid until: July 17, 2023 (under revision); https://register.awmf.org/de/leitlinien/detail/051-001

**2.3.1.25 Neuropsychological, neurotoxic effects**

While neurotoxic effects have been documented for microbial toxins of aquatic origin (dinoflagellates, cyanobacteria) with inhalation exposure [414, 457, 748], there are no systematic or epidemiological studies with inhalation exposure to neurotoxic mold toxins. 

Exposure to toxin-producing molds (“toxic mold”) in indoor environments has been linked to neurotoxic effects and cognitive and emotional problems have been attributed to mycotoxins (“black toxic mold syndrome”) [23, 24, 25, 27, 38, 117, 222, 223, 289, 564, 656] and toxin-discharging therapies propagated. These studies must be criticized due to methodological weaknesses [223, 224, 438]. Recent epidemiological studies found associations between exposure to moisture and mold and sleep disorders and hyperactivity (ADHD) in children [82, 84, 85, 705, 708]. Also for depression [647], cognitive development [221, 308] such a correlation is reported. A causal attribution to mycotoxins is uncertain, as growing up under poor housing and living conditions as a stressor in itself could explain these effects [407, 460, 461, 498]. 

Connections with neurodevelopmental disorders such as autism [564] and Alzheimer’s [65, 153] are considered speculative. No consistent connection can be derived from the specialist literature that neurotoxic effects are caused by the toxin concentrations occurring indoors [78, 96, 333, 390, 698]. The evidence for an association can be classified as insufficient [223], but an association cannot be excluded [16]. 

In summary, it can be stated that so far there is inadequate or insufficient evidence for an association between indoor moisture/mold exposure and neuropsychological, neurotoxic effects (see Table 4 in chapter 2.3). 

**2.3.1.26 Gastrointestinal diseases, renal diseases, thyroid diseases, endocrinopathies, reproductive disorders, teratogenicity, sudden infant death syndrome, cancer**

Occasionally, (non-scientific) publications and internet forums postulate hypotheses about connections between molds and numerous other different clinical pictures [26, 662, 717, 718], with the result of unsettled patients who have “informed themselves” on the internet. Molds are also sometimes wrongly equated with “intestinal fungi” (commensal colonization with *Candida albicans*). 

To date, there are no systematic studies or case reports that prove causal link between moisture damage or mold indoors and gastrointestinal or renal diseases, thyroid diseases, endocrinopathies, reproductive disorders, teratogenicity, sudden infant death syndrome or cancer (see [257, 437, 526]). Exceptions are rare invasive gastrointestinal fungal infections in immunocompromised patients [557]. 

A association between sudden infant death syndrome (SIDS) and tobacco smoke as well as moisture damage with mold exposure was already speculated 20 years ago in connection with the deaths of the so-called “Cleveland babies” (see above chapter 2.3.1.11) [125, 171]. Since then, however, no more recent studies have been published on the subject of mold exposure and SIDS that prove a causal relationship with mycotoxin exposure. Three risk factors appear to predispose to SIDS: an exogenous stressor (e.g., sleeping in the prone position, exposure to smoke, air pollutants such as CO and NO_x_), a critical developmental period (usually age 2 – 4 months) and an underlying susceptibility, such as a genetic disposition [216, 405]. Until today, it is not possible to predict infants at risk of SIDS. 

Endocrinopathies are also thought to be linked to various environmental factors [402, 575]. Environmental factors are plausible as a cause of some diseases, for example for food-related – usually very high – mycotoxin exposure (e.g., [402, 661, 789]). Occasionally, publications (e.g., [134]) also speculate about exposure to moisture damage with mold infestation, such as suspected damage to the pituitary gland with growth disorders, thyroid disease and diabetes caused by mold toxins or antibodies. There is currently insufficient scientific evidence to support the hypothesis that exposure to molds and mycotoxins (“toxic mold”) is causally involved in the development of systemic diseases such as Parkinson’s disease, amyotrophic lateral sclerosis (ALS), diabetes mellitus and endocrinopathies (e.g., thyroid diseases, hypopituitarism) [507], as most studies lacked power to detect exposure/disease associations and had methodological weaknesses, for example, were not prospective or long-term, did not start in infancy, had inaccurate or no exposure estimates, had unclear exposures and did not consider genetic susceptibility. 

In all these cases, it is the doctor’s task to provide objectification. 

**Key message 8, section 1.3 Key messages:**

**Physicians shall, in cases of a suspected association between indoor moisture/mold damage and conditions for which there is no evidence of such an association (e.g., acute idiopathic pulmonary hemorrhage in children, arthritis, autoimmune diseases, chronic fatigue syndrome (CFS), endocrinopathies, gastrointestinal effects, cancers, airborne mycotoxicoses, multiple chemical sensitivity (MCS), multiple sclerosis, neuropsychological effects, neurotoxic effects, sudden infant death syndrome, renal effects, reproductive disorders, rheumatism, thyroid disorders, sick building syndrome (SBS), teratogenicity, and urticaria), inform affected individuals objectively about the current state of knowledge.**

In summary, there is inadequate or insufficient evidence to date of an association between indoor moisture/mold exposure and gastrointestinal or renal diseases, thyroid diseases, endocrinopathies, reproductive disorders, teratogenicity, SIDS, cancer (see Table 4 in chapter 2.3). 

Cross references

German Society for Sleep Research and Sleep Medicine e.V. (DGSM). S1 Guideline Prevention of Sudden Infant Death Syndrome, version 3.0. Register number 063 - 002. Status: November 06 2022. Valid until: November 05, 2027 (under revision); https://register.awmf.org/de/leitlinien/detail/063-002

### 2.4 Risk analysis and assessment 

**2.4.1 Risk of infection**

The risk of infection from the mold species regularly found indoors is low for healthy people, most species are classified in risk group 1 and a few in 2 (*Aspergillus fumigatus*, *A. flavus*) of the Biological Agents Ordinance [712]. 

For occupational activities (handling) involving molds, the current Biological Agents Ordinance applies, according to which the infection risks of biological agents are divided into four risk groups [743], whereby the molds are distributed between risk groups 1 and 2: 

Risk group 1: Biological agents that are unlikely to cause disease in humans. 

Risk group 2: Biological agents that may cause disease in humans and may pose a risk to workers; the substance is unlikely to spread to the population; effective prevention or treatment is usually possible. 

Risk group 3: Biological agents that can cause serious disease in humans and pose a serious risk to workers; there may be a risk of spread to the population, but effective prevention or treatment is usually possible. 

Risk group 4: Biological agents that cause serious disease in humans and pose a serious risk to workers; the risk of spread to the population may be high; effective prevention or treatment is not normally possible (risk group 4 does not include fungi). 

Mold mycoses are opportunistic infections. They require a reduced immune system in exposed persons. Thermotolerant mold species of risk group 2 (e.g., *Aspergillus fumigatus, A. terreus, A. niger, A. flavus, Emericella nidulans,* or mesophilic *Fusarium *sp.) of “TRBA 460: Classification of molds into risk groups” [712] of the Biological Agents Ordinance [743] rarely cause infections in healthy, immunocompetent people, but can trigger invasive mycoses in people whose immune system is incompetent due to illness or other circumstances [401]. 

The WHO comes to a similar conclusion in its current WHO fungal priority pathogens list for guide research, development, and public health action [784]. 

According to the recommendation of the KRINKO at the RKI [349], immunocompromised persons can be divided into three risk groups (Table 9). 

Particularly at risk are (listed with decreasing risk) patients with tumor disease, especially with underlying hemato-oncological disease (e.g., leukemia, lymphoma), acute myeloid leukemia (AML), acute lymphoblastic leukemia (ALL), allogeneic stem cell transplantation, autologous stem cell transplantation, solid organ transplantation, HIV infection, other immunosuppression (e.g., protracted high-dose glucocorticoid therapy), aplastic anemia, CF, etc. [254, 482, 555, 581, 582, 612]. Acute myeloid leukemia (AML) is associated with the highest incidence of invasive mold infections (~ 12%) and most mold infections (~ 8%). This is followed by acute lymphoblastic leukemia (~ 4%). Among the procedures, allogeneic hematopoietic stem cell transplantation is associated with a very high incidence of mold infections [254]. 

In addition, critical patients in intensive care units are at risk of *Aspergillus* infection [102, 309, 346, 372]. 

Patients with influenza [8, 114, 281, 406, 427, 648] and patients with COVID-19 [8, 37, 174, 541, 588, 613, 615, 733, 749] have an increased risk of contracting fungal infections. 

The same applies to other severe respiratory viral infections [419, 549]. 

Due to the steady increase in the proportion of immunocompromised people in the population and the longer survival, it cannot be ruled out at present that mold infections may become an increasing risk factor for the health of this population group [349]. 

A numerical risk cannot be derived on the basis of the current state of knowledge. Risk matrix 1 shows a semi-quantitative risk assessment of the risk of infection from indoor molds. 

Cross references:

- Society for Pediatric Oncology and Hematology (GPOH). S1 guideline Acute myeloid leukemia - AML - in childhood and adolescence, version 2.0. Register number 025 - 031. Status: March 25, 2019. Valid until: March 24, 2024; https://register.awmf.org/de/leitlinien/detail/025-031
- Society for Pediatric Oncology and Hematology (GPOH). S1 guideline Acute lymphoblastic leukemia - ALL - in children, version 7.0. Register number 025 - 014. Status: May 31, 2021. Valid until: May 30, 2026; https://register.awmf.org/de/leitlinien/detail/025-014
- German Society for Hematology and Medical Oncology (DGHO). S3 guideline Diagnostics, therapy and follow-up for patients with chronic lymphocytic leukemia (CLL), version 1.0. Register number 018 - 032OL. Status: March 31, 2018. Valid until: March 30, 2023 (under revision); https://register.awmf.org/de/leitlinien/detail/018-032OL
- German Society for Internal Intensive Care and Emergency Medicine e.V. (DGIIN), German Interdisciplinary Association for Intensive Care and Emergency Medicine e.V. (DIVI), German Society for Pneumology and Respiratory Medicine e.V. (DGP), German Society for Infectiology e.V. (DGI). S3 guideline Recommendations for the treatment of patients with COVID-19 - Living Guideline, version 8.1. Register number 113 - 001LG. Status: September 12, 2022. Valid until: September 11, 2023; https://register.awmf.org/de/leitlinien/detail/113-001LG
- Society for Pediatric Pneumology e.V. (GPP), German Society for Pediatric and Adolescent Medicine e.V. (DGKJ). S3 guideline Pulmonary disease in cystic fibrosis: *Pseudomonas aeruginosa*, version 2.0. Register number 026 - 022. Status: September 27, 2022. Valid until: September 26, 2027: Guideline manuscript submitted for review, revision not yet completed; https://register.awmf.org/de/leitlinien/detail/026-022

**2.4.2 Risk of sensitization / allergy**

In principle, there is also the possibility of sensitization and the triggering of a clinically symptomatic allergy in healthy people after inhalation of spores and other mold components (e.g., mycelium). The sensitizing potential of molds compared to other environmental allergens, such as allergens from fur-bearing pets, grass and tree pollen or house dust mite (~ 15 – 30% [206, 796]), is estimated to be significantly lower [292, 490, 690, 691]. Both population-based and patient-based studies show a comparatively low prevalence of sensitization across Europe of 3 – 22.5% [193, 241, 255], which varies greatly depending on the type of mold and shows a north-south gradient (low prevalence of sensitization in Finland, relatively high in Greece) [255]. 

**Key message 11, section 1.3 Key messages:**

**In principle, a large number of mold species can cause sensitization and allergies in case of corresponding exposure. Compared to other environmental allergens, however, the allergenic potential is to be regarded as lower overall [** 241 **,** 255 **].**

It is generally assumed that there are over one million mold species. To date, ~ 350 mold species have been listed as potentially sensitizing at www.allergome.org. However, it cannot be concluded from this information how high the overall proportion of sensitizing mold species is. The WHO/IUIS criteria for classifying an allergen are currently met by 107 mold proteins from 43 mold species (www.allergen.org). Only a few molds are available as test allergen solutions and typical indoor fungal allergen extracts are largely absent [327, 330]. 

From an allergological point of view, following sensitization of a patient to mold fungi, a dose-dependent exposure (measured as CFU) is not the only decisive factor for the clinical reaction. Sensitization with the formation of specific IgE antibodies and the triggering of allergic reactions occurs at the level of proteins or peptide components. It is therefore not necessary for whole spores or intact mold mycelium to be present. Rather, the allergenicity depends on the proteins or peptides and also on the susceptibility of the exposed person, so that an antigen becomes an allergen and triggers sensitization or, in the case of repeated contact, an allergy. An antigen only becomes an allergen when the antigen reacts with a person’s immune system, which responds with an IgE reaction.^4^

^4^Definition of the term allergen: Allergens are antigens which cause allergy. Most allergens reacting with IgE and IgG antibody are proteins, often with carbohydrate side chains, but in certain circumstances pure carbohydrates have been postulated also to be allergens. In rare instances low molecular weight chemicals, e.g., isocyanates and anhydrides acting as haptens, are still referred to as allergens for IgE antibodies. In the case of allergic contact dermatitis, the classical allergens are low molecular weight chemicals, e.g., chromium, nickel and formaldehyde, reacting with T cells. (Nomenclature by WAO/EAACI (World Allergy Organization/European Academy of Allergy and Clinical Immunotherapy), http://tmedihk.com/allergy-basics/).

In people with atopy, rhinoconjunctivitis, and rhinosinusitis, exposure to damp indoor spaces is a risk factor for the development of bronchial asthma. Rhinosinusitis associated with mold exposure doubles the risk of developing bronchial asthma (OR: 2.2; CI: 1.3 – 3.6) [533]. Infants with atopy appear to have an increased risk of developing bronchial asthma if there is moisture damage or mold in the bedroom or living room [318]. 

A numerical risk cannot be derived on the basis of the current state of knowledge. Risk matrix 2 shows a semi-quantitative risk assessment of the sensitization/allergy risk from indoor molds. 

**2.4.3 Risk of irritant effects**

Mucous membrane irritations of the eyes and upper respiratory tract have been described in various publications (see chapter 2.3.1.17). The same applies to chronic bronchitis (see chapter 2.3.1.13). 

So far, it is unclear whether people affected by MMI or chronic bronchitis are particularly sensitive individuals who react at lower doses, or sensitized individuals who react differently than non-sensitized individuals regardless of the dose [43]. 

Possible predisposing factors for MMI and chronic bronchitis can be other inflammatory processes in the area of the mucous membranes of the eyes and respiratory tract, such as infections, atopic mucosal diseases, keratoconjunctivitis sicca and dry nasal mucous membranes [354]. 

**2.4.4 Risk of toxic effects**

Only molds that are potentially capable of producing toxins can be considered as triggers of toxic effects. Whether toxin formation occurs indoors in individual cases is determined by the environmental and growth conditions and, above all, the substrate [437, 523]. 

There are no known predisposing factors for toxic reactions caused by mycotoxins in humans. However, predispositions are conceivable at the organ level. For example, it is conceivable that a previously damaged liver (e.g., chronic hepatitis, liver cirrhosis) may be a predisposition to hepatotoxic aflatoxin effects following oral ingestion of this toxin. Whether this also applies to aerogenic toxin uptake is still unclear [321, 354]. 

A numerical risk cannot be derived on the basis of the current state of knowledge [78]. 

**2.4.5 Risk of odor effects and mood disorders**

In principle, anyone can be affected by odor effects and/or discomfort in the event of moisture/mold damage indoors. This is a nuisance, not a health hazard. 

Predisposing factors for odor effects can be genetic factors, age, gender and hormonal influences, imprinting, smoking, context as well as adaptation, habituation and sensitization effects [122, 471]. 

Predisposing factors for mood disorders can be environmental concerns, fears, conditioning and attributions as well as a variety of illnesses [774]. 

## 3 Diagnostics 

### 3.1 Reason for consultation 

Patients usually seek medical advice in connection with mold exposure for the following reasons [475, 678]:

- Patients who have health complaints and whose circumstances suggest an environmental connection with moisture damage and/or mold exposure.
- Patients with mood disorders and non-specific symptoms that have a clear temporal connection with certain environmental conditions or activities.
- Patients concerned about possible mold exposure.
- Measurement results are already available.
- Medical assistance is sought in rental and construction disputes.

### 3.2 Diagnostics – general procedure, medical history, physical examination, clinical-chemical and instrumental tests 

The basic elements of every medical diagnosis are the medical history [236] and the physical examination. Building on this, further special examination methods are carried out by the relevant medical disciplines depending on the problem and the differential diagnosis. In environmental and occupational medicine, where possible and indicated, examinations of internal exposure (human biomonitoring as exposure and/or effect monitoring) and/or external exposure (home visit/site visit, environmental monitoring) are also carried out. 

**3.2.1 Medical history**

When taking the medical history, a holistic approach should be taken that is not limited to environmental pollutions and aspects of physical illness, but also includes the psycho-social dimension of the problems. This approach, which is particularly necessary in view of the high expectations patients have, should be explained to the patient. The equal inclusion of the psychological and social aspects hardly encounters any difficulties in the consultation if this is explained to the patient accordingly. 

If there is a suspicion of possible health problems caused by molds, the following elements of the medical history should be considered in addition to the general and differential diagnostic history:

- Exposure history in the living area
- Exposure history in the work area
- Exposure history in the leisure sector
- Infectious disease history incl. predisposition factors
- Allergological history incl. predisposition factors
- History of irritant-toxic effects
- Medical history regarding odor effects
- Medical history regarding mood disorders

Some aspects are discussed in more detail below. Where no literature is provided, only experience from daily practice is available. 

**3.2.1.1 Allergy history**

History-taking for suspected mold-associated diseases consists of the general patient history as well as their special environmental medical and allergy history. 

The patient’s own medical history should be taken, taking into account the family history (atopy). Experienced allergists usually obtain up to 50% agreement with the allergy tests from the history of temporal and spatial exposure to allergens [653, 658]. The specific environmental medical allergy history in the case of mold growth is particularly helpful to initiate a targeted diagnosis and to detect competing allergens. 

A mold allergy can only be diagnosed with certainty when the medical history, clinical symptoms, allergen detection, and, if necessary, provocation tests are taken together. 

Allergy history consists of two sets of questions. The underlying diseases, symptoms, and complaints are asked in relation to specific organs. 

The medical history is supplemented in the second part by the possible mold allergens, competing allergens, and the temporal and spatial associations of complaints with allergen exposure. 

In addition, non-allergic symptom complexes are identified and differentiated. These include mood disorders, neuropsychological symptoms, odor effects, toxic-irritative effects of molds, their components, and bacteria. 

**First step of the medical history – organ-related symptoms**

In the first step of the medical history, organ-related symptoms and their onset, intensity, and duration are asked. There are no specific symptoms in patient history that allow a reliable conclusion to be drawn about the cause of an allergy to molds. The allergic symptoms caused by immediate-type sensitization are independent of the specific allergen causing them. The severity of the allergic symptoms is triggered by a variety of endogenous and exogenous factors (e.g., tobacco smoking). Stress can exacerbate allergic symptoms. This includes emotional conflicts in the partnership, the family, or at work. Exposure to stress, for example after water damage and mold infestation indoors, can also exacerbate allergic symptoms. In women, but also in men, the allergic reaction threshold and organ localization can change depending on the hormone balance. 

**Second step of the medical history – allergen exposure**

In the second step of the medical history, the location, time, and allergen reference of the symptoms should be determined. For this purpose, patients should be asked about the onset, course, and severity of symptoms at different locations. In accordance with the ecology of mold fungi, symptoms should also be investigated in relation to the weather, season, location at home, e.g., in air-conditioned rooms or in the bedroom (note: mold fungi occur together with mites in the bedroom). The focus is on questions about a “mold smell”, visible mold growth or mold contamination indoors. The smell of mold growth is often described as, musty to sour. Moisture damage, thermal bridges, condensation, waste disposal, the handling of organic waste garbage cans and garbage, compost storage, keeping animals, the presence of indoor plants, hobbies, user behavior and structural defects should be investigated. The living conditions and user behavior can also be indicative. The heating and ventilation options of the building or apartment, the year of construction, the roof construction, the attic, and the cellar rooms should be taken into account. Demolition, construction, and renovation work in the building and its surroundings can also contribute to mold allergen exposure [40, 76, 166, 301, 409, 702]. Woodlice (lat. *Oniscidea*), silverfish (lat. *Lepisma saccharina*), and especially dust lice (lat. *Psocoptera*), which feed on “mold turf”, can be taken as an indication of increased moisture in building components. 

Also relevant is the transfer of mold fungi and their components through the stirring up of decaying biological material (leaves, grass, soil, wood). This can be caused by motor blowers during cleaning, gardening, and landscaping work, as well as by strong winds in a dry period following a wet weather period. Important, often not easily recognizable mold reservoirs are ventilation and air conditioning systems that are not regularly maintained. Questionnaires can be used to explore the exposure anamnesis. These are used in routine clinical practice, e.g., in cases of EAA [544], or in studies dealing with the topic of mold exposure [331, 399]. 

It is of central importance whether typical complaints of possible health effects of mold fungi occur in a spatial and temporal relation to rooms or activities. As part of the diagnostic procedure, a period of non-exposure, i.e., temporarily leaving the suspected rooms and then re-exposure, can also provide important information [381]. 

To determine the local and temporal dependencies of the complaints, it can be useful for the patient to keep a symptom diary. 

**3.2.1.2 Susceptibility history**

The medical history is intended to identify particularly vulnerable and sensitive individuals such as immunosuppressed persons, allergic persons (atopics), and persons with underlying pulmonary diseases such as asthma, COPD, and CF (mucoviscidosis) [127, 146, 482]. Patients with CF have an increased risk of ABPA. 

Predisposing factors for a mold allergy are a familial history of type I allergies, existing sensitization, and the presence of one or more atopic diseases. The significance of these predispositions increases in the order shown. In the context of sensitization and atopic disease, the more mold-specific the predisposition, the more pronounced it is [354]. 

Other predisposing factors are:

- Allergic rhinitis that is difficult to treat,
- sinusitis that is difficult to treat,
- asthma that is difficult to treat,
- exacerbating asthma for unclear reasons.

**3.2.1.3 Occupational mold exposure**

Occupational exposure to molds varies qualitatively and quantitatively depending on the activity. According to the 2013 amendment to the Biological Agents Ordinance, which represents the national implementation of Directive 2000/54/EC, the classification of the risk of infection no longer applies to activities where a sensitizing effect is primarily to be assumed. This applies to occupational mold exposure. In principle, occupational mold exposure is to be regarded as given in the following areas of activity [713]:

- Activities in the waste sector (waste management, composting, waste separation, waste incineration, sorting of recyclable materials)
- Commercial mold sampling and handling of sample material from areas contaminated with mold
- Remediation of mold-infested interiors
- Staying in archives, libraries, depots, and warehouses (old paper or mold infestation)
- Activity in agriculture (hay, litter, animal husbandry)
- Gardeners, landscape gardeners, florists, tree work
- Millers and bakers
- Winegrowers (especially for destemming grapes)
- Food industry (refinement of dairy and meat products)
- Breweries
- Paper and wood production and processing
- Handling cooling lubricants (aerosol with bacteria and molds)
- Building renovators (upholsterers, plumbers)
- Feed production and handling of feed
- Ventilation/air conditioning maintenance

If employees have workplace-associated symptoms when handling materials containing mold fungi that indicate an allergy to mold fungi, the exposure (frequency, quantity, quality) must first be examined as part of a risk assessment. The TRBA, the protective measures based on the risk of infection (TRBA 460) or the specific processing (TRBA 212 or TRBA 214) must be observed. The basic rule for safety and health protection is to initially avoid or at least minimize hazards through substitution and technical measures. If the technical measures are not sufficient to exclude a health hazard, organizational and personal protective measures must be added. This hierarchy of protective measures is referred to in occupational medicine as STOP (substitution, technical, organizational, personal measures). Technical measures must be taken if necessary. Personal protective equipment must be tested. Employers and doctors are obliged to report the suspicion of an occupational disease to the responsible accident insurance institution (§§ 193, 202 SGB (Social Security Code) VII). 

### 3.3 Physical examination 

Every medical history includes a complete or at least symptom-oriented physical examination. The methodology of the physical examination is based on inspection, palpation, percussion, auscultation, and functional testing. 

This means that the target organs identified in the medical history should be examined first. Particular attention should be paid to the mucous membranes of the eyes and, if possible, the upper respiratory tract and the skin, since non-specific complaints that patients often complain about primarily affect these organs [408]. In principle, the physical examination should be carried out in a structured and standardized manner and adequately documented. Various clinical findings forms are available for this purpose. 

### 3.4 Markers for mold exposure 

Studies on mold exposure are carried out for a wide variety of purposes, with some of these studies being targeted and others postulating that they can prove a causal relationship between mold exposure and possible health effects. However, the latter studies, some of which are very costly in terms of time and money, often do not meet the expectations placed on them. In addition, a distinction must be made between studies that are used in individual medical examinations and those that are only realistically applicable for scientific purposes. 

A basic distinction must be made between investigations and parameters that relate to external exposure, i.e., contact outside the body (environmental monitoring), and investigations in body media (biomonitoring). Biomonitoring, in turn, is subdivided into biomonitoring related to internal exposure (biomonitoring of exposure), biomonitoring related to effects of exposure (biomonitoring of effect), and biomonitoring related to susceptibility (biomonitoring of susceptibility). Environmental monitoring is discussed in more detail below. 

Biomonitoring of exposure, effect and susceptibility are part of medical-clinical diagnostics and are described in the corresponding chapter 3.5. 

**3.4.1 Environmental monitoring**

As a rule, there is no medical indication for the determination of molds indoors or in building materials or on furnishings. 

**Key message 3, section 1.3 Key messages**

**For medical indication, indoor mold measurements are rarely useful. As a rule, both a quantitative and a qualitative determination of mold species can be dispensed with in the case of visible mold infestation. Rather, the causes of the infestation should be clarified, and then the infestation and primary causes should be eliminated.**

Epidemiological studies show that there is an association between moisture/mold damage in indoor spaces and health complaints among users. The health conditions observed are obviously related to the microorganisms that have grown on and possibly in damp material. However, it has not yet been possible to clarify which specific agents are particularly relevant in this context. In addition to molds, bacteria, especially actinobacteria, also grow in damp matrices. The organism itself, but also metabolic products and cell components of the microorganisms, such as toxins, allergens, MVOCs, β-glucans, endotoxins and fragments of molds (particles) or bacteria can have a health effect. Mites and amoebae often occur concomitantly in the case of moisture/mold damage so that an increased health effect of these parasites and microorganisms can also be expected. There is no simple causal relationship between one of the above-mentioned noxious factors and the health effects that occur [135, 159]. With appropriate exposure, both cultivable and non-cultivable mold spores can have a health effect. The same applies to mycelium fragments. This means, for example, that allergenic components of molds can still be detected even after disinfection measures [347]. 

The absorption pathways of the various noxious substances in the possible media also take place in different ways. For example, airborne MVOCs and mold spores, mycelial fragments of molds as well as bacteria, toxins, endotoxins, and other allergens such as pollen are inhaled. However, many mold allergens can also be ingested orally via food or percutaneously via contact, e.g., with infested building materials [57]. 

Even the most comprehensive examinations to identify and quantify mold fungi and the other indoor noxious substances that occur in the event of moisture/mold damage, they are of little use to the attending physician in confirming the diagnosis and therapy, since there is no simple causal association between the individual health complaints that have occurred and the mold infestation present in the interior [135, 159]. In addition, a targeted allergological diagnosis is not possible in most cases, as the repertoire of mold allergen extracts that are commercially available for diagnosis is very limited and mainly comprises typical species from the outdoor environment. 

From a medical point of view, a visual inspection of a mold infestation is sufficient to initiate medically justified measures. The on-site inspection, ideally carried out by the doctor and persons with expertise in building physics, is of the greatest relevance. 

In the case of visible mold infestation, increased material moisture or physical/structural abnormalities (“moisture or water damage”), identification and quantification of mold in the interior is not necessary from a medical diagnostic and therapeutic point of view [775]. 

The medical differential diagnosis always has priority in the health assessment of mold exposure. As the effect of molds is primarily dependent on the disposition of the person affected, the delay in taking measures due to a lengthy mold identification can pose an increased risk for people who require special protection from mold exposure. Risk groups requiring special protection are

- Persons under immunosuppression according to the classification of the KRINKO at the RKI [349]
- Persons with severe influenza
- Persons with severe COVID-19
- Persons with CF (mucoviscidosis)
- Persons with bronchial asthma

**Key message 9, section 1.3 Key messages:**

**Risk groups requiring special protection are:**

**a) Persons under immunosuppression according to the classification of the Commission for Hospital Hygiene and Infection Prevention (KRINKO) at the Robert Koch Institute (RKI) [349]**

**b) Persons with severe influenza**

**c) Persons with severe COVID-19**

**d) Persons with cystic fibrosis (CF; mucoviscidosis)**

**e) Persons with bronchial asthma.**

For reasons of prevention only, medical examinations of the above-mentioned groups of people may rarely be indicated for risk assessment purposes if there are grounds for suspicion. 

A summary of the investigation methods for detecting mold exposure in indoor mold infestations, including moisture damage, can be found in Gabrio et al. (2015) [207]. This presentation is intended to provide physicians, but also environmental mycologists, indoor air quality experts, craftsmen, architects and building experts who have to commission and/or evaluate the corresponding measurements, with sound knowledge of the appropriate application and significance of the various measurement and examination methods. This provides a solid basis for commissioning and evaluating the relevant investigations. For more in-depth information on this topic, please refer to the relevant literature [135, 267, 297, 380]. 

### 3.5 Medical-clinical diagnostics 

Allergy history forms the basis of the step-by-step diagnostic scheme, followed by the skin test, the determination of allergen-specific IgE, and, at the top, organ provocation. This step-by-step scheme illustrates two things: the higher up the diagnostic procedure is, the more important it is, and the smaller the area, the less often it is indicated. 

**3.5.1 Allergy diagnostics**

The diagnosis of mold allergy does not differ from the diagnosis of other allergic diseases. A step-by-step procedure is usually carried out according to the classic step-by-step scheme, taking individual factors into account: medical history/physical findings/clinical examination – skin test – serum analysis or additional *in vitro* methods – provocation [676]. 

Allergic diseases caused by mold allergens can manifest as conjunctivitis, rhinitis, rhinosinusitis, allergic bronchial asthma, urticaria, EAA, or ABPA. Consequently, the differential diagnosis based on medical history and *in vitro*/*in vivo* laboratory diagnostics is of central importance. In individual cases, it is important to confirm the allergic reaction and identify the allergy trigger. There is a wide variety of *in vitro* tests that measure parameters of the cellular and humoral allergic reaction at different levels. However, the repertoire of mold allergen extracts that are commercially available is limited and mainly includes typical outdoor airborne species. 

Particularly in the case of *in vitro* tests, it should be noted that elevated mold-specific IgE concentrations, for example, may indicate sensitization to mold allergens, but are not the same as an allergic disease. A correct interpretation of the results can only ever be made in connection with the medical history, clinical manifestation and/or the results of the organ-specific provocation tests. Positive evidence of sensitization to mold fungi must be interpreted very critically in the causality assessment with regard to the exposure possibilities (ubiquitous outdoor air exposure, indoor exposure, occupational exposure). In the case of mold sensitization, it is rarely possible in everyday allergy-environmental medicine to reliably confirm the causal relationship between mold exposure in an indoor environment and a specific sensitization and related disease (rhinitis, conjunctivitis, asthma) [354]. 

The following requirements for the diagnosis of a mold allergy must be met [674]:

- A pathogenic mold antigen can be found in the private and professional environment.
- There is a definite temporal relationship between the allergic symptoms and the exposure to the mold allergen.
- There is an atopic predisposition.
- There is evidence for the formation of specific IgE against mold antigens.
- Measures to avoid mold allergens show clear clinical effects.

In principle, the same recommendations and guidelines apply to the diagnosis of a mold allergy as for other allergen sources that are the causes of an immediate-type allergy [565]. 

**Key message 13, section 1.3 Key messages:**

**The core elements of a type I allergy diagnosis are the medical history, skin prick test, determination of specific IgE antibodies, and provocation testing. In case of allergic bronchopulmonary aspergillosis (ABPA), the determination of specific IgG antibodies should also be performed. In the case of extrinsic allergic alveolitis (EAA), only the determination of specific IgG antibodies shall be performed serologically.**

Cross references:

- German Society for Pneumology and Respiratory Medicine (DGP). S2k guideline Diagnostics and therapy of adult patients with cough, version 3.0. Register number 020 - 003. Status: January 01, 2019. Valid until: December 31, 2023 (under revision); https://register.awmf.org/de/leitlinien/detail/020-003
- *Kardos P, Dinh QT, Fuchs K-H, Gillissen A, Klimek L, Koehler M, Sitter H, Worth H.* Guideline of the German Society of Pneumology and Respiratory Medicine on the diagnosis and treatment of adult patients with cough. Pneumology. 2019; *73:* 143-180; doi: 10.1055/a-0808-7409; https://www.thieme-connect.de/products/ejournals/abstract/10.1055/a-0808-7409
- *Renz H, Biedermann T, Bufe A, Eberlein B, Jappe U, Ollert M, Petersen A, Kleine-Tebbe J, Raulf-Heimsoth M, Saloga J, Werfel T, Worm M.* *In vitro* allergy diagnostics. Allergo J. 2010; *19:* 110-128. doi: 10.1007/BF03362255
- German Society for Pneumology and Respiratory Medicine (DGP). S2k guideline Specialist diagnosis and therapy of asthma, version 3.0. Register number 020-009. Status: March 01, 2023. Valid until: February 29, 2028; https://register.awmf.org/de/leitlinien/detail/020-009

**3.5.1.1 Serological tests**

*In vitro* serological tests include the detection of specific IgE antibodies in the case of IgE-mediated disease and the detection of specific IgG antibodies in the case of HP. The detection of elevated specific antibodies is a clear indication of sensitization, but is not synonymous with clinical relevance. The predictive value for clinical relevance can also increase with the level of the specific IgE response [735]. 

a) Detection of mold-specific IgE antibodies 

The undoubtedly most important and practical *in vitro* test is the determination of allergen-specific IgE antibodies (sIgE) in serum. Especially as more and more mold skin test solutions are being withdrawn from the market [345], serologic IgE determination is almost the only available test instrument, although it is often less sensitive than skin prick tests [326]. The use of a mold mixture (mx 1, consisting of *Alternaria alternata*, *Cladosporium herbarum*, *Aspergillus fumigatus,* and *Penicillium chrysogenum*) is sufficient to detect IgE reactions to all individual mold species included and may therefore be suitable as a screening tool. If mold-specific IgE is detectable, a history of possible exposure (indoor or outdoor) should be checked and possible co-sensitizations such as grass pollen or house dust mites, which represent an overlapping allergen exposure, should be investigated [329, 331]. When testing with total extracts, it should be borne in mind that Asp f 6 is a homologous allergen to Alt a 16 [611]. Although a large number of tests from different manufacturers exist, the selection of indoor-relevant mold diagnostics is very limited. The tests of the different manufacturers differ not only due to the test procedure (including the use of different detection methods such as ELISA, FEIA, RIA, the use of different allergen carriers such as chemically activated paper disc, microtiter plate, ImmunoCAP, chip technology or the use of liquid allergens), but also due to different allergen raw materials, allergen extract preparations and their standardization [433]. The value of *in vitro* diagnosis is determined by the diagnostic sensitivity and specificity of the test method, and here too the validity of allergy diagnostics is heavily dependent on the quality of the allergen extracts used and also on the method used. Despite the 113 mold allergens from a total of 30 mold species now described in the WHO/IUIS allergen database (www.allergen.org: as of 10/2021) [332], only eight individual allergens in recombinant form from the three outdoor air species *Alternaria alternata* (rAlt a 1, rAlt a 6), *Aspergillus fumigatus* (rAsp f 1, 2, 3, 4, 6) and *Cladosporium herbarum* (rCla h 8) are currently available for molecular diagnostics [332]. Improved mold IgE diagnostics through the availability and use of mold-typical marker allergens (e.g., subtilisin-like proteases [332]) with strong IgE binding would be desirable. 

The determination of total IgE in conjunction with the determination of specific IgE can be useful as a supplementary parameter for the assessment of sIgE values [342]. However, the determination of total IgE can never rule out or prove specific sensitization and is not useful as the sole validation. 

For the diagnosis of ABPA, a type of allergy with components of a type I, type III and type IV reaction, which is usually (co-)caused by *A. fumigatus,* the determination of *A. fumigatus*-specific IgE as well as the determination of total IgE and *A. fumigatus-specific* IgG is useful (see specific IgG determination). Recombinant *A. fumigatus* single allergens (rAsp f 1-4, 6) are useful for further differential diagnostics. The combination of rAsp f 2 + rAsp f 4 + rAsp f 6, the three intracellular proteins, is considered an indicator of ABPA for the specific IgE determination, while sensitization to rAsp f 1 and/or rAsp f 3 (secretory proteins) is not a clear indicator of allergic asthma. Serologically positive findings for rAsp f allergens can also occur in other diseases, such as CF [118]. A meta-analysis [479], which summarized the results of the use of recombinant Asp f allergens in 26 studies (with a total of 1,694 patients), showed that IgE against Asp f 1 or Asp f 3 has the highest sensitivity (96.7% in asthmatics and 93.3% in CF patients) to differentiate ABPA patients from these patients, but Asp f 4 or Asp f 6 had the highest specificity with 99% in asthmatics versus Asp f 6 alone with 98% in people with CF. The more recent work by Muthu et al. (2020) [480] also supports the assessment that Asp 1 has a very high sensitivity and specificity for ABPA. 

Summary:

- Detection of allergen-specific IgE indicates a specific sensitization, but not necessarily a disease; the result can only be correctly interpreted in conjunction with the patient’s medical history, clinical manifestation and the results of organ-specific provocation tests. Positive reactions caused by cross-sensitization are only partly clinically relevant.
- Quantitative comparisons of the results from different test systems are difficult to make (international standardization is still lacking).
- Improvement of reagent quality through standardization of allergens and definition of minimum requirements for the allergen carrier material (determination of diagnostic efficiency) is required.
- Extracts of indoor-relevant molds should also be commercially available in sufficient quality.
- The spectrum of available individual allergens of the relevant molds should be expanded.

**Key message 12, section 1.3 Key messages:**

**As polysensitized individuals, atopic patients often also have IgE antibodies against molds, although this does not necessarily mean that they are ill. The clinical severity of the allergic reaction does not correlate with the level of the specific IgE titer.**

**Key message 14, section 1.3 Key messages:**

**The detection of specific IgE or a positive reaction in the skin test initially only mean that a specific sensitization to corresponding allergens is present. A clinically relevant allergy only becomes apparent in connection with typical allergic symptoms.**

**Key message 15, section 1.3 Key messages:**

**A negative result of a skin test or a specific IgE test for molds does not reliably exclude sensitization to molds. The reasons for this include the varying composition and quality of test extracts or the absence of relevant allergens.**

b) Determination of mold-specific IgG 

The determination of specific IgG antibodies in connection with the diagnosis of mold allergy of the immediate type (type I allergy) has no diagnostic significance, since IgG antibodies as a physiological response of the immune system rarely have a pathogenetic significance, and is therefore not recommended [341]. 

Only in cases of suspected ABPA (type I, III allergy) or EAA (type III, IV allergy) the determination of mold-specific IgG antibodies is a useful part of the diagnostic procedure and is recommended [508]. 

In ABPA, in addition to an increase in total IgE and specific IgE against *A. fumigatus* (see above), there may also be an increase in specific IgG against *A. fumigatus*. The latter is often significantly elevated compared to patients with allergic sensitization to *A. fumigatus* and is recommended for ABPA differential diagnostics. However, *Aspergillus* IgG is only a minor criterion in newer diagnostic algorithms in contrast to the specific IgEs against *Aspergillus fumigatus* [415]. 

For the quantitative assessment of specific IgG concentrations (stated in mg_A_/L), it must be taken into account that, in contrast to specific IgE diagnostics, there is no uniform cut-off value, meaning that a specific reference value or range must be determined for each antigen and for each measurement method. Furthermore, there are no defined cut-off values that clearly indicate pathological changes. For 32 typical commercially available skin test antigens, including numerous molds, reference ranges were determined specifically for a detection system using 121 sera from healthy unexposed control subjects [566]. 

**Key message 16, section 1.3 Key messages:**

**The determination of specific IgG antibodies in connection with the diagnosis of an immediate-type mold allergy (type I allergy) has no diagnostic significance and shall therefore not be performed. This also applies to the detection of immune complexes, e.g., using the Ouchterlony test.**

c) Eosinophilic cationic protein (ECP) 

Elevated ECP concentrations reflect the activation status of the eosinophilic leukocytes, but do not allow the classification or clarification of specific allergic diseases. There is no specific indication for this non-specific marker of activation and recruitment of eosinophil granulocytes for the detection of a mold allergy. 

**Key message 18, section 1.3 Key messages:**

**The determination of eosinophil cationic protein (ECP) and β-1,3-D-glucan (BDG) in serum has no indication and shall not be performed in medical diagnostics in the case of mold exposure.**

d) Immune complexes 

The examination of immune complexes, e.g., by double immunodiffusion according to Ouchterlony, is limited to special clinical pictures from the group of type III allergic reactions such as HP and has no place in the diagnosis of mold exposure (see mold-specific IgG determination above). 

**Key message 16, section 1.3 Key messages:**

**The determination of specific IgG antibodies in connection with the diagnosis of an immediate-type mold allergy (type I allergy) has no diagnostic significance and shall therefore not be performed. This also applies to the detection of immune complexes, e.g., using the Ouchterlony test.**

e) Galactomannan in serum 

Galactomannan, a heteropolysaccharide, is a cell wall component of the mold genus *Aspergillus* and can circulate in serum in invasive pulmonary aspergillosis (IPA). The detection of galactomannan in BAL can be measurable before the appearance of galactomannan in the serum and thus represent an early sign of aspergillosis. A negative result does not rule out aspergillosis; repeated tests at short intervals are recommended. Serological test methods are available for the detection of galactomannan from serum and are based on antigen detection using classic sandwich ELISAs. This test is only indicated for the diagnosis of IPA [435, 741]. 

**Key message 17, section 1.3 Key messages:**

**Galactomannan in serum shall only be performed for the diagnosis of suspected invasive pulmonary aspergillosis (IPA), otherwise there is no indication in the diagnosis of mold exposure.**

f) β-1,3-D-glucan (BDG) in serum 

The principle of this test method is based on a modification of the Limulus amoebocyte lysate test. It is a microtiter plate-based antigen detection test for the detection of (1→3)-β-D-glucan in serum. This test procedure has not been validated for plasma, cerebrospinal fluid, pleural punctate and joint punctate, so its validity is questionable. BAL is not a suitable material, as it is usually contaminated with yeasts from the oral cavity, which almost always leads to positive test results. A chromogenic reagent is used for detection, which eliminates factor C in the procoagulant system of the Limulus amoebocyte lysate pathway. This opens the way to activate factor G, a serine protease enzyme, in the presence of (1→3)-β-D-glucan. The inactive procoagulant system becomes an active one, which then cleaves off a chromogenic peptide substrate, forming a light-absorbing chromophore. 

The test is technically demanding and is primarily used to rule out invasive mycoses. It is not indicated for use in connection with indoor mold exposure [190]. 

**Key message 18, section 1.3 Key messages:**

**The determination of eosinophil cationic protein (ECP) and β-1,3-D-glucan (BDG) in serum has no indication and shall not be performed in medical diagnostics in the case of mold exposure.**

g) Mycotoxins in serum 

With regard to intoxication by airborne mold toxins, a diagnosis is only possible to a limited extent [734]. In the USA, macrocyclic trichothecenes were detected in sera of individuals exposed to *Stachybotrys chartarum* in indoor environments [64, 274, 680]. For this purpose, an immunochemical detection method was used (trichothecene ELISA), in which 23 sera from test persons as well as a control serum were positive. Although an ELISA is a very sensitive method with detection limits in the range of ppb (equivalent to ng/mL or g), non-specific reactions and cross-reactions can never be completely ruled out. 

In recent years, great progress has been made in the analysis of mycotoxins in serum and urine using HPLC mass spectrometry and has already been successfully used for human biomonitoring studies. However, the studies relate to the more relevant exposure via the food pathway. 

Systematic studies on inhalation exposure indoors are not available. 

With the current state of analytical possibilities, mycotoxins from indoor pollution can neither be validly determined nor evaluated by human biomonitoring. The determination of mycotoxins in blood, serum or urine has no significance for practical medicine / routine diagnostics and must currently be limited to scientific questions. 

**Key message 24, section 1.3 Key messages:**

**Human biomonitoring of mycotoxins has no indication in medical diagnostics for indoor mold exposure, and shall therefore not be performed.**

**3.5.1.2 Cellular test systems**

For all cellular test systems based on IgE-sensitized basophilic granulocytes or on lymphocytes from peripheral blood or whole blood, the following has applied to date: they are methodologically complex, costly, generally poorly suited for shipping samples, and challenging to perform and interpret. Cellular *ex vivo* diagnostics with basophil granulocytes is not suitable for routine diagnostics and belongs to specialized allergy diagnostics. However, these tests can be an useful addition in individual cases [573] and should be used in the case of a clear suspicion of an IgE-mediated allergy and unclear previous diagnostic findings and scientific questions. Compliance with the necessary pre-analytical conditions is a prerequisite for valid results. However, the selection of commercial mold allergen extracts available specifically for these tests, which are used as stimuli, is very limited, and here too the result of the test depends on the quality of the antigen used (extract or individual allergen) [329, 345]. 

Cellular test systems based on IgE-sensitized basophilic granulocytes use different parameters (“readouts”) for test evaluation, but have identical principles. The detection of released mediators (e.g., histamine, leukotrienes) or the expression of surface antigens (e.g., CD203c, CD63) after *in vitro* stimulation with allergens are possible readouts. 

Positive results after titrated allergen incubation serve as an indirect measure of the cellular bound specific IgE. The considerable excess of bound IgE on basophils and its high affinity for the FcεRI receptor result in a high analytical sensitivity of these test systems, which can outperform both specific serological IgE methods and skin tests. Rare indications for these tests with the target cell “basophil granulocyte” are therefore samples with extremely low total IgE and unsuccessful specific serological IgE detection in the case of suspected sensitization or exotic allergens (whereby the availability and quality of the extracts must be taken into account). 

Cross references:

- *Renz H, Biedermann T, Bufe A, Eberlein B, Jappe U, Ollert M, Petersen A, Kleine-Tebbe J, Raulf-Heimsoth M, Saloga J, Werfel T, Worm M.* *In vitro* allergy diagnostics. Allergo J. 2010; *19:* 110-128; doi: 10.1007/BF03362255

a) Basophil degranulation test and histamine release, histamine liberation test (HLT) 

The basophil degranulation test measures allergen-induced release of the granule-derived preformed mediator histamine. 

Histamine release serves as an indirect measure of cellular-bound specific IgE (see above), which has a longer half-life compared to serologic IgE. Histamine is determined using spectrofluorometric, enzyme or radioimmunological methods. While the determination of histamine has declined due to the more complex methodology, the flow cytometric measurement of surface antigens on basophils (e.g., CD203c, CD63) has gained in importance as an activation marker. 

**Key message 19, section 1.3 Key messages:**

**The basophil degranulation test and histamine release (HLT = histamine liberation test), the basophil activation test (BAT) using flow cytometry and the determination of other mediators (sulfidoleukotriene release test, cellular antigen stimulation test (CAST-ELISA)) are used in special diagnostics, but should not be performed in basic allergy diagnostics.**

b) Basophil activation test (BAT) using flow cytometry (FlowCAST) 

The basophil activation tests (BAT) are based on the flow cytometric detection of activation markers on basophil granulocytes. For IgE-mediated reactions, the markers CD63 and CD203c have primarily been used to date. The quantitative determination of surface markers is carried out using flow cytometry (FlowCAST); a further development is the so-called Flow2CAST, in which CCR3 (eotaxin receptor) is used as a selection criterion for better discrimination of basophils. The test is useful for inhalation allergens, especially in cases where the skin test and measurements of specific IgE cannot be carried out. Rare indications also include samples with low total IgE, unsuccessful specific serological IgE detection and possibly a negative skin test in the case of suspected sensitization or exotic allergens. 

In principle, soluble, non-cytotoxic substances (commercially available allergens, but also crude extracts) can be used as allergens, which should be used in different concentrations (dose series). However, only a few mold test allergens (*Penicillium chrysogenum (notatum*), *Cladosporium herbarum*, *Aspergillus fumigatus*, *Alternaria alternata*) are available, which often do not represent the colonizing fungal spectrum. 

A BAT can be helpful in local allergic rhinitis with positive nasal provocation and negative IgE detection and negative prick test. 

The sensitivity of the BAT correlates well with the result of the nasal provocation test in allergic rhinitis [158]. 

The prerequisite for carrying out a BAT is a sufficient number of basophils in the EDTA whole blood (at least 150 basophils in 0.1 ml of blood). 

**Key message 19, section 1.3 Key messages:**

**The basophil degranulation test and histamine release (HLT = histamine liberation test), the basophil activation test (BAT) using flow cytometry and the determination of other mediators (sulfidoleukotriene release test, cellular antigen stimulation test (CAST-ELISA)) are used in special diagnostics, but should not be performed in basic allergy diagnostics.**

c) Determination of other mediators (sulfidoleukotriene release test, cellular antigen stimulation test (CAST-ELISA)) 

In this CAST ELISA, *de novo* synthesized sulfidoleukotrienes are measured, which are formed after preactivation with interleukin 3 and allergen contact in the sensitized person (e.g., cellular antigen stimulation test (CAST)). After completion of allergen incubation and centrifugation, the leukotrienes in the supernatants are determined using an ELISA. 

A positive result for an allergen, i.e., indirect evidence of sensitization, is only indicative of a clinically significant allergy if there is a clear anamnestic connection and/or a positive provocation test. The sulfidoleukotriene release test is a complex cellular test system whose significance lies in special and not basic allergy diagnostics. 

**Key message 19, section 1.3 Key messages:**

**The basophil degranulation test and histamine release (HLT = histamine liberation test), the basophil activation test (BAT) using flow cytometry and the determination of other mediators (sulfidoleukotriene release test, cellular antigen stimulation test (CAST-ELISA)) are used in special diagnostics, but should not be performed in basic allergy diagnostics.**

d) Lymphocyte stimulation test (LST) / Lymphocyte transformation test (LTT) 

The lymphocyte transformation test (LTT) is a laboratory procedure for the detection of antigen-specific T lymphocytes. It is used in immune function diagnostics in medicine. For a few years now, it has also been used predominantly in the context of scientific, but rarely in clinical questions in allergology for the detection of certain delayed type IV allergic reactions (e.g., drug allergy). A fundamental problem with LTT is that it is not possible to distinguish between a “physiological” response to an antigen and an “allergic” T cell response. Proliferation is therefore merely an expression of a normal confrontation of the organism with an (already known) antigen and therefore not an indicator of clinically relevant sensitization. When using protein antigens in particular as stimuli, it should be noted that contamination of the antigen with endotoxins can induce false-positive proliferation. As mold allergens do not induce type IV sensitization, LTT for molds are not indicated as a diagnostic procedure [355]. 

**Key message 20, section 1.3 Key messages:**

**Lymphocyte transformation tests (LTT) for molds are not indicated as a diagnostic procedure [355] and shall therefore not be performed.**

e) Whole blood test 

In order to describe the immune reactivity due to mold exposure, the whole blood test, i.e., the cellular analysis with blood from exposed persons, has recently gained importance as a possible additional instrument [785]. The use of whole blood without further cell isolation steps is a relatively simple experimental approach to gain insight into the immune response [42, 152]. A whole blood test can be used in two different ways: either as a tool to describe the pyrogenic and proinflammatory properties of bioaerosol/dust samples (stress monitoring) or to analyze individual changes in a person’s immunological reactivity (load monitoring) with their fresh blood [398]. In general, the whole blood test is a two-step test. In step 1, the blood is incubated with the appropriate stimuli (including mold extracts), and in step 2 the cytokine release (IL-1β, IL-6, IL-8) in the cell-free supernatant is quantified by ELISA [556]. A recent pilot study using whole blood from 29 mold-exposed and 19 non-mold-exposed individuals suggests that *in vitro* stimulation of fresh blood from individual subjects with mold extracts and subsequent cytokine release reflected disease-related cellular differences but did not indicate mold exposure [399]. In combination with other data, Whole blood test can be a helpful and interesting tool in research, for example, in describing the complex immune response to molds. Although statistically significant differences can be found on a group basis [556], the Whole blood test does not help in the assessment of mold exposure in individual cases. The Whole blood test is therefore not a suitable instrument for validating mold exposure and cannot be recommended for diagnostic purposes. 

**Key message 21, section 1.3 Key messages:**

**The whole blood test is not a suitable instrument for detecting mold sensitization and shall therefore not be performed.**

**3.5.1.3 Provocation tests**

If the medical history, physical examination, and serology do not provide a clear diagnosis of a mold allergy, a provocation test may be indicated if this has important consequences for therapy, prevention, and/or compensation [220]. In this test, the patient is confronted with the allergens in question by natural means in order to provoke an immediate allergic reaction (type I) with the corresponding typical symptoms under controlled conditions. An organ-related provocation test is used to confirm the clinical relevance of existing sensitization or supposedly observed symptoms. 

Similarly, the study by O’Driscoll et al. (2009) [511] also showed that the correlation between skin test results and specific IgE findings in mold diagnostics is unsatisfactory. It is therefore recommended that all available methods, both skin testing and serological examinations, should be used for a target-oriented diagnosis and that test extracts – if available – from different manufacturers should also be used. The sole use of a serological IgE determination for the detection of mold sensitization does not appear to be sufficient in terms of sensitivity [328]. 

However, tests with commercial extracts should not be avoided as they are easy to handle and their biological quality is controlled by batch tests of the Paul-Ehrlich-Institut. 

It has proven useful to compile fixed test series for specific questions. The selection of standard aeroallergens to be tested must take into account individual, occupational and regional circumstances [590]. 

False negative results often occur in comparison with the medical history and skin testing [395]. 

The results of tests with rarer allergens and unstable mold allergens are particularly critical. 

It follows that negative *in vitro* and *in vivo* test results do not rule out sensitization or allergy to molds. 

In the complete absence of IgE-mediated sensitization to the largely standardized skin test solutions of common environmental allergens, mold allergy is a rarity. 

With every provocation test, there is a risk of a severe allergic reaction and, in the worst case, of anaphylactic shock. Doctors and nursing staff must therefore have the relevant experience, and must have emergency equipment for testing and provocation and be familiar with the treatment of emergencies [395]. 

Provocation tests should not be carried out in the case of a high degree of sensitization, acute inflammatory diseases of the nose, acute allergic reactions in other organs, severe general reactions, taking medication that increases the risk of intolerance reactions (ACE inhibitors, β-blockers) [674], as well as in children under 5 years of age. 

**Test materials**

Currently, only a few commercial mold allergen test extracts are available from individual manufacturers. As studies by Kespohl et al. 2013 [325] using detailed biochemical and immunological analyses demonstrate, mold allergen extracts show a very high variability in allergen composition, and preparations of one mold species from different manufacturers are not comparable. The skin test extracts of the outdoor mold *Alternaria* are an exception. With regard to storage and shelf life of the test substances, the usual general conditions for skin tests also apply to the mold extracts (storage of the extracts in the refrigerator at an average temperature of 4 °C, observation of expiry date of the test solutions [590]). 

According to EU Directive 2001/83/EC, Article 1(4b), test allergens are defined as medicinal products. The associated approval procedure tends to mean that mold extracts in particular, which are very complex and cost-intensive to produce, are no longer commercially available and diagnostics are therefore more restricted. Sensible strategies for the future in the interests of those affected should be found as quickly as possible. 

a) Skin testing 

Skin tests form the basis of allergological diagnostics after the medical history and can be carried out quickly and relatively inexpensively. As a rule, they are sufficiently informative and have a low complication rate. Skin tests should be performed in accordance with the relevant German and European position papers [608]. 

In skin tests, a distinction is made between epicutaneous (patch test, rub test) and cutaneous tests (scratch, prick, intracutaneous test). The allergen concentration of solutions for the intradermal test is usually 100 – 1,000 times lower than that of prick test solutions. However, commercial intradermal test solutions are no longer available (as of June 2015), making this diagnostic method for detecting mold sensitization obsolete. An allergic reaction is triggered by applying an allergen dose to or into the skin. If inhalation allergies to mold spores are suspected, a prick test is usually carried out. 

The size and nature of the reacting area (erythema, wheal) are used as a measure of the degree of sensitization of the organism [590]. 

The maximum histamine reaction occurs within 15 minutes. Allergen-induced reactions reach their maximum after 15 to 20 minutes. Regression usually occurs within 1 – 2 hours. A few hours later, delayed immediate-type reactions can occur, which appear as wheals or erythema. Late-type reactions are also possible, which appear within hours to a few days after the test, e.g., as a reddened papule or eczema [590]. 

In all tests, these late-type reactions can occur as a delayed reaction (after 6 –  24 hours) or as a late reaction (up to 48 hours) in the case of both negative and positive immediate reactions, which is why the observation interval should not be less than 24 hours (possible as patient self-observation). A positive result in skin tests requires both functioning immunological mechanisms and the ability of the skin to react. If medication is taken that influences the immune response (e.g., antihistamines, corticoids) and in the case of eczematous or urticarial skin diseases, the result is doubtful. 

The inner sides of the forearms or the back are suitable areas of skin to test. 

**Key message 14, section 1.3 Key messages:**

**The detection of specific IgE or a positive reaction in the skin test initially only mean that a specific sensitization to corresponding allergens is present. A clinically relevant allergy only becomes apparent in connection with typical allergic symptoms.**

**Key message 15, section 1.3 Key messages:**

**A negative result of a skin test or a specific IgE test for molds does not reliably exclude sensitization to molds. The reasons for this include the varying composition and quality of test extracts or the absence of relevant allergens.**

Cross references:

- *Ruëff F, Bergmann K-C, Brockow K, Fuchs T, Grübl A, Jung K, Klimek L, Müsken H, Pfaar O, Przybilla B, Sitter H, Wehrmann W.* Skin tests for the diagnosis of immediate allergic type reactions. Guideline of the German Society for Allergology and Clinical Immunology (DGAKI) in coordination with the Medical Association of German Allergists (ÄDA), the Professional Association of German Dermatologists (BVDD), the German Dermatological Society (DDG), the German Society for Ear, Nose and Throat Medicine and Head and Neck Surgery (DGHNOKHC), the German Society for Pneumology and Respiratory Medicine (DGP) and the Society for Pediatric Allergology and Environmental Medicine (GPA). Allergo J. 2010; *19:* 402-415; https://dgaki.de/wp-content/uploads/2010/05/Leitlinie_Hauttests-at-immediate-type-reactions2010.pdf
- *Ruëff F, Bergmann K-C, Brockow K, Fuchs T, Grübl A, Jung K, Klimek L, Müsken H, Pfaar O, Przybilla B, Sitter H, Wehrmann W.* Skin tests for the diagnosis of immediate-type allergic reactions. Guideline of the German Society for Allergology and Clinical Immunology (DGAKI). Pneumology. 2011; *65:* 484-495; doi: 10.1055/s-0030-1256476; https://www.thieme.de/statics/dokumente/thieme/final/de/dokumente/zw_pneumologie/Hauttest_Allergie.pdf

b) Nasal provocation test (NPT) 

The nasal provocation test (NPT) enables the reproduction of an allergic reaction on the organ of manifestation under standardized conditions and is considered a simple and safe procedure with high specificity and sensitivity [10, 34, 227, 608]. Evaluation and implementation are carried out according to the standards of the position paper of the EAACI (European Academy of Allergology and Clinical Immunology) [33]. 

Inhalation allergies to mold spores are usually accompanied by persistent respiratory symptoms. This can make a clear anamnestic reference difficult. In this context, the NPT can confirm or refute the suspected diagnosis of an allergic reaction of the respiratory tract. The NPT is also indicated if there are contraindications to a skin test, for suspected Local Allergic Rhinitis (LAR) and for monitoring the progress of therapies such as allergen-specific immunotherapy (SIT). It is also true for provocation test solutions that fewer and fewer test substances are available. At the time of writing this guideline, the guideline “Standardized application of nasal and conjunctival provocation test on allergic diseases of the Upper Airways” is currently being revised. Reference is made to this guideline for the performance of the NPT. 

Cross references

- German Society for Allergology and Clinical Immunology e.V. (DGAKI). Sk2 guideline Standardized application of nasal and conjunctival provocation test on allergic diseases of the Upper Airways. Planned completion: 30.06.2023; https://register.awmf.org/de/leitlinien/detail/061-009

c) Conjunctival provocation test (CPT) 

The CPT should only be performed in a symptom-free state; standardized prick test solutions of 1 : 10 are generally used, possibly in a higher dilution [235]. First, a control solution is placed in the lower conjunctival sac and a possible reaction is waited for 10 minutes (control eye). Then a drop of the allergen test solution is applied to the inferior conjunctiva of the other eye (test eye). A positive test result is indicated by increasing itching, increased tear secretion, foreign body sensation, photophobia, etc. (Table 10). 

As soon as stage II-III has been reached, the test is considered positive and the patient can be spared further, more severe reactions by rinsing the conjunctival sac with physiological saline solution and administering eye drops (antihistamines, vasoconstrictors). 

The CPT is significantly less standardized than the NPT, does not directly record the reaction of the airway mucosa as a manifestation organ for aeroallergens and is therefore not a standard test method. 

CPT may be indicated if

- predominantly conjunctival symptoms are present.
- NPT is not possible in the case of nasal complaints due to contraindications or recent endonasal surgery.

Cross references:

- German Society for Allergology and Clinical Immunology e.V. (DGAKI). Sk2 guideline Standardized application of nasal and conjunctival provocation test on allergic diseases of the Upper Airways. Status: May 28, 2020. Planned completion: December 31, 2024; https://register.awmf.org/de/leitlinien/detail/061-009

d) Bronchial provocation test 

A bronchial provocation test may be indicated if the diagnosis cannot be made by a combination of exposure testing and less invasive diagnostic tools such as a history of asthma symptoms and antibody detection and skin testing. Particularly in the case of indoor molds, which occur perennially, a medical history is generally not helpful. There is an optional indication to confirm the diagnosis prior to hyposensitization and if a connection with a specific exposure must be assessed in court [220]. In analogy to other inhalation allergens, the degree of sensitization can be taken into account for orientation purposes. In this respect, the bronchial provocation test is of great importance in cases of suspected allergic perennial asthma caused by indoor molds. The choice of allergen should be based on the sensitization spectrum. The evidence for provocation testing in the absence of evidence of sensitization is insufficient so that no recommendation can be made. 

Allergen extracts from molds are generally suitable for provocation tests due to their sufficient solubility. Since it is also not possible to quantify molds in native materials sufficiently with reasonable effort, testing with native material in the laboratory is not a suitable method. Testing in and away from potentially contaminated rooms can provide an indication of an allergen source, but cannot be assessed with regard to the triggering agent. 

The range of commercial extracts for provocation tests is becoming increasingly limited. Performance must be based on the relevant guideline (Guideline for the performance of bronchial provocation tests with allergens, Part I and II 2001). 

When assessing provocation tests with allergens, both false-positive and false-negative reactions must always be expected. In the absence of a clinically relevant gold standard, a statement about sensitivity and specificity is generally problematic and is made particularly difficult in the case of molds by the inadequate investigations into the quality of the test extracts. More recent studies from Finland on occupationally exposed persons show that provocation testing with commercial mold extracts may be significantly more sensitive than the sensitization test [317]. These data need to be confirmed. The assessment of the provocation reaction with mold provocations is a challenge in this respect, also because isolated late reactions have frequently been described [317]. 

Cross references:

- *Gonsior E, Henzgen M, Jörres RA, Kroidl RF, Merget R, Riffelmann F-W, Wallenstein G.* Guideline for the performance of bronchial provocation tests with allergens - Part I. German Society for Allergology and Clinical Immunology. Allergo J. 2000; *9:* 193-199. https://dgaki.de/wp-content/uploads/2010/05/Leitlinie_BronchialeProvokationAllergenenTeilA20001.pdf
- *Gonsior E, Henzgen M, Jörres RA, Kroidl RF, Merget R, Riffelmann F-W, Wallenstein G.* Guideline for the performance of bronchial provocation tests with allergens - Part II. German Society for Allergology and Clinical Immunology. Part B: Allergo J. 2001; *10:* 257-264. https://dgaki.de/wp-content/uploads/2010/05/Leitlinie_BronchialeProvokationAllergenenTeilB2001.pdf
- *Gonsior E, Henzgen M, Jörres RA, Kroidl RF, Merget R, Riffelmann F-W, Wallenstein G.* Guideline for the performance of bronchial provocation tests with allergens. Pneumology. 2002; *56:* 187-198; doi: 10.1055/s-2002-20553
- German Society for Occupational and Environmental Medicine (DGAUM). S2k guideline Workplace-based inhalation test (AIT) - specific inhalation challenge (SIC), version 3.1. Register number 002 - 026. Status: January 25, 2021. Valid until: January 24, 2026; https://register.awmf.org/de/leitlinien/detail/002-026

**3.5.1.4 Allergological differential diagnostics (pollen, house dust mites, indoor allergens)**

In the case of non-specific health conditions associated with visible or hidden mold damage, it should be noted that the symptoms reported in these cases (e.g., tiredness, headaches, mucous membrane irritation) can also occur with a variety of other illnesses and in connection with other indoor pollution (e.g., VOCs, formaldehyde, wood preservatives, insecticides, tobacco smoke). A home visit is generally recommended for orientation, if necessary, together with an indoor air quality and building expert. 

As molds usually occur indoors at the same time as other allergens, it is problematic to differentiate mold-specific effects. It has been shown, for example, that early exposure to house dust mites may lead to symptoms in the first year of life that correspond to an obstruction of the upper airways [736]. Antigens of house dust mites, the cat antigen Fel d 1, antigens introduced into the interior from outside as well as bacteria and endotoxins must also be taken into account [252]. House dust mites (and also bacteria) are a particular confounder because, like molds, they occur more frequently in rooms with higher air and material humidity. What is particularly problematic is that numerous allergens of biological origin are subject to a similar seasonal cycle as the molds typical in the outside air. In summer/late summer, for example, there is not only an increase in mold spores (especially *Cladosporium* and *Alternaria*) in outdoor and indoor air, but also numerous grass and herb pollens. Diagnostic clarification of other indoor allergens is therefore advisable, e.g., by means of specific IgE determination or a skin test. 

**Figure 4:**
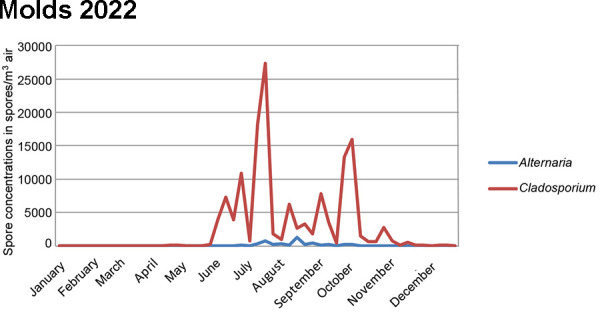
Annual course of the occurrence of mold spores in the outdoor air, spore concentrations as weekly values in spores/m^3^ air, pollen trap in Leverkusen. Source: Mülleneisen 2023, unpublished data. A volumetric spore trap based on the Hirst principle was used to determine the concentration [269] with associated microscopic analysis of the samples. The measurement method is standardized [738].

Figure 4 shows an example of the seasonal mold spore flight for *Alternaria* and *Cladosporium* for the measuring station in Leverkusen, which overlaps the grass/herb season and makes it difficult to differentiate between allergy symptoms in summer based solely on the medical history. 

**Key message 11, section 1.3 Key messages:**

**In principle, a large number of mold species can cause sensitization and allergies in case of corresponding exposure. Compared to other environmental allergens, however, the allergenic potential is to be regarded as lower overall [** 241 **,** 255 **].**

**3.5.2 Functional diagnostic procedures**

**Tear film break-up time**

The tear film break-up time, which was determined, for example, in a study by Norbäck et al. [503] shows an association with fungal DNA in classroom dust, is not suitable for individual medical diagnostics to objectify indoor mold-associated eye complaints due to a variety of other influencing factors. 

**Pulmonary function diagnostics**

Studies show an association between indoor mold exposure and changes in lung function in healthy indoor users [259, 500]. In individual medical diagnostics, however, no indoor mold-associated lung dysfunction can be diagnosed with lung function diagnostics without specific provocation. 

**NO in the exhaled air**

Scientific studies have shown an association between indoor mold and NO in the exhaled air of healthy people [83, 478, 502]. In individual medicine, the determination of NO in exhaled air cannot be used to diagnose an indoor mold-associated reaction of the respiratory tract. 

**Key message 25, section 1.3 Key messages:**

**The following diagnostic methods shall not be used for indoor mold exposure because there is insufficient scientific evidence (without claim of completeness): Detection of molds in the blood, determination of IgA antibodies directed against molds, determination of lymphocyte subpopulations, determination of cytokines, determination of oxidative stress, visual contrast sensitivity test (VCS test), tear film break-up time.**

**3.5.3 Infectiological diagnostics**

Systemic mycoses: 

For the procedure for mold infections, please refer to the relevant guideline. 

**Key message 23, section 1.3 Key messages:**

**Microbiological, immunological, molecular biological and radiological methods are core elements of mold infection diagnostics and shall be used depending on the indication.**

Cross references:

- Onkopedia Guideline - Invasive fungal infections - Diagnostics. Status: 2018

Working Group on Infections in Hematology and Oncology (AGIHO) of the German Society for Hematology and Medical Oncology (DGHO) 

*Ruhnke M, Behre G, Buchheidt D, Christopeit M, Hamprecht A, Heinz W, Heussel CP, Horger M, Kurzai O, Karthaus M, Löffler J, Maschmeyer G, Penack O, Rieger C, Rickerts V, Ritter J, Schmidt-Hieber M, Schuelper N, Schwartz S, Ullmann A, Vehreschild JJ, von Lilienfeld-Toal M, Weber T, Wolf HH.* Diagnosis of invasive fungal diseases in haematology and oncology: 2018 update of the recommendations of the infectious diseases working party of the German society for hematology and medical oncology (AGIHO). Mycoses. 2018; *61:* 796-813; doi: 10.1111/myc.12838, for the Infections in Hematology and Oncology Working Party (AGIHO) of the German Society for Hematology and Medical Oncology (DGHO); https://www.onkopedia.com/de/onkopedia/guidelines/invasive-pilzinfektionen-diagnostik/@@guideline/html/index.html

**3.5.4 Toxicological diagnostics**

**Mycotoxins**

Approximately 400 mycotoxins are known as secondary metabolites of molds [199]. Due to their chemical diversity, they cannot be detected in the environment (environmental monitoring) or in human biological materials (blood or urine) (human biomonitoring)) by a single analytical method. This requires different standardized sampling and measurement methods. 

The routine determination of mycotoxins in indoor air (environmental monitoring) or in mold-contaminated building materials has no medical diagnostic significance, as reliable data on inhalation toxicity and respirable concentrations in the air as well as standardized sampling and testing methods are still lacking, nor is it relevant for a remediation decision, as any massive mold infestation must be removed immediately, regardless of whether mycotoxins have been formed or not [91, 199, 286]. 

**Key message 4, section 1.3 Key messages:**

**In medical diagnostics for mold exposure, environmental monitoring of mycotoxins in indoor air and house dust has no indication.**

To date, only a few mycotoxins can be reliably detected in human biological materials). 

Meanwhile, mycotoxins from food-related intake and occupational exposure [172, 195, 264, 527, 709] can be reliably quantitatively detected in human biological materials [31, 185, 194, 199]. Nevertheless, this requires standardized criteria for sampling of human biological materials and analytical methods [239, 730]. There is still no indication for the determination of mycotoxins in human biological materials in medical diagnostics in patients with indoor mold exposure [286]. 

**Key message 24, section 1.3 Key messages:**

**Human biomonitoring of mycotoxins has no indication in medical diagnostics for indoor mold exposure, and shall therefore not be performed.**

**Microbial volatile organic compounds (MVOC)**

The term MVOC refers to volatile organic compounds that are formed by molds and bacteria. The metabolism of molds and bacteria produces numerous volatile metabolites that are responsible for the “mold smell” as OVOCs. Some substances have quite pleasant odor notes, for example 1-octen-3-ol has a typical mushroom smell, 2-heptanone a fruity smell and the terpene geosmin an earthy smell. Together, these substances produce a musty, typically “moldy” odor. This is perceived as unpleasant and is often associated with “spoilage” and “uncleanliness”. Sometimes those affected fear that the odorous substances themselves pose a toxic health risk. Many MVOCs have osmophoric groups (carbonyl, S, N or OH groups) and have very low odor thresholds [418, 472, 599]. 

It must be taken into account that for many so-called MVOCs there are other sources in addition to microbial ones (tobacco smoke, cooking, baking, roasting, potted plant soil, compost bins, etc.) [100, 621, 622, 623, 626]. 

It has not yet been clarified whether so-called MVOCs in concentrations in the lower µg/m^3^ range occurring indoors have biological signaling effects [226, 256]. Olfactory-psychological coupling reactions with unspecific complaints are possible with corresponding cacosmic perceptions, but toxic reactions are unlikely [360, 361]. 

Determining the MVOC concentration in indoor air is not useful for medical diagnostics. 

**Key message 5, section 1.3 Key messages:**

**In medical diagnostics for mold exposure, environmental monitoring of microbial volatile organic compounds (MVOCs) in indoor air has no indication.**

**3.5.5 Diagnostic methods without sufficient scientific evidence or without a medical or scientific basis**

Diagnostic methods are often demanded by patients without sufficient scientific evidence or without a medical or scientific basis, but are also propagated by doctors and therapists from various disciplines (e.g., [568]). It is striking that scientific methods that are justified for other issues, such as human biomonitoring, are used for purposes other than their intended purpose in order to give the procedure the appearance of being scientific. The same applies to the fanciful names of some of the methods used. Without criticizing individual procedures with the patients, their protection from expensive and nonsensical procedures must also be a concern of environmental medical advice. The right to a pluralism of schools of thought and procedures should remain unaffected by this [489]. 

The Commission “Methods and Quality Assurance in Environmental Medicine” of the RKI has commented in detail on various diagnostic methods without sufficient scientific evidence or without a medical-scientific basis in environmental medicine [350, 351, 352, 353, 355, 357]. 

Diagnostic methods without sufficient scientific evidence or without a medical or scientific basis must, like all medical methods, be tested and evaluated according to the current state of scientific knowledge [356]. In addition, unambiguous statements on these methods should be made by experts, professional societies and institutions in specialist journals and the lay press as well as in television programs. In addition, statutory and private health insurance funds should only cover the costs of diagnostic methods without sufficient scientific evidence or without a medical or scientific basis if their benefit has been proven [512]. 

Table 11 shows examples of diagnostic methods without sufficient scientific evidence or without a medical or scientific basis [5, 73, 232, 283, 350, 351, 352, 353, 355, 357, 512, 535, 684]. These are not recommended due to a lack of evidence. 

**Key message 25, section 1.3 Key messages:**

**The following diagnostic methods shall not be used for indoor mold exposure because there is insufficient scientific evidence (without claim of completeness): Detection of molds in the blood, determination of IgA antibodies directed against molds, determination of lymphocyte subpopulations, determination of cytokines, determination of oxidative stress, visual contrast sensitivity test (VCS test), tear film break-up time.**

**Key message 26, section 1.3 Key messages:**

**The following diagnostic methods shall not be used for indoor mold exposure due to a lack of medical and scientific evidence (list is non-exhaustive): Electroacupuncture according to Voll, bioresonance procedures, pendulum, Vega test, decoder dermography, biotonometry, biotensor, Kirlian photography (plasma print procedure, energetic terminal point diagnosis), regulation thermography according to Rost, auriculodiagnostics, kinesiology, aurascopy, iris diagnostics, cytotoxic blood tests, provocation and neutralization test (PN test).**

## 4 Therapy 

Even if the causal relationship between complaints/findings/diseases and the presence of mold/moisture in the interior cannot be proven, from a preventive and hygienic point of view, the first “therapeutic” measure in the presence of moisture/mold damage is rapid professional and proper remediation and, in the case of serious clinical pictures with a high health risk (immunosuppression according to the criteria of KRINKO [349], severe influenza, severe COVID-19, CF, asthma, rhinoconjunctivitis, rhinosinusitis) the immediate minimization of exposure. 

### 4.1 Allergological therapy 

**4.1.1 General drug treatment**

Depending on the organ-specific severity of the allergic disease, topical, and/or systemic therapy is generally indicated in the case of a mold allergy. 

The medical treatment of allergological conditions (rhinitis, conjunctivitis, sinusitis, bronchial asthma) associated with exposure to molds is no different from the treatment of other allergens (e.g., pollen). 

With regard to the (organ-related) drug therapy of an allergy, please refer to the relevant guidelines [69, 343, 545]. 

**4.1.2 Specific immunotherapy (hyposensitization)**

Specific immunotherapy (SIT) is the only therapy for allergies that has a causal immunomodulating function. The introduction of therapeutic allergens activates specific blocking antibodies, tolerance-inducing cells, and messenger substances. This leads to a reduced immune response triggered by allergens. As a result, the inflammatory reaction in the body responsible for the allergic symptoms decreases [545]. 

SIT with mold extracts should be used as early as possible in the course of the disease, especially if the measures of drug therapy and allergen avoidance have not previously led to a stabilization of the symptoms [14]. 

The corresponding mold allergens must be clearly confirmed diagnostically as triggers of the allergic symptoms. The prerequisite for SIT is evidence of allergen-specific IgE sensitization of clinical relevance. The combination of different test methods together with the medical history provides a sufficient basis for SIT. 

Hyposensitization requires a reliable diagnosis. The current guideline [545] is referred to. 

For testing for mold allergens (exclusively for prick testing), single and mixed allergens are only available from a few manufacturers, mainly for outdoor mold species. For guideline-based provocation prior to specific immunotherapy, only 12 single allergen extracts and one mixed allergen extract that comply with the Therapy Allergen Regulation are currently available (as of 1/23). However, these are not always commercially available due to production and supply bottlenecks. For hyposensitization, the hyposensitizing solution should preferably come from the same manufacturer as the test solution used. 

Preparations for subcutaneous immunotherapy (SCIT) and sublingual immunotherapy (SLIT) are available for hyposensitization. For SCIT, unmodified allergens are used as aqueous or physically coupled extracts and chemically modified extracts (allergoids) as semi-depot extracts. The predominantly unmodified allergen extracts for SLIT are used as aqueous solutions. For the rare mold allergens, there are no therapy allergens approved for SLIT or SCIT in accordance with the Therapy Allergen Regulation (http://www.pei.de). They cannot be mixed with the allergens that satisfy the Therapy Allergen Regulation. 

The SCIT injections are carried out by a doctor who has experience with this form of therapy and is qualified to treat allergy emergencies. Prior information and documentation is required (observe the Patient Rights Act and adhere to the guidelines). 

The individual success of hyposensitization can be monitored on the basis of the severity of the clinical symptoms. A symptom questionnaire has proven its worth. Regular checks using whole-body plethysmography are useful for asthmatics or those with corresponding symptoms. 

For allergies to spores of seasonal outdoor molds, evidence of clinical efficacy is limited to a few SCIT studies with *Alternaria* *alternata* and *Cladosporium herbarum* extracts [151, 277, 425]. A 3-year DBPC study in children with an allergy to *Alternaria* was able to demonstrate the efficacy of SCIT from the second year of treatment [371]. The difficulty in producing mold allergen extracts is that molds biologically produce different allergens *in vitro* (culture tanks) than in their spore form *in vivo* in which they are inhaled. To overcome this problem, a multicenter Spanish study involving 111 adolescents and adults shows promising results with SCIT using recombinant Alt a 1 [685]. A preparation with Alt a 1 polymer allergoids has been available on the German market since 2020. 

The effectiveness of SLIT for hyposensitization against indoor-relevant molds has not yet been sufficiently scientifically proven. Prior to AIT, the clinical relevance of an identified sensitization and the individual benefit/risk ratio should be carefully considered [60, 545, 611, 721]. 

Cross references:

- German Society for Allergology and Clinical Immunology e.V. (DGAKI). S2k guideline Allergen immunotherapy for IgE-mediated allergic diseases, version 5.0. Register number 061 - 004. Status: June 30, 2022. Valid until: June 29, 2027. https://register.awmf.org/de/leitlinien/detail/061-004
- *Pfaar O, Ankermann T, Augustin M, Bubel P, Böing S, Brehler R, Eng PA, Fischer PJ, Gerstlauer M, Hamelmann E, Jakob T, Kleine-Tebbe J, Kopp MV, Lau S, Mülleneisen N, Müller C, Nemat K, Pfützner W, Saloga J, Strömer K, Schmid-Grendelmeier P, Schuster A, Sturm GJ, Taube C, Szépfalusi Z, Vogelberg C, Wagenmann M, Wehrmann W, Werfel T, Wöhrl S, Worm M, Wedi B; Commenting participation and process support: Kaul S, Mahler V, Schwalfenberg A.* Guideline on allergen immunotherapy in IgE-mediated allergic diseases: S2K Guideline of the German Society of Allergology and Clinical Immunology (DGAKI), Society of Pediatric Allergology and Environmental Medicine (GPA), Medical Association of German Allergologists (AeDA), Austrian Society of Allergology and Immunology (ÖGAI), Swiss Society for Allergology and Immunology (SSAI), German Dermatological Society (DDG), German Society of Oto-Rhino-Laryngology, Head and Neck Surgery (DGHNO-KHC), German Society of Pediatrics and Adolescent Medicine (DGKJ), Society of Pediatric Pulmonology (GPP), German Respiratory Society (DGP), German Professional Association of Otolaryngologists (BVHNO), German Association of Paediatric and Adolescent Care Specialists (BVKJ), Federal Association of Pneumologists, Sleep and Respiratory Physicians (BdP), Professional Association of German Dermatologists (BVDD). Allergol Select. 2022; *6:* 167-232; doi: 10.5414/ALX02331E

### 4.2 Infectiological therapy 

**Systemic mycoses**

For the procedure for mold infections, please refer to the relevant guideline. 

Cross references:

- Onkopedia Guideline - Invasive fungal infection therapy. Status: 2019

Working Group on Infections in Hematology and Oncology (AGIHO) of the German Society for Hematology and Medical Oncology (DGHO) 

*Ruhnke M, Cornely OA, Schmidt-Hieber M, Alakel N, Boell B, Buchheidt D, Christopeit M, Hasenkamp J, Heinz WJ, Hentrich M, Karthaus M, Koldehoff M, Maschmeyer G, Panse J, Penack O, Schleicher J, Teschner D, Ullmann AJ, Vehreschild M, von Lilienfeld-Toal M, Weissinger F, Schwartz S*. Treatment of invasive fungal diseases in cancer patients - Revised 2019. Recommendations of the Infectious Diseases Working Party (AGIHO) of the German Society of Hematology and Oncology (DGHO). Mycoses 2020; *63:* 653-682; doi: 10.1111/myc.13082, for the Infections in Hematology and Oncology Working Party (AGIHO) of the German Society of Hematology and Medical Oncology (DGHO); https://www.onkopedia.com/de/onkopedia/guidelines/invasive-pilzinfektionen-therapie/@@guideline/html/index.html

### 4.3 Treatment methods without sufficient scientific evidence or without a medical or scientific basis 

Treatment methods without sufficient scientific evidence or without a medical or scientific basis are often demanded by patients, but also propagated by doctors and therapists from various disciplines (e.g., [568]. It is noticeable that scientifically based procedures such as antifungal treatment are misused in order to give the procedure the appearance of being scientific [489]. 

Table 12 shows examples of treatment methods without sufficient scientific evidence or without a medical or scientific basis [5, 73, 160, 384, 512, 762]. These treatments are not recommended due to a lack of evidence. 

Treatment methods without sufficient scientific evidence or without a medical-scientific basis must, like all medical methods, be tested and evaluated according to the current state of scientific knowledge and only be reimbursed as a health insurance benefit if the therapeutic benefit has been proven [512]. 

### 4.4 Exposure avoidance 

As with all allergic diseases, avoidance of exposure or allergens has priority. Nevertheless, timely medication is necessary so that the full-blown allergic disease does not reappear after a period with few symptoms. 

Eliminating the causes of dampness as the basis for mold growth indoors is the first priority. The evidence for the success of remediation measures after moisture or mold damage in relation to asthma and respiratory symptoms and the incidence of colds in adults and children is moderate [614] as shown in Table 13. 

Together with other studies [75, 253, 314, 367, 708], there is sufficient evidence that an intervention that improves housing conditions with respect to moisture and mold growth [624, 625] has a favorable effect on the morbidity of asthma and respiratory allergies. 

For people at increased risk (e.g., CF or immunosuppression), the recommended hygiene measures include avoiding damp rooms and rooms with mold growth and the need for mold remediation [11, 146]. 

By using special devices for air purification or dehumidification, the concentrations of airborne mold components (bioaerosols) can be reduced [50]. In Europe, filter classes H13-H14 are classified as HEPA. According to the US standard, the filter must remove at least 99.97% of all particles > 0.3 µm, comparable to filter class H13 according to EN 1822-1:1998. While HEPA filters were effective for cat and dog allergies, there was no evidence of effectiveness for house dust mite or mold allergies [782]. A meta-analysis of 10 randomized controlled trials of air filtration from 1973 to 1999 found a small statistically significant improvement in overall symptoms and sleep disturbances with the use of air purifiers, but no improvement in nasal symptoms, medication use, or peak expiratory flow (PEF) [439]. 

In addition to the clearly indicated, professional structural renovation to eliminate and prevent moisture and renewed microbiological growth in the interior (see below), for further technical measures (air filters, dehumidifiers), which are recommended in various ways [437, 682], only insufficient evidence is available due to a lack of studies [297]. 

“Air purifiers” with ionizers are to be classified as problematic, as they can lead to ozone pollution that is harmful to health [137, 282, 761]. 

Those affected should follow the recommendations listed in the following sections 4.4.1 and 4.4.2. 

**4.4.1 Indoor air**

Mold spores always occur in indoor air. As a rule – especially in summer – their concentration is primarily determined by the outdoor air, whereby the concentration of the typical outdoor air species or genera (extramural fungi such as *Cladosporium*, sterile mycelia, possibly yeasts, possibly *Alternaria*, possibly *Botrytis*) is usually lower in the indoor air than in the outdoor air, but is similarly dependent on the season as the concentration in the outdoor air. It is now generally known that the normal background concentration of certain mold species in indoor air differs significantly from that of outdoor air, especially in winter. The typical humidity indicator *Aspergillus versicolor* complex can occur with a background value of up to ~ 40 CFU/m^3^ in indoor spaces that are not exposed to humidity; it is very rare in outdoor air. The same applies to other intramural fungi (so-called indoor humidity indicators). In the case of moisture damage with microbial growth, the concentrations are often above these reference values [380]. 

Atypical outdoor air species or genera (intramural fungi such as fungal species with a high indication of moisture damage, e.g., *Acremonium* spp., *Aspergillus versicolor* complex, *A. penicillioides*, *A. restrictus*, *Chaetomium* spp., *Phialophora* spp., *Scopulariopsis brevicaulis*, *Scopulariopsis fusca*, *Stachybotrys chartarum*, *Tritirachium* (*Engyodontium*) *album*, *Trichoderma* spp.), on the other hand, are present in higher concentrations in indoor air than in outdoor air in the event of moisture/mold damage [297, 380]. During the remediation of such damage, increased mold spore concentrations sometimes occur indoors [138], which can also contaminate adjoining rooms if the sealing is inadequate. 

The most important prerequisite for the growth of mold fungi and other microorganisms is moisture. Indoors, the following causes, among others, can lead to increased moisture on component surfaces, in components or furnishings:

- Structural causes
- Usage-related causes
- Accidents

The following frequency distribution was determined [381]:

- Construction defects 45%
- Increased humidity 18%
- Incorrect furnishings 17%
- Leakages 20%

The nutrients required for the growth of mold fungi are present in sufficient quantities on almost all building surfaces, e.g., on wallpaper, wooden surfaces, silicone joints, but also in dust deposits on plaster and in the organic additives contained in plaster. Further information can be found in the “Guidelines for the prevention, detection and remediation of mold infestation in buildings” published by the German Environment Agency in 2017 [297]. In 5 – 20% of homes in Germany there is moisture damage or moisture/mold damage [63, 379, 518, 725]. 

The following measures prevent [135, 487] or reduced [135, 763] mold contamination in the interior:

- If there is a thermal bridge or if a building component is insufficiently insulated, a building expert should be consulted to develop a concept to eliminate the damage. The mediation itself must be carried out by a qualified specialist company (see chapter 5).
- If an interior room is ventilated by a technical ventilation system, it must be checked whether the resulting moisture is sufficiently removed (time, volume flow). The corresponding ventilation system must be maintained regularly in accordance with the manufacturer’s instructions and care must be taken to ensure a clean air supply.
- If conversion, furnishing and/or change of use measures are planned that fundamentally change the building physics (or: indoor climate) conditions of the building, the measure must first be checked by an appropriate specialist in order to prevent mold infestation. This applies in particular to the installation of new windows.
- The use of diffusion-tight building materials such as tiles, plastics, or special paints (especially if painted over several times) and wallpaper (vinyl wallpaper, glass fiber wallpaper) prevents the intermediate storage of moisture in the building components. The moisture condenses on the diffusion-tight surface. This often leads to mold infestation, especially if there are other structural and/or usage-related deficits. In interior rooms, only surfaces should be “sealed” with diffusion-tight materials where splashing water and a high level of moisture ingress (e.g., in wet rooms) is to be expected on a regular basis. In the case of new buildings or renovation work, care should be taken to ensure that the surfaces of building components are as open to diffusion as possible.
- Depending on the size of the property and the building materials used, several tons of water are brought into a solid new building. It usually takes 2 – 3 years for the moisture to dry out of the building. This drying process can be accelerated by technical drying. In the first 2 – 3 years, the property should be ventilated particularly well. If built-in furniture is to be installed in the property, special precautions must be taken. The built-in furniture must be ventilated [379] and the wall behind the built-in furniture should not be papered. In some cases, it may be advisable to lightly heat the relevant walls locally.
- The living area should be kept dry by ventilation (3 – 4 times a day for 5 – 15 minutes, preferably as cross ventilation) and heating. During the cold season, the relative humidity in living areas should not exceed 50%.
- Adequate ventilation, especially in wet rooms! Most moisture and therefore mold growth is found in bathrooms. Dark streaks along the tile joints are a sign of mold growth. Ventilate the bathroom sufficiently during and after showering or bathing. Drain and dry the shower tray and bathtub. Wash shower curtains regularly and allow to dry. If possible, dry wet towels and other laundry outside or in a designated, well-ventilated room. No carpets in the bathroom. Good air circulation between furniture and floor, ceiling and wall [379, 487].
- Do not use humidifiers, room fountains, etc. indoors during the cold season if the indoor air humidity is between 40 – 55% relative humidity. Humidifiers and indoor fountains must be maintained regularly in accordance with the manufacturer’s instructions.
- Do not store damp firewood in the interior.
- Do not dry laundry in the interior.
- Keep indoor spaces low in dust (mold allergens are dust-bound).
- Sanitation of sleeping areas as in the case of a mite allergy.
- Potted plants and cut flowers should be avoided indoors and under no circumstances should they be present in the sleeping area, as mold will always grow in the soil.
- Do not store damp shoes, clothes, or leather items in cupboards.
- Garbage cans, especially compost garbage cans, must be emptied and cleaned frequently.
- Fur-bearing animals, especially long-haired dogs, can be the cause of spores entering the interior. They can bring the spores collected in their fur from the outside air into the interior.
- Small animal feed and bedding must be stored in a dry place.
- With steam irons, remove the water properly between uses [583, 637].
- Special infection prevention measures must be observed for immunocompromised patients receiving outpatient treatment [349]. These must be discussed with the attending physician in accordance with the risk group in question.

Indoors in the vicinity of

- Nurseries (horticulture),
- Recycling facilities,
- Compost heap,
- Composting facilities,
- Waste collection and waste management facilities

can lead to increased exposure to mold (see chapter 4.4.2). This must be taken into account when ventilating the relevant rooms, especially in summer (*Alternaria alternata*) and fall (*Aspergillus fumigatus).*

**4.4.2 Outdoor air**

Increased mold concentrations in the outdoor air depend on the potency of the sources present. Some of these sources are independent of the vegetation, such as waste management companies, recycling and composting facilities, or gardening. Other sources depend on the vegetation, the weather, and the region. For example, certain mold species occur in association with plants such as grasses, herbs, or trees. Increased concentrations of these mold spores are emitted through activities during mowing/harvesting of these plants and processing of these plants into food or animal feed. Increased spore concentrations, especially of *Aspergillus fumigatus*, also occur when organic material decomposes. In the case of a pollinosis, seasonal symptoms may occur that do not correlate with the usual pollen seasons and this may also occur in the case of a mold spore allergy [140, 180]. 

The concentrations of airborne mold spores depend on the spore genus/family, weather conditions, and human activities (e.g., correlation of *Alternaria* and harvest season). 

**Figure 5:**
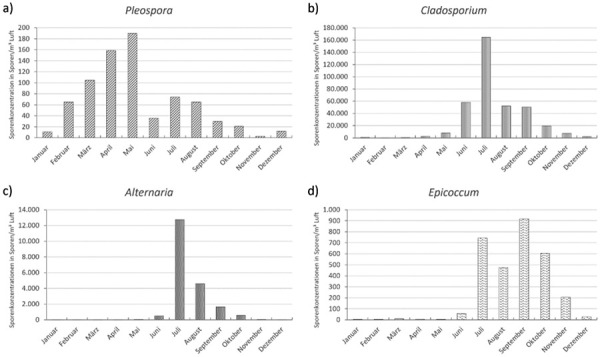
Monthly course of spore concentrations (in spores/m^3^ of air) in the outdoor air for several mold genera as an example for one year and one measuring station in Germany: a) *Pleospora* 2021 – Berlin measuring station, b) *Cladosporium* 2021 – Chemnitz measuring station, c) *Alternaria* 2021 – Berlin measuring station, d) *Epicoccum* 2021 – Berlin measuring station. Missing data at the Chemnitz station in the periods: February 24, 2021 to March 1, 2021 and April 1, 2021 to April 6, 2021. The figure was provided by the German Pollen Information Service Foundation (Stiftung Deutscher Polleninformationsdienst). The concentration was determined using volumetric spore traps based on the Hirst principle [269] followed by microscopic analysis of the samples. The measurement procedure is standardized [738].

For example, *Pleospora* spore concentrations are particularly high in spring (Figure 5a), *Cladosporium* and *Alternaria* in the summer months (Figure 5b, c), and *Epicoccum* in summer and early fall (Figure 5d). 

Spores can be in the air in any weather during the summer months. For example, the spore flight of the allergy-relevant mold genus *Alternaria* is concentrated on days with dry, warm weather [234]. In contrast, the occurrence of some other fungal genera, such as *Pleospora* or *Fusarium*, is more closely associated with previous precipitation and higher humidity [234]. 

Forecasts of the spore flight of some mold spores (*Alternaria, Cladosporium, Epicoccum, Pleospora*) in the outdoor air for Germany are part of the free weekly pollen forecast of the German Pollen Information Service Foundation (PID) [765], published on the PID website (https://www.pollenstiftung.de/). 

If there is a strong spore flight, people allergic to mold spores should limit their time outdoors if necessary, and it is recommended to minimize or avoid activities where there is an increased exposure to mold spores, e.g., gardening, handling bark mulch or rotten leaves, working on compost heaps, etc. 

The spore flight of *Alternaria* in particular is closely linked to the grain harvest [28, 513]. Spending time in the vicinity of farmed fields can lead to increased exposure to spores. 

People who suffer from a proven severe allergy to *Alternaria* should consider avoiding the outside air during midsummer or wearing a mouth and nose protection or FFP2 mask. 

Persons with moderate to very severe immunosuppression – this includes in particular hemato-oncology patients after stem cell transplantation or intensive chemotherapy – should avoid staying near compost heaps, composting facilities, market gardens, recycling facilities, waste management facilities and, if possible, avoid working in the garden or in agriculture/horticulture, as increased concentrations of *Aspergillus fumigatus* may occur. 

## 5 Renovation of living spaces (buildings) with moisture problems and mold growth 

Mold infestation of small to medium size (< 0.5 m^2^, only superficial infestation) with a known cause can often be removed by the affected person themselves, provided they are not allergic to mold or suffer from immunosuppression. In the case of larger infestations and low to medium infestations with no known cause, a specialist company should be involved. In principle, it is important to start mold remediation immediately so that the infestation does not spread [297]. 

Professional remediation of moisture/mold damage includes eliminating the physical cause(s) of the building, removing mold-infested materials in accordance with the recommendations of the UBA mold guide [297], drying and subsequent fine cleaning. The remediation must be carried out by a qualified specialist company, which must prove that the responsible persons in the company carrying out the work have specialist knowledge of occupational health and safety in accordance with the German Ordinance on Biological Substances (BioStoffV) and that their specialist area of responsibility has been extended to include the area of mold damage. This expertise is ensured as part of further training and is verified by means of a (regularly) agreed minimum standard of examination requirements in the subject areas of microbiological principles and assessment principles, building physics principles, physical-chemical measurement methods, hygiene and occupational health and safety, damage detection and remediation techniques as well as fine cleaning [726, 740], e.g., for:

- Academy of Engineers
- Professional Association of German Building Biologists VDB e.V.
- Bundesverband Ausbau & Fassade / Fachverband Ausbau & Fassade NRW
- Bundesverband für Schimmelpilzsanierung BBS e.V.
- German Wood and Building Protection Association e.V.
- Professional associations of the framework (www.schimmelnetz-nrw.de)
- Baden-Württemberg State Health Office
- NRW state network for mold consulting
- Netzwerk Schimmel e.V.
- Hamm Eco-Center
- TÜV Rheinland Akademie GmbH
- Verbraucherzentale NRW e.V.

For the protection of the workers and room users, the guidelines “Health hazards from biological agents during building renovation” [138] must be followed. It is important to work with as little dust as possible during all refurbishment measures in order to keep the spread of mold spores with the dust and via the air as low as possible. Damp cleaning (mopping) is therefore always preferable to dry vacuuming. When vacuuming, only appliances with additional filters (high efficiency particulate air filters such as HEPA filters) should be used. Sweeping should be avoided altogether, as this unnecessarily stirs up and spreads dust. Qualified fine cleaning after refurbishment is also particularly important. 

The details and procedures are not part of this guideline. Detailed information can be found in the UBA Guideline [297] and in the relevant literature [129, 130, 726, 740, 786, 787, 788]. 

The remediation of moisture/mold damage in interior spaces is not regulated by the state. The decision on the type and method of remediation should always be based on the results of accepted investigation methods for which there are generally accepted assessment values. The generally accepted recommendations for carrying out mold remediation [297, 786, 787, 788] must be followed. 

## 6 Risk of poverty and moisture/mold infestation 

**Risk of poverty**

- A person is considered to be at risk of poverty if they have to live on less than 60% of the median income of the population as a whole.
- The reasons and the degree of poverty risk vary greatly.
- Factors influencing the risk of poverty result from socio-economic status, which is determined by a person’s level of education, professional position and net income [378] and other factors such as [209]:
     - Migration experience
     - Gender affiliation
     - Old age (poverty in old age more common among women)
     - Caring for relatives in need of care (more often women)
     >- Number of underage children in the family
     - Marital status of parents of underage children

**Risk of poverty in Germany**

- Increased from 15% of the population in 2015 to 16.2% (~ 13.8 million people) in 2020 [71] and is expected to increase further due to the current rise in energy prices
- Is particularly high among women, people between the ages of 18 and 25, the unemployed, people with a low level of education, without graduation or a migrant background, as well as communities with several children and especially single parents [71]
- In the new federal states particularly affects younger people who had problems finding an apprenticeship and then a job after reunification [71]
- Mainly affects women in old age in the old federal states [72]
- Varies greatly from region to region depending on the urbanization and gross domestic product of the individual federal states [667, 671, 672]

**Frequency of moisture/mold infestation in homes in Germany**

- The proportion of homes in Germany with visible mold infestation is in the range of 5 – 20% [62, 381, 382, 518, 703]. The way in which such damage was assigned differed between the various investigations.
- Moisture/mold damage can be structural or usage-related. Another possible reason for such damage is an accident [381].
- It is estimated that hidden damage is also present in 14% of homes.
- In the period from 2009 to 2019, the Federal Statistical Office [672] reported moisture damage in the home more frequently in the population at risk of poverty compared to the rest of the population on the basis of self-assessment.
- According to a study by the RKI, mold in living spaces occurs more frequently in immigrants (10.4%) than in natives (4.4%) [213, 579].
- According to a study by the RKI, mold in living spaces occurs more frequently in people with a low social status (7.8%) compared to people with a high social status (3.3%) [213, 579].
- The evaluation of the German Environment Agency’s Children’s Environmental Survey 2003/06 revealed that:
     - for children with migration status (non-migrant → migrant)
     - for the area types (rural → suburban → urban),
     - for the size of the municipality (< 100,000 → ≥ 100,000 inhabitants),
     - for the house types (single-family house → two-family house → multi-family house → high-rise building/apartment block) and
     - on completion of the residential building (from 1995 → 1980 to 1995 → 1950 to 1979 → to 1949)

a significantly higher probability for the occurrence of moldy walls [703].

- The above-mentioned factors are generally inversely proportional to the rent level and the available living space in m^2^ per person.
- Comparable results to the aforementioned RKI study were reported across Europe by the WHO (2019) [139].

**Risk of moisture/mold damage due to exposure to poverty [** 430, 488]

- Due to sharply rising rents [209], the fact that the stock of social rental housing in Germany has been declining in recent years [209] and the fact that many households are financially overburdened by their housing costs [673], people who are at risk of poverty generally have little living space available per capita [669], which objectively increases the risk of high humidity and thus mold infestation in the home. These residential properties are often located in noisy areas [672], such as on busy roads, which makes it more difficult to ventilate optimally.
- People who are at risk of poverty usually live in rented accommodation [668], some of which are poorly maintained and inadequately repaired. Subsidized housing is also often built in regions with poorer environmental and housing quality, where vandalism [672], which means that the tenants’ motivation to treat the housing responsibly is not optimal. Due to the lack of experience and knowledge about appropriate living behavior [209], this effect is exacerbated.
- Due to the energy poverty often associated with the risk of poverty, the living space is not heated and ventilated efficiently, which increases the risk of mold [670]. The situation is exacerbated by the fact that people on low incomes often live in poorly insulated homes, which are very susceptible to mold damage due to the condensation of moisture on the cold exterior walls. The current sharp rise in energy prices will exacerbate this problem.
- Due to the lack of data, it is not possible to estimate the extent to which the health risk of people at risk of poverty is increased in relation to the population as a whole due to the occurrence of moisture/mold damage.

## 7 Prevention 

Increased exposure to mold spores should be avoided as a matter of principle. This applies in particular to immunocompromised patients [349], patients with severe influenza, severe COVID-19, CF, and asthma. They must be informed about the risks associated with indoor exposure to mold and about measures for prevention [112, 293], if necessary supplemented by home tests for the presence of *Aspergillus fumigatus* and *Aspergillus flavus* (only very rarely expected indoors) [580]. The attending physician must inform the patient of the results of these tests in relation to the specific individual circumstances. 

For all health disorders associated with exposure to environmental factors, the focus is on prevention and exposure avoidance. This also applies in particular to mold. An indoor climate that favors mold growth (high humidity, lack of ventilation) must always be avoided to prevent allergies [563]. 

Detailed information on avoiding moisture/mold damage due to construction defects (e.g., new building moisture, thermal bridges) and information on inappropriate usage behavior (e.g., heating and ventilation behavior) or accidents can be found in the UBA mold guide [297] and in the guidebook “Feuchtigkeit und Schimmelbildung – Erkennen, beseitigen, vorbeugen”. With regard to the new problems arising from the measures currently required to save energy from the consumer advice center [739]. 

With regard to the place of exposure, a distinction must be made between indoor and outdoor air (see chapter 4.4.1 and 4.4.2). 

In addition, the source strength must be differentiated between the various sources with regard to assessability:

- Defined source with an expected source strength
     - Moisture/mold damage
- Ubiquitous source that is partly dependent on lifestyle habits
     - Dealing with waste in the interior
     - Potted flowers
     - Humidifier, room fountain, aquarium
     - Fur-bearing animals; long-haired dogs in particular can be the cause of spores entering the interior
     - Small animal feed and bedding

People with proven asthmatic and allergic symptoms to mold spores, which occur frequently in the outdoor air, can try to minimize or avoid contact with their allergen, for example by using the PID weekly pollen forecast to find out about possible spore exposure so that they can then better avoid it, in particular by reducing the exposure time in the outdoor air or by wearing a mouth and nose protection (see chapter 4.4.2). 

The measures to prevent increased mold concentrations indoors can be found in section 4.4.1. 

With regard to the new problems arising from the measures currently required to save energy, refer to the BSS information sheet - “Energy crisis 10 tips for heating and ventilation at low room temperatures - Information sheet - Reduce heating and avoid mold” [https://bss-schimmelpilz.de/energiekrise-heizen-und-lueften-niedrige-raumtemperatur/] for information on the energy-saving measures that are currently necessary. 

Cross references:

- German Society for Allergology and Clinical Immunology e.V. (DGAKI) and German Society for Pediatric and Adolescent Medicine e.V. (DGKJ). S3 Guideline Allergy prevention, version 4.0. Register number 061 - 016. Status: December 07, 2021. Valid until: January 01, 2026 (under revision). https://register.awmf.org/de/leitlinien/detail/061-016
- Onkopedia Guideline - Prophylaxis of invasive fungal infections. Status: 2022

Working Group on Infections in Hematology and Oncology (AGIHO) of the German Society for Hematology and Medical Oncology (DGHO) 

*Stemler J, Mellinghoff SC, Khodamoradi Y, Sprute R, Classen AY, Zapke SE, Hoenigl M, Krause R, Schmidt-Hieber M, Heinz WJ, Klein M, Koehler P, Liss B, Koldehoff M, Buhl C, Penack O, Maschmeyer G, Schalk E, Lass-Flörl C, Karthaus M, Ruhnke M, Cornely OA, Teschner D.* Primary prophylaxis of invasive fungal diseases in patients with haematological malignancies: 2022 update of the recommendations of the Infectious Diseases Working Party (AGIHO) of the German Society for Haematology and Medical Oncology (DGHO), 2022. J Antimicrob Chemother. 2023; *78:* 1813-1826; doi: 10.1093/jac/dkad143, for the Infections in Hematology and Oncology Working Party (AGIHO) of the German Society for Hematology and Medical Oncology (DGHO); https://www.onkopedia.com/de/onkopedia/guidelines/invasive-pilzinfektionen-therapie/@@guideline/html/index.html

## Funding 

The guideline was produced without any financial support. 

## Conflict of interest 

See Table 14. 

**Risk matrix 1.**
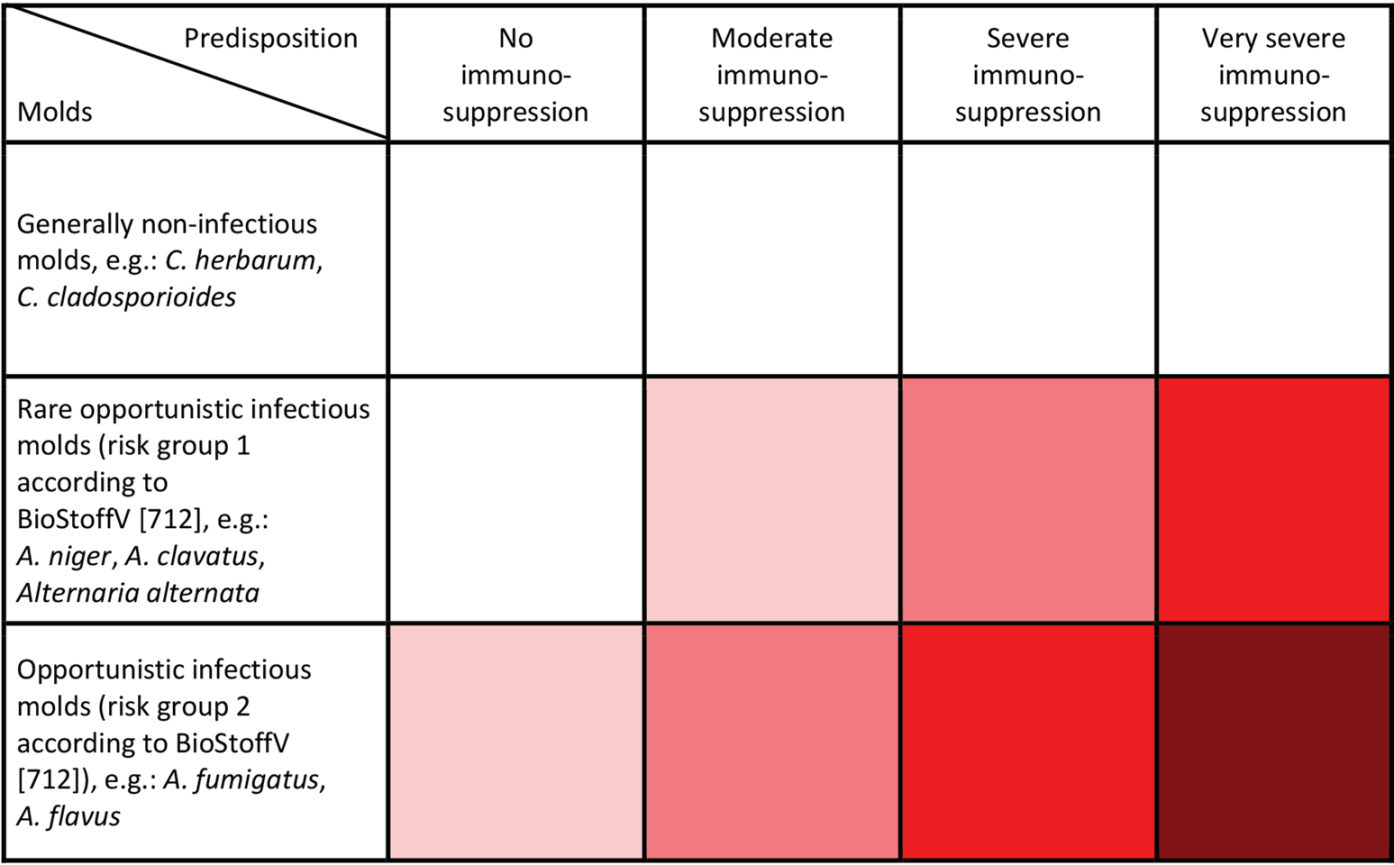
Risk of infection by mold (The darker a box, the greater the possible health risk.).

**Risk matrix 2.**
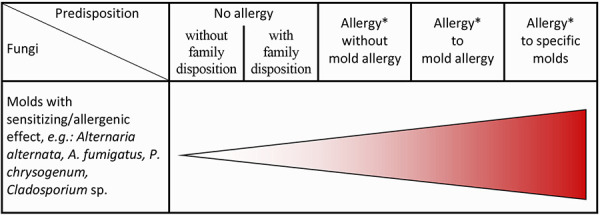
Risk of sensitization/risk of allergy caused by mold (the darker the color, the greater the possible health risk). *Proof of the clinical relevance of a sensitization determined in the allergy test required!

**Table 2.** Important sources of frequently occurring mold species and genera (examples from practical investigations).

**Species or genus**	**Important sources**
*Cladosporium herbarum, Alternaria alternata, Botrytis cinerea*	Vegetation, outdoor air-associated
*Aspergillus versicolor *complex and *Scopulariopsis brevicaulis*	Damp plaster
Xerophilic *Aspergillus *species of the *Restrictus* complex (esp. *A. penicillioides *and *A. restrictus) *as well as *Aspergillus glaucus/pseudoglaucus *and *A. montevidensis*, *Wallemia sebi*	Cellulose-containing materials with only slightly increased moisture
*Aspergillus fumigatus*	Composting, rotting of plant material, indicator for thermotolerant processes
*Chaetomium *spp., *Trichoderma *spp., *Phialophora *spp.	Colonizer of damp wood and cellulose materials such as cardboard
*Penicillium *species	Perishable food, waste, organic waste and wallpaper, house dust
*Sarocladium strictum, Parengyodontium album, Lecanicillium psalliotae *and *Simplicillium *spp*.*	Polystyrene floor insulation with prolonged moisture penetration
*Stachybotrys chartarum, Acremonium *spp, *Chaetomium *spp.	Very moist, cellulose-containing building materials
*Wallemia sebi*, *Aspergillus restrictus *complex	Keeping caged animals with bedding

**Table 3.** Examples of species complexes based on molecular analyses (modified according to [297]).

**Complex or group**	**Selected species**	**Molecular markers for differentiation**
*Aspergillus versicolor *complex	*A. versicolor, A. amoenus, A. creber, A. jensenii, A. protuberus, *et al.	CaM, β-Tub
*Aspergillus niger *complex	*A. niger, A. acidus, A. aculeatus, A. brasiliensis, A. tubingensis, *et al.	CaM, β-Tub
*Aspergillus fumigatus *complex	*A. fumigatus, A. lentulus, A. novofumigatus, A. fumigatiaffinis, *et al.	CaM, β-Tub (Note: Cultivation at 37 °C; Differentiation *A. lentulus)*
*Fusarium solani *complex	*F. solani, F. keratoplasticum, F. petroliphilum, F. lichenicola, *et al.	EF-1α, RPB-1 and/or RPB-2

**Table 4.** Evidence for the relationship between indoor moisture/mold exposure and disease (in alphabetical order), modified from [7, 39, 45, 58, 59, 74, 78, 79, 80, 101, 108,111, 116, 119, 131, 133, 162, 169, 175, 181, 182, 183, 201, 202, 204, 211, 212, 245, 263, 273, 275, 287, 289, 291, 298, 303, 316, 319, 322, 338, 363, 370, 394, 437, 442, 444, 445, 449, 450, 451, 462, 465, 466, 467, 490, 499, 501, 504, 509, 514, 520, 521, 522, 525, 526, 528, 532, 534, 537, 540, 546, 547, 552, 553, 568, 569, 596, 598, 632, 635, 642, 643, 644, 645, 652, 655, 694, 698, 717, 722, 742, 746, 754, 756, 757, 777, 783, 790, 791, 794, 797].

**Causal relationship **	
Sufficient evidence for an association^1^	Allergic bronchopulmonary aspergillosis (ABPA) Allergic bronchopulmonary mycoses (ABPM) Allergic respiratory diseases Allergic rhinitis Allergic rhinoconjunctivitis Aspergilloma Aspergillosis Aspergillus bronchitis Asthma (manifestation, progression, exacerbation) Bronchitis (acute, chronic) Community-acquired aspergillus pneumonia Favoring respiratory infections Hypersensitivity pneumonitis (HP); extrinsic allergic alveolitis (EAA) Invasive aspergillosis Mycoses Organic dust toxic syndrome (ODTS) (workplace) Pulmonary aspergillosis (subacute, chronic) Rhinosinusitis (acute, chronic invasive or granulomatous, allergic)
Limited or suspected evidence of an association	Atopic eczema / atopic dermatitis / neurodermatitis (manifestation) Chronic obstructive pulmonary disease (COPD) Mood disorders Mucous membrane irritation (MMI) Odor effects Sarcoidosis
Inadequate or insufficient evidence for an association	Acute idiopathic pulmonary hemorrhage in children Airborne mycotoxicosis Arthritis Autoimmune diseases Cancer Chronic fatigue syndrome (CFS) Endocrinopathies Gastrointestinal effects Multiple Chemical Sensitivity (MCS) Multiple sclerosis Neuropsychological effects Neurotoxic effects Sudden infant death syndrome Renal effects Reproductive disorders Rheumatism Sick building syndrome (SBS) Teratogenicity Thyroid diseases Urticaria

^1^The diseases listed here can be subsumed under the term Building Related Illness (BRI), even though BRI requires that the etiology, pathology, pathophysiology, diagnosis, treatment, prevention and prognosis are clearly known [429, 663].

**Table 5.** Examples of typical antigens and antigen sources for hypersensitivity pneumonitis (HP; extrinsic allergic alveolitis (EAA) (mod. after Costabel et al. 2020 [113]).

**Antigen**	**Antigen source**	**HP type**
Bacteria		
Thermophilic actinomycetes	Moldy hay and straw	Farmer’s lung
	Sugar cane dust	Bagassosis
* Klebsiella oxytoca*	Humidifier	Humidifier lung
Mycobacteria		
* Mycobacterium avium *complex	Whirlpools	Whirlpool lung (hot tub lung)
* Mycobacterium immunogenum*	Cooling lubricants	Machinist’s lung
Mushrooms		
* Absidia corymbifera*	Moldy hay and straw	Farmer’s lung
* Trichosporon cutaneum*	Interiors	Summer-type alveolitis
* Penicillium roqueforti*	Cheese production/industrial sources	Cheese washer lung
* Purpureocillium lilacinum, Aspergillus* spp.	Wind instruments	Wind instrument alveolitis
Animal proteins		
Feathers and excrement	Birds	Bird keeper lung
Serum and urine	Rats	Rat protein alveolitis
Plant proteins		
Nut dust	Tiger nuts	Tigernut alveolitis
Soy dust	Soy products	Soy dust alveolitis
Wood dust	Various wood dusts	Woodworker’s alveolitis
Enzymes		
Phytase	Animal feed production	Phytase alveolitis
Enzymes from *Bacillus subtilis*	Biological cleaning agent	Detergent lung

**Table 6.** Diagnostic criteria for allergic bronchopulmonary aspergillosis.

a) Diagnostic criteria for allergic bronchopulmonary aspergillosis according to Rosenberg et al. [586]:
**Main criteria:**
1. Bronchial asthma
2. Positive immediate reaction in the skin test for *Aspergillus fumigatus*
3. Total IgE > 417 IU/mL
4. Positive specific IgE against *Aspergillus fumigatus*
5. IgG antibodies against *Aspergillus fumigatus*
6. Blood eosinophilia (> 1,000 Eos/µL)
7. Central bronchiectasis
8. Radiological volatile or permanent pulmonary infiltrates
**Secondary criteria**
Tough mucus plugs
Positive sputum culture for *Aspergillus fumigatus*
Late reaction in the intradermal test for *Aspergillus fumigatus*
b) Modified ISHAM diagnostic criteria for allergic bronchopulmonary aspergillosis 2021 [616]:
Combination of criteria with best sensitivity/specificity:
1. Bronchial asthma
2. *Aspergillus fumigatus*-specific IgE > 0.35 kU/L
3. Total IgE > 500 IU/mL
and at least two of the following criteria:
1. *Aspergillus fumigatus*-specific IgG > 27 mgA/dL
2. Bronchiectasis in CT thorax
3. Eosinophilia in the blood count > 500/µL

**Table 7.** Mold mycoses and their pathogens [712].

Infectious disease (invasive mycosis)	Pathogen (risk group according. TRBA 460 (2016) [712]
Aspergillosis	*A. fumigatus* (2)
	*A. flavus* (2)
	*A. niger* (complex) (1, 2)
	*A. terreus* (2)
	*A. nidulans* (1)
Mucormycosis	*Rhizopus oryzae* (2)
	*Mucor* sp. (1)
	*Rhizomucor* (1)
Phaeohyphomycoses	*Curvularia *sp. (1)
	*Bipolaris* sp. (1)
	*Alternaria* sp. (1)
Hyalohyphomycoses	*Fusarium* sp. (1, 2)
	*Pseudallescheria* sp.= *Scedosporium* sp. (2)
Penicillioses	*Talaromyces* (formerly *Penicillium*) *marneffei* (2)

**Table 8.** Differential diagnosis of extrinsic allergic alveolitis (EAA) and organic dust toxic syndrome (ODTS) [354].

Features	EAA	ODTS
Exposure	Various allergens	Endotoxins, high exposure
Incidence	2 – 30 / 10,000	10 – 100 / 10,000
Latency	4 – 8 hours	4 – 12 hours
Auscultation	End-expiratory rales on both sides basal	Normal, possibly rattling noises
Lung function	Restriction (rarely obstruction, D_LCO_ reduced	Normal (possible restriction)
Precipitins	Often specific IgG	Mostly negative

**Table 9.** Immunosuppression risk groups of the Commission for Hospital Hygiene and Infection Prevention (KRINKO) at the Robert Koch Institute [349].

**Risk group 1 (moderate immunosuppression/deficiency)**
– Granulocytopenia < 0.5 × 10^9^ /L; (< 500/µL) probably up to 10 days (analogous to leukopenia < 1 × 10^9^ /L; < 1,000/µL), – Autologous stem cell transplantation up to 3 months after day 0 (day of stem cell return) – Lack of CD4-positive T helper cells < 200/µL (Caveat: age-appropriate normal values in children) – Autologous stem cell transplantation up to 3 months after intensive therapy phase *Patients who have more than one characteristic of the immunosuppression/deficiency listed under risk group 1 are assigned to risk group 2.*
**Risk group 2 (severe immunosuppression/deficiency)**
– Granulocytopenia < 0.5 × 10^9^ /L (< 500/μL) for more than 10 days (analogous to leukopenia < 1× 10^9^ /L; < 1,000/μL) – Severe aplastic anemia or macrophage activation syndrome during intensive immunosuppressive therapy – Allogeneic bone marrow or stem cell transplantation up to 6 months after completion of the intensive therapy phase (important: extent of GVHD and ongoing iatrogenic immunosuppression) – Acute inpatient treatment phase for autologous stem cell transplantation or after solid organ transplantation (until discharge)
**Risk group 3 (very severe immunosuppression/deficiency)**
– Allogeneic BMT/PBSCT in intensive therapy phase (until engraftment = regeneration of granulopoiesis) – Severe GVHD grade III or IV under intensive immunosuppression *The decision to assign patients after allogeneic stem cell transplantation to group 3 is ultimately made by the treating hematologist-oncologist after reviewing all findings.*

GVHD = graft-versus-host-disease = graft-versus-host-reaction; BMT = mone marrow transplantation; PBSCT = peripheral blood stem cell transplantation.

**Table 1.** Medline search on the topic of the guideline (as of 12-2014 and 6-2022).

**Keywords**	**Number of publications found ** **Status 12-2014 [** 777 **]**	**Number of publications found ** **Status 6-2022**
Indoor mo(u)ld or indoor dampness and human health	1,949	3,145
Indoor mo(u)ld or indoor dampness and human health and allergy	1,875	3,126
Indoor mo(u)ld asthma	440	805
Indoor mo(u)ld health asthma	285	564
Indoor mo(u)ld human health asthma		494
Indoor mo(u)ld allergy		1,198
Indoor mo(u)ld health allergy	434	774
Indoor mo(u)ld human health allergy		689
Indoor mo(u)ld atopy		89
Indoor mo(u)ld health atopy	24	59
Indoor mo(u)ld human health atopy		55
Indoor mo(u)ld arthritis		8
Indoor mo(u)ld health arthritis		4
Indoor mo(u)ld human health arthritis		3
Indoor mo(u)ld rheumatism		5
Indoor mo(u)ld health rheumatism		4
Indoor mo(u)ld human health rheumatism		3
Mo(u)ld arthritis rheumatism	46	62
Indoor mo(u)ld arthritis rheumatism		2
Indoor mo(u)ld health arthritis rheumatism		2
Indoor mo(u)ld human health arthritis rheumatism		1
Indoor mo(u)ld infection		750
Indoor mo(u)ld health infection	74	435
Indoor mo(u)ld human health infection		357
Indoor mo(u)ld irritation		113
Indoor mo(u)ld health irritation	42	95
Indoor mo(u)ld human health irritation		85
Indoor mo(u)ld symptoms		1,159
Indoor mo(u)ld health symptoms	449	724
Indoor mo(u)ld human health symptoms		594
Indoor mo(u)ld ergosterol		50
Indoor mo(u)ld health ergosterol	24	36
Indoor mo(u)ld human health ergosterol		27
Indoor mo(u)ld review		450
Indoor mo(u)ld health review		301
Indoor mo(u)ld human health review	160	266
Indoor mo(u)ld trial		67
Indoor mo(u)ld health trial		40
Indoor mo(u)ld human health trial	17	32
Indoor mo(u)ld clinical diagnosis	89	194
Indoor mo(u)ld health clinical diagnosis		113
Indoor mo(u)ld human health clinical diagnosis		93
Indoor mo(u)ld diagnostic / diagnostics		800
Indoor mo(u)ld health diagnostic / diagnostics	273	449
Indoor mo(u)ld human health diagnostic / diagnostics		353
Indoor mo(u)ld prevention		654
Indoor mo(u)ld health prevention	216	433
Indoor mo(u)ld human health prevention		358
Indoor mo(u)ld treatment		650
Indoor mo(u)ld health treatment	229	360
Indoor mo(u)ld human health treatment		293
Indoor mo(u)ld therapy		507
Indoor mo(u)ld health therapy	196	291
Indoor mo(u)ld human health therapy		250
Indoor mo(u)ld air filter		174
Indoor mo(u)ld health air filter	54	94
Indoor mo(u)ld human health air filter		55

**Table 10.** Stage I - IV after conjunctival provocation test (CPT) [235].

Stage I	Foreign body sensation, reddening of the conjunctiva, onset of itching
Stage II	As I, plus lacrimation, more intense itching, reddening of the conjunctiva tarsi of the lower eyelid
Stage III	As II, additionally reddening of the conjunctiva tarsi of the upper eyelid, severe itching, blepharospasm
Stage IV	As III, additionally chemosis, eyelid swelling, irresistible itching

**Table 11.** Selection of diagnostic methods without sufficient scientific evidence or without a medical or scientific basis in environmental medicine [5, 73, 232, 283, 350, 351, 352, 353, 355, 357, 512, 535, 684].

**Diagnostic methods without sufficient scientific evidence**	
Stress tests in body media	e.g., molds in the blood
Allergological examinations	e.g., serial dilution titration, cytotoxic blood tests, determination of IgG and IgA antibodies directed against molds in type I allergies
Investigations into disorders of the immune system	e.g., lymphocyte stimulation test, determination of lymphocyte subpopulations, determination of cytokines
Investigations of the oxidative system	e.g., determination of oxidative stress
Ophthalmological examinations	e.g., visual contrast sensitivity Test (VCS test)
**Diagnostic methods without a medical or scientific basis**	
Holistic or bioenergetic diagnostic procedures	e.g., electro-acupuncture according to Voll, bioresonance procedures, pendulum, Vega test, decoder dermography, biotonometry, biotensor, Kirlian photography (plasma print procedure, energetic terminal point diagnosis), regulation thermography according to Rost, auriculodiagnostics, kinesiology, aurascopy, iris diagnostics
“Clinical ecology” procedure	e.g., cytotoxic blood tests, provocation and neutralization test (PN test)

**Table 12.** Examples of treatment methods without sufficient scientific evidence or without a medical or scientific basis in environmental medicine [5, 73, 160, 384, 512, 762].

**Treatment methods without sufficient scientific evidence**
Antifungal treatment not in line with guidelines
Detoxification therapy, e.g., with cholestyramine (CSM therapy)
Dietary changes
Homeopathic treatments
Symbiosis control
**Treatment methods without a medical or scientific basis**
Bioresonance therapy (Moratherapy)
Autologous blood and urine treatment
Holistic intestinal cleansing
Salt therapy
Clinical ecology procedures (e.g., provocation and neutralization test (PN test))

**Table 13.** Cochrane EBM Review by Sauni et al. (2011) [614] on the success of remediation measures after moisture or mold damage in relation to asthma and respiratory symptoms as well as the frequency of colds in adults and children.

**Measure**	**Effect on adults**	**Effect on children**
Home renovation (Evidence level: moderate)	Wheezing (asthma): OR 0.64 (KI: 0.55 – 0.75) Rhinitis: OR 0.57 (CI: 0.55 – 0.66)	Acute treatments (mean difference): MD –0.45 (KI: –0.76 – –0.14)

**Table 14.** The authors’ declarations of conflicts of interest and their assessment are presented below:

	**Consultant or expert activity**	**Collaboration in a scientific advisory board**	**Paid lecturing or training activities**	**Paid authors or co-author-ship**	**Research projects / conducting clinical studies**	**Proprietary interests (patent, copyright, share ownership)**	**Indirect interests**	**Topics of the guideline affected by COI; classification of the conflict of interest (IC) with regard to relevance, consequence**	
Dr. med. Ute Aurbach	No	No	No	No	No	No	No	Classification: no IC Consequence: none	
Priv.-Doz. Dr. med. Sven Becker	No	Yes	Yes	No	Yes	No	Board member AeDA	Classification: moderate IC; consequence: no voting authorization for allergological topics	
Prof. Dr. med. Romuald Bellmann	Yes	Yes	Yes	No	No	No	Mandate holder ÖGMM	Classification: moderate IC; consequence: no eligibility for antifungal therapy	
Prof. Dr. med. Karl-Christian Bergmann	Yes	Yes	Yes	No	No	No	Mandate holder DGP	Classification: moderate IC; consequence: not eligible to vote on asthma-related issues	
Prof. Dr. med. Oliver A. Cornely	Yes	Yes	Yes	No	No	No	No	Classification: moderate IC; consequence: no voting rights for mycosis-related topics	
Prof. Dr. med. Steffen Engelhart	No	No	No	No	No	No	Mandate holder GHUP and DGKH, Member of the Board GHUP	Classification: no IC; Consequence: none	
Dr. rer. nat. Guido Fischer	No	No	No	No	No	No	No	Classification: no IC; Consequence: none	
Dr. rer. nat. Thomas Gabrio	No	No	No	No	No	No	No	Classification: no IC; Consequence: none	
Dr. med. Birger Heinzow	No	No	No	No	No	No	No	Classification: no IC; Consequence: none	
Prof. Dr. med. Caroline E.W. Herr	No	No	No	No	No	No	Mandate holder and President of the GHUP	Classification: no IC; Consequence: none	
Dr. rer. nat. Julia Hurraß	No	No	No	No	No	No	Mandate holder and board member of the GHUP	Classification: no IC; Consequence: none	
Dr. med. Marcus Joest	No	No	Yes	No	No	No	No	Classification: low IC; consequence: no management function (coordination/AG management)	
Prof. Dr. med. Christian Karagiannidis	Not specified	Not specified	Not specified	Not specified	Not specified	Not specified	Not specified	Classification: not possible; consequence: not entitled to vote	
Prof. Dr. med. Ludger Klimek	No	Yes	Yes	No	Yes	No	Mandate holder AeDA	Classification: moderate IC; consequence: no voting rights for immunotherapy	
Dr. rer. nat. Martin Köberle	No	No	Yes	No	No	No	Mandate holder DDG	Classification: low IC; consequence: no management function (coordination/AG management)	
Dr. rer. nat. Annette Kolk	Not specified	Not specified	Not specified	Not specified	Not specified	Not specified	Not specified	Classification: not possible; consequence: not entitled to vote	
Dr. med. Dipl.-Chem. Herbert Lichtnecker	Not specified	Not specified	Not specified	Not specified	Not specified	Not specified	Not specified		Classification: not possible; consequence: not entitled to vote
Dr. med. Thomas Lob-Corzilius	No	No	No	No	No	No	Mandate holder GPAU		Classification: no IC; Consequence: none
Norbert Mülleneisen	No	No	No	No	No	No	Mandate holder DGP		Classification: no IC; Consequence: none
Prof. Dr. med. Dennis Nowak	No	No	Yes	No	No	No	DGAUM and DGP elected representatives		Classification: low IC; consequence: no management function (coordination/AG management)
Dr. med. Uta Rabe	No	No	Yes	No	No	No	Mandate holder AeDA		Classification: low IC; consequence: no management function (coordination/AG management)
Prof. Dr. rer. nat. Monika Raulf	No	No	Yes	No	No	No	Mandate holder DGAKI and DGAUM		Classification: low IC; consequence: no management function (coordination/AG management)
Prof. Dr. med. Jörg Steinmann	No	No	Yes	No	No	No	No		Classification: low IC; consequence: no management function (coordination/AG management)
Prof. Dr. med. Jens-Oliver Steiß	No	No	Yes	No	No	No	Mandate holder BAPP		Classification: low IC; consequence: no management function (coordination/AG management)
Dr. med. Jannik Stemler	No	No	Yes	No	No	No	No		Classification: low IC; consequence: no management function (coordination/AG management)
Dr. med. Ulli Umpfenbach	No	No	No	No	No	No	Board member BAPP, WAPPA, AGAS, FAAK		Classification: no IC; Consequence: none
Dr. rer. nat. Kerttu Valtanen	Not specified	Not specified	Not specified	Not specified	Not specified	Not specified	Not specified		Classification: not possible; consequence: not entitled to vote
Dr. rer. nat. Sandra Walser-Reichenbach	No	No	No	No	No	No	Mandate holder GHUP		Classification: no IC; Consequence: none
Dr. rer. medic. Barbora Werchan	No	No	No	No	No	No	No		Classification: no IC; Consequence: none
Prof. Dr. med. Gerhard A. Wiesmüller	No	No	No	No	No	No	Mandate holder and Board member GHUP		Classification: no IC; Consequence: none
Prof. Dr. med. Birgit Willinger	No	Yes	Yes	No	No	No	Mandate holder DMykG		Classification: low IC; consequence: no management function (coordination/AG management)

## Supplemental material

Supplemental material Supplemental material
