# Supplementary material for: AWMF mold guideline “Medical clinical diagnostics for indoor mold exposure” – Update 2023 AWMF Register No. 161/001
Source: Allergol Select. 2024 May 3;8:90–198. doi: 10.5414/ALX02444E (PMC11097193; doi:10.5414/ALX02444E)
Supplement: Supplemental material [file allergologieselect-8-090-S01.pdf]

Supplement

Definitions

Atopic patients

Persons with a tendency to hypersensitivity reactions such as allergic rhinitis, allergic asthma, atopic dermatitis, etc. to react to contact with environmental substances.

Bioaerosol

Airborne particles of biological origin (DIN EN 13098);

all accumulations of particles in the airspace to which fungi (spores, conidia, hyphal fragments), bacteria, viruses and/or pollen as well as their cell wall components and metabolic products (e.g., endotoxins, mycotoxins) adhere or which contain or form them (VDI 4253 Sheet 2).

Colony

A (mostly circular) network of branched hyphae with multiple, genetically identical nuclei is considered a single organism (CFU).

Complex

In this guideline, the term “complex” is used colloquially to refer to various species from the *Aspergillus* section *Versicolores* (e.g., *A. versicolor*, *A. jensenii*, *A. protuberus*). Species that are morphologically indistinguishable or difficult to distinguish from each other and whose molecular distinction has not yet been conclusively clarified are referred to as complex. In contrast, species that are well defined molecularly are grouped together as a section (species group between the rank of genus and species). The term “section” therefore represents a separate rank in taxonomy; the “complex” is not defined in the taxonomic ranking.

Endotoxins

Components of the lipopolysaccharides of the outer cell membrane of gram-negative bacteria. Endotoxins are released by living gram-negative bacteria by splitting off vesicles or when gram-negative bacteria die.

Exposure

Latin: exponere - to expose

Intentional or unintentional contact or exposure of the organism or its substructures (tissues, cells, molecules) to external influences, such as biological, physical, chemical, psychological, or other environmental influences.

House dust

House dust is the collective term for sedimented particulate and fibrous emissions in closed rooms. It is a mixture of various inorganic and organic substances, which also depends on the respective living conditions (e.g., the presence of a pet) and use. More detailed information can be found in the withdrawn VDI 4300 Sheet 8 [737].

Interior

Room that is protected from the weather.

In this guideline, indoor space refers to residential interiors as well as non-industrially used residential-like interiors, such as offices, kindergartens, and schools.

Moisture (dampness)

Visible, measurable, or perceived effect of excess water content that leads to problems in buildings such as leakage, material degradation, mold, mildew odor or directly measured excess moisture (in terms of rela-

tive humidity or water content) or microbial growth.

Moisture damage

Visible, measurable, or perceived consequence of increased water content in interior spaces or building components.

Mold (British English: mould)

(1) Superficial structures of molds visible to the naked eye (without taxonomic significance); (2) All types of microscopic fungi that grow in the form of cell filaments - so-called hyphae - as a fungal network (mycelium) and usually form pigmented conidia or sporangia carriers.

Mold contamination

Contamination of surfaces or materials (e.g., with fungal spores) that goes beyond the general basic contamination due to entry from outside (e.g., in house dust, approach spores).

Mold growth

Process involving biological activity, i.e., associated with moisture and characterized by cell division, hyphae, mycelium and possibly spore formation, etc.

Mold infestation/mold-infested materials

Building material or inventory that was or still is overgrown (colonized) with molds. If not already visible to the naked eye, determination by microscopic detection of a hyphal network and more or less developed conidia or sporangia carriers, regardless of whether the molds are still vital / active or already dead. In addition to molds, other biological substances such as bacteria may be present.

Molds

Collective term for hyphal and usually also spore-forming small fungi.

Water content (moisture)

(1) Water vapor partial pressure,

(2) Water content in a matrix, such as soil or building material.

Water damage

Visible, measurable, or perceived consequence of large quantities of water (accidents, leaks).
